# Supplementary material for: YAP silencing by RB1 mutation is essential for small-cell lung cancer metastasis
Source: Nat Commun. 2023 Sep 22;14:5916. doi: 10.1038/s41467-023-41585-z (PMC10516997; doi:10.1038/s41467-023-41585-z)
Supplement: Supplementary file 1 — Supplementary Information [file 41467_2023_41585_MOESM1_ESM.pdf]

Supplementary Figure 1

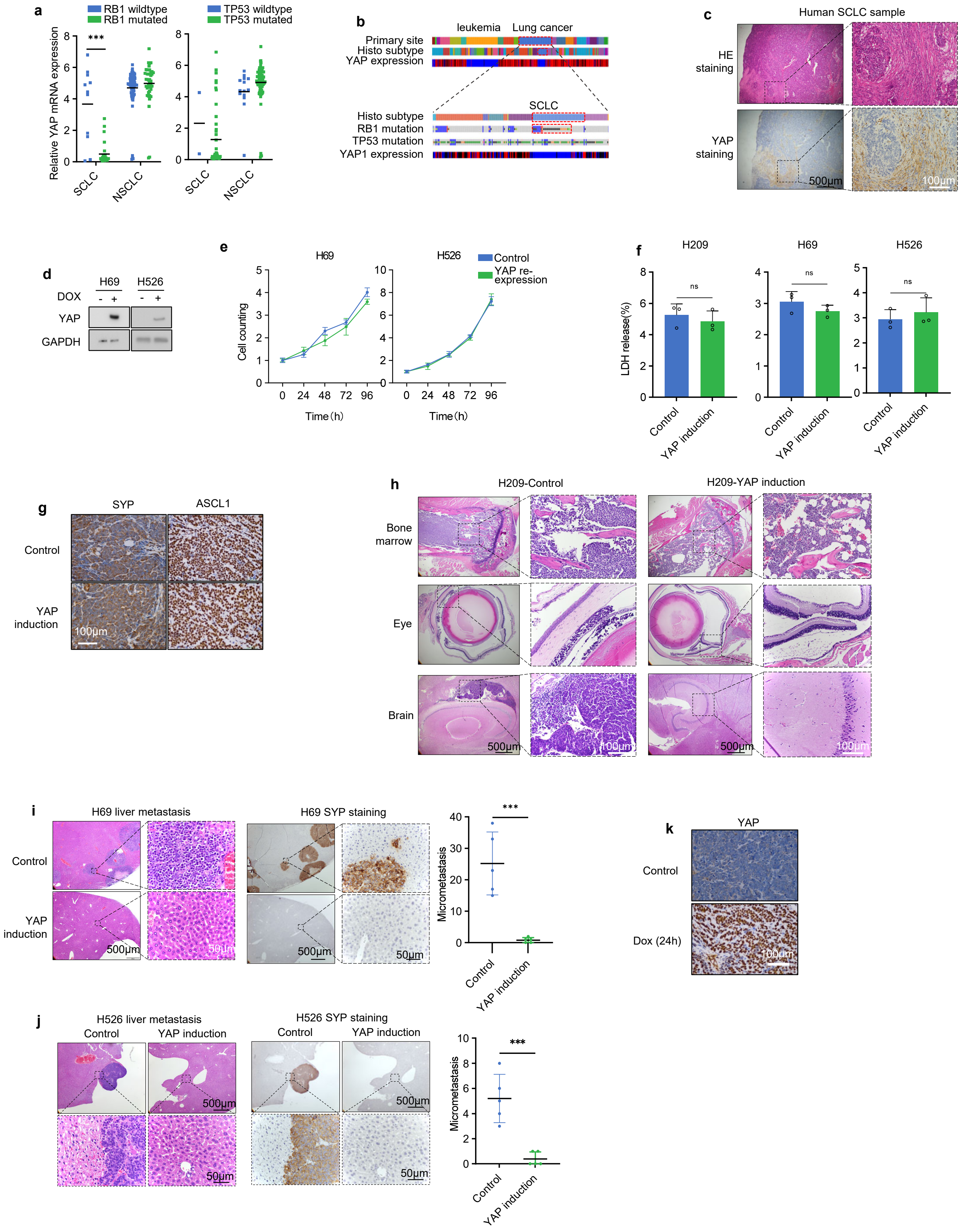

Supplementary Figure 1

***YAP* is silenced in SCLC and re-expression inhibits tumor metastasis.**

a. *RB1* but not *TP53* mutation is associated with low *YAP* expression in SCLC but not NSCLC cell lines.
b. Bioinformatics analysis of *YAP* expression from the Cancer Cell Line Encyclopedia database. The red dashed boxes in “Primary site,” “Histo subtype,” and “*RB1* mutation” mark lung cancer, SCLC, and *RB1* mutation, respectively. *RB1* WT (grey bars) and mutated (colored bars) are denoted. *YAP* expression is shown from red (high) to blue (low).
c. YAP staining of human SCLC tumor samples. YAP staining is absent in SCLC cells but present in neighboring non-SCLC cells.
d. Inducible *YAP* expression in H69, and H526 cells. DOX denotes the presence of 100 ng/ml Dox in culture.
e. *YAP* induction does not affect the growth of H69 or H526 cells. Viable cells were determined by the CCK8 assay.
f. Cell death is not affected by *YAP* induction in H209, H69 and H526 cells. Cell death was determined by LDH release assay.
g. *YAP* induction does not affect SCLC markers. Primary tumors from control and Dox-treated mice were stained for SYP and ASCL1.
h. *YAP* induction inhibits H209 cell metastasis in bone marrow, eyes, and brain.
i. *YAP* induction inhibits H69 liver metastasis. Liver from vehicle and Dox-treated mice were stained with hematoxylin and eosin (HE; left panel) or against SYP (middle panel). Quantification of liver metastasis (foci per mouse) is shown in the right panel.
j. *YAP* induction inhibits H526 liver metastasis. Liver from vehicle and Dox-treated mice were stained with hematoxylin and eosin (HE; left panels) or against SYP (middle panels). Quantification of metastasis (foci per mouse) is shown in the right panels.
k. IHC shows *YAP* induction in primary tumors from Dox-treated mice.
Source data are provided as a Source data file.

Supplementary Figure 2

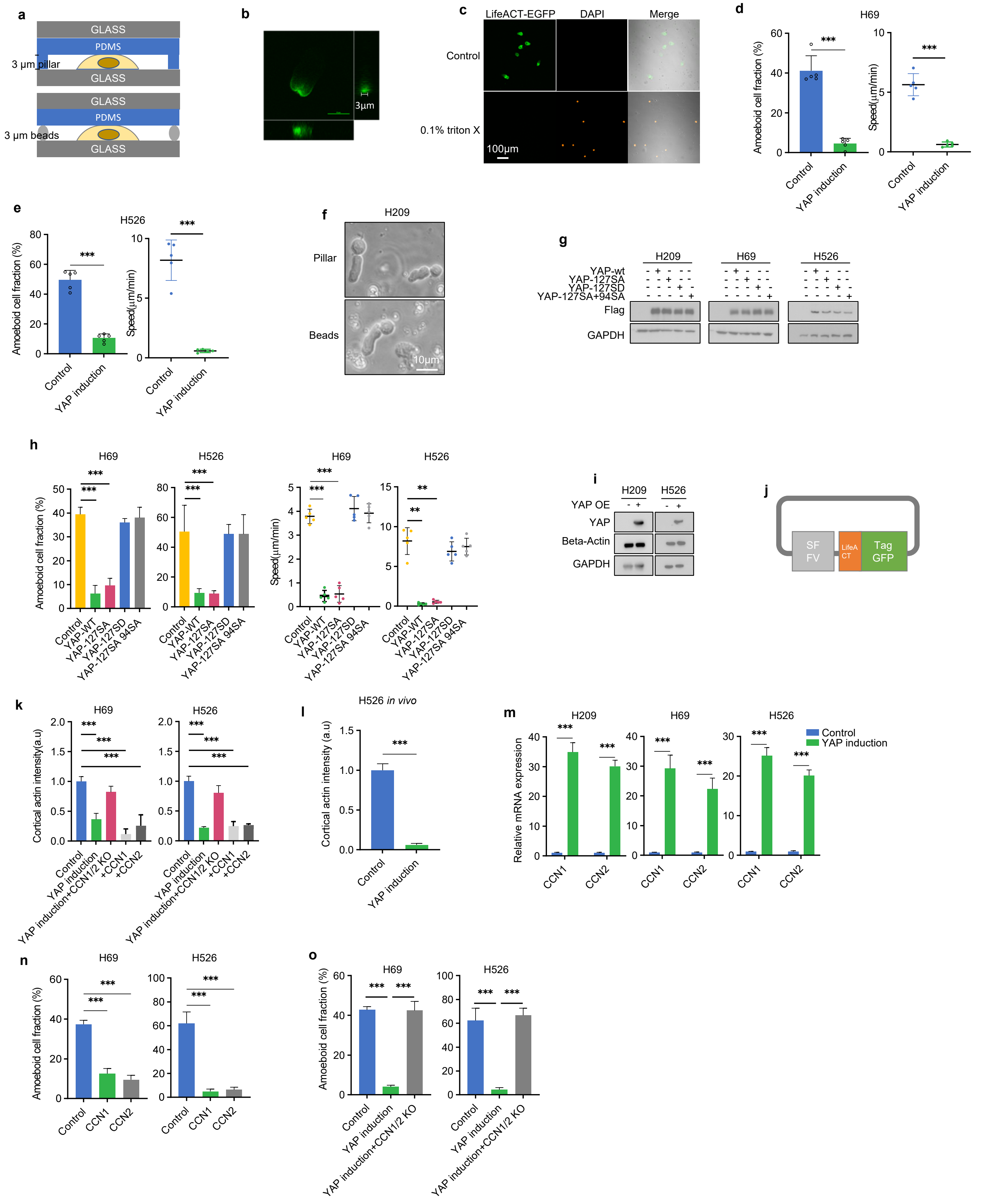

Supplementary Figure 2

YAP inhibits SCLC amoeboid migration through CCN1/2

a. Diagram of cell confinement settings. The confinement height was determined by 3 μm beads or microfabricated pillars.
b. 3D reconstruction showing the height of confinement.
c. Cell viability was not adversely affected by confinement. Trion-X treated cells are used as the positive control.
d. *YAP* expression inhibits the fast amoeboid-like migration of H69 cells under confinement.
e. *YAP* expression inhibits the fast amoeboid-like migration of H526 cells under confinement.
f. Amoeboid morphology of H209 cells under confinement using beads and pillar.
g. Ectopic expression of different *YAP* mutants in H209, H69 and H526 cell lines.
h. TEAD-dependent YAP co-transcriptional activity is required to suppress H69 and H526 amoeboid migration.
i. *YAP* re-expression does not alter Actin protein level.
j. Diagram of Lifeact-TagGFP that binds specifically to F-actin but not G-actin.
k. The effect of YAP and CCN1/2 on F-actin polarization. Fluorescence images of Lifeact-TagGFP are shown for H69 and H526 cells under confinement.
l. *YAP* re-expression decreases F-actin intensity in H526 xenografted tumors.
m. *CCN1* and *CCN2* are upregulated in YAP-induced H209, H69 and H526 cells.
n. Purified CCN1 or CCN2 protein inhibits amoeboid migration in H69 and H526 cells.
o. CCN1/2 are required for YAP to inhibit H69 and H526 cell amoeboid migration.
Source data are provided as a Source data file.

Supplementary Figure 3

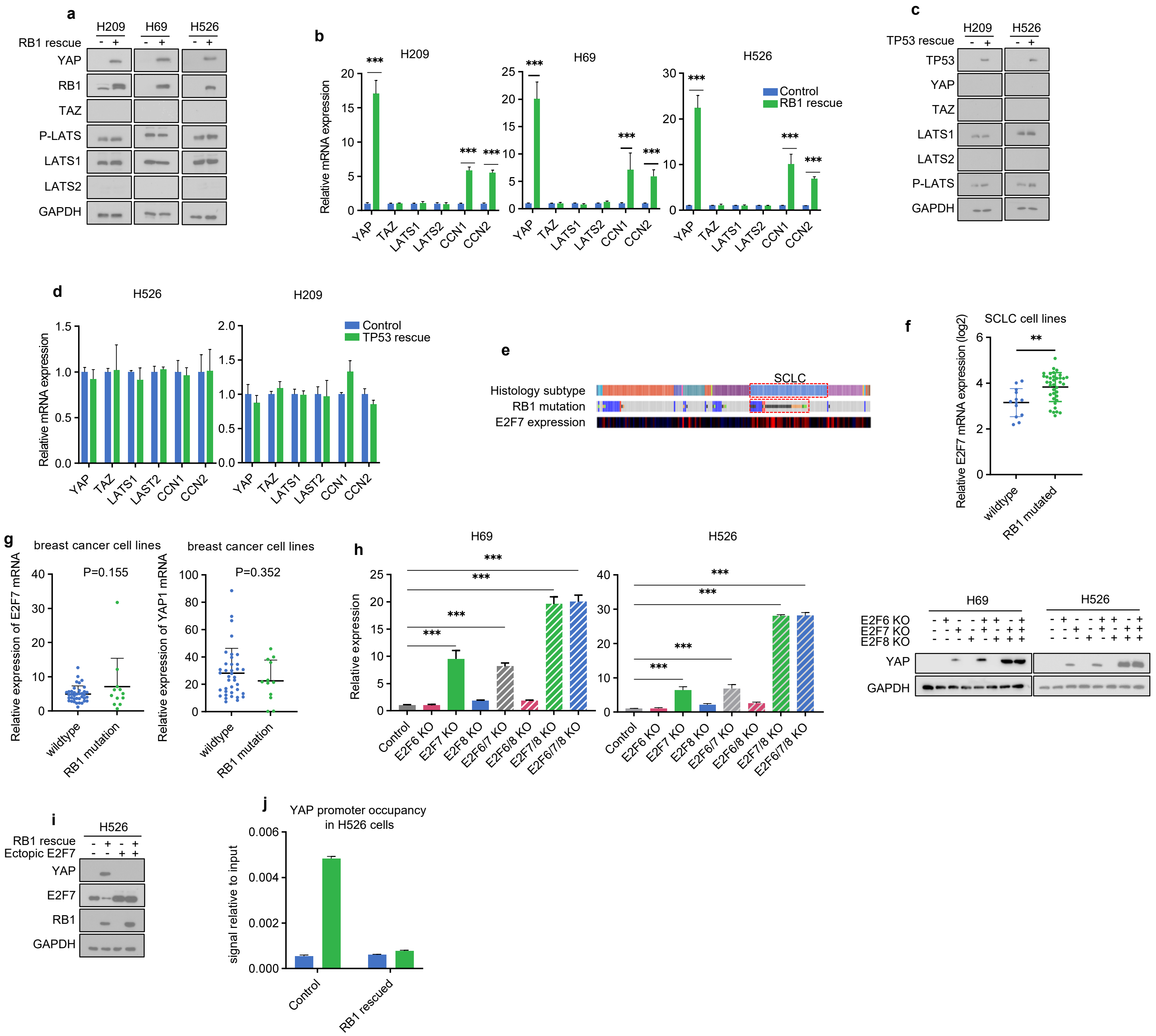

Supplementary Figure 3

RB1 **loss results in** ***YAP*** **downregulation**

a. Ectopic *RB1* expression induces YAP protein in H209, H69, and H526 cell lines.
b. Ectopic *RB1* expression induces *YAP* and its target genes *CCN1*/*2* in H209, H69, and H526 cell lines.
c. Ectopic *TP53* expression does not induce YAP protein in H209 and H526 cell lines.
d. Ectopic *TP53* expression does not induce *YAP* and its target genes *CCN1*/*2* in H526 and H209 cell lines.
e. Bioinformatics analysis shows that *E2F7* mRNA is elevated in *RB1* mutant SCLC cell lines (data from Cancer Cell Line Encyclopedia).
f. Correlation of *RB1* mutation and *E2F7* expression in SCLC cell lines.
g. *RB1* mutation does not correlate with *E2F7*/*YAP* expression in breast cancer cell lines.
h. *E2F7/8* KO induces *YAP* mRNA and protein in H69 and H526 cells.
i. Ectopic *E2F7* expression blocks YAP induction by RB1 in H526 cells.
j. RCOR1/2/3 and E2F7/8 KO increases H3K4me3 in the H526 cell *YAP* promoter. ChIP and RT-qPCR show *YAP* promoter H3K4 trimethylation.
Source data are provided as a Source data file.

Supplementary Figure 4

a RCOR1 CoIP-MS analysis

Proteins	scores
O60341|KDM1A	409.44
Q9UKL0|RCOR1	364.91
Q9UBW7|ZMYM2	363.8
O95071|UBR5	387.06
Q14687|GSE1	316.56
Q92769|HDAC2	298.5
Q13547|HDAC1	288.98
Q9P2K3|RCOR3	225.66
**Q96AV8|E2F7**	295.39
Q96BD5|PF21A	202.99
Q9NP66|HM20A	187.69
O75362|ZN217	186.11
Q14202|ZMYM3	180.13
Q9P0W2|HM20B	160.92
Q8IZ40|RCOR2	139.78

b

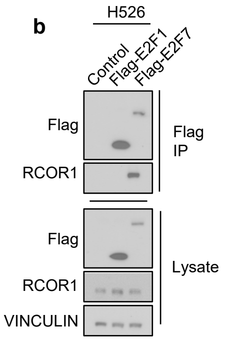

c

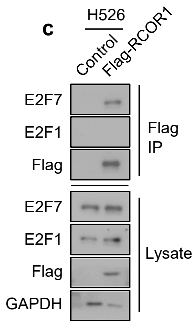

d

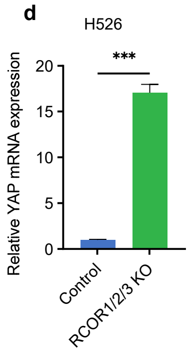

e

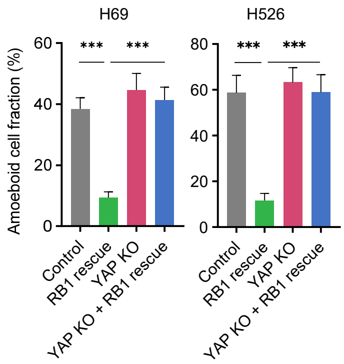

f

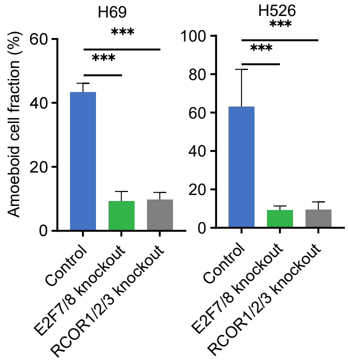

g

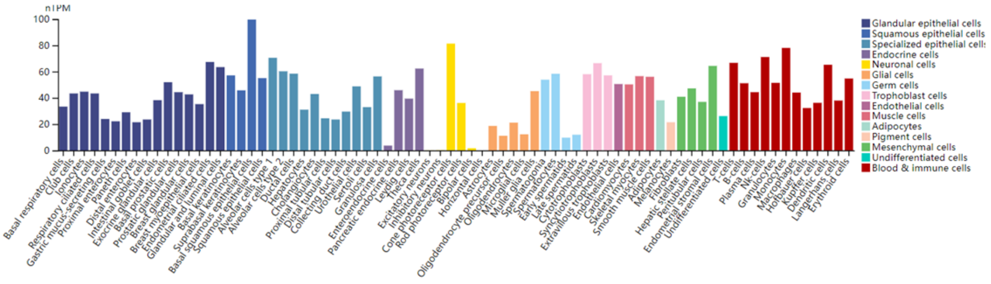

Supplementary Figure 4

E2F7 and RCOR mediate the effect of *RB1* loss on *YAP* downregulation

a. Mass spectrometry showing Flag-RCOR1 interacting proteins from H526 cells. The 15 top enriched proteins are listed.
b. E2F7 but not E2F1 interacts with RCOR1. Co-immunoprecipitation (IP) experiment confirmed the interaction between Flag-tagged E2F family proteins and endogenous RCOR1 in H526 cells.
c. RCOR1 interacts with E2F7 but not E2F1. Co-IP showed the interaction between Flag-RCOR1 and endogenous E2F family proteins in H526 cells.
d. *RCOR1/2/3* KO induces YAP mRNA in H526 cells.
e. YAP is required for RB1 to inhibit amoeboid migration in H69 and H526 cells.
f. *E2F7/8* KO or *RCOR1/2/3* KO inhibits amoeboid migration in H69 and H526 cells.
g. *REST* expression in different cell types. Data are from Protein Atlas single cell RNA-seq database.
   Source data are provided as a Source data file.

Supplementary Figure 5

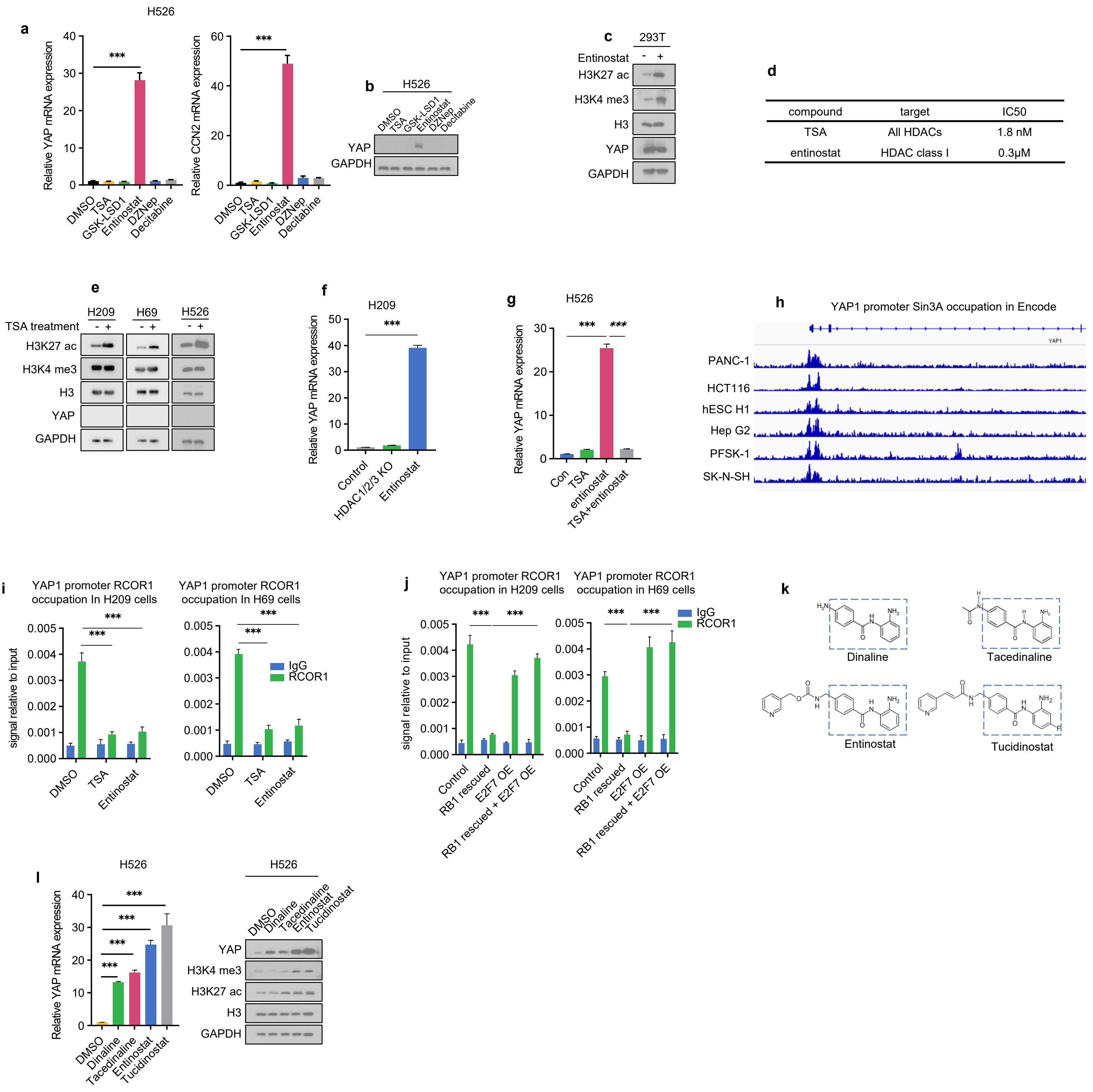

Supplementary Figure 5

Benzamide family HDAC inhibitors induce *YAP* transcription via the SIN3A-HDAC complex

a. Entinostat induces *YAP* and *CCN2* expression. H526 cells were treated with 1 μM entinostat, 0.3 μM TSA, 1 μM GSK-LSD1, 1 μM DZNep, and 10 μM decitabine for 24 hours.
b. 1μM entinostat treatment for 24 hours induces YAP protein in H526 cells.
c. Entinostat increases global H3K27 acetylation and H3K4 trimethylation but not *YAP* expression in 293T cells.
d. Specificity and half-maximal inhibitory concentration (IC_{50}) of HDAC inhibitor TSA and entinostat.
e. TSA elevates global H3K27 acetylation but not *YAP* expression in H209, H69 and H526 cells.
f. 1 μM entinostat for 24 hours but not *HDAC1*/*2*/*3* KO induces *YAP* in H209 cells.
g. TSA prevents entinostat from inducing *YAP* expression in H526 cells..
h. SIN3A occupancy on the *YAP* promoter. Data are from the ENCODE database.
i. Both TSA and entinostat treatment decrease RCOR1 binding to the *YAP* promoter in H209 and H69 cells.
j. *E2F7* expression blocks the ability of RB1 to decrease RCOR1 binding to the *YAP* promoter in H209 and H69 cells.
k. Chemical structures of benzamide family HDAC inhibitors.
l. Benzamide HDAC inhibitors induce *YAP* mRNA (left panel) and protein (right panel) in H526 cells.
   Source data are provided as a Source data file.

Supplementary Figure 6

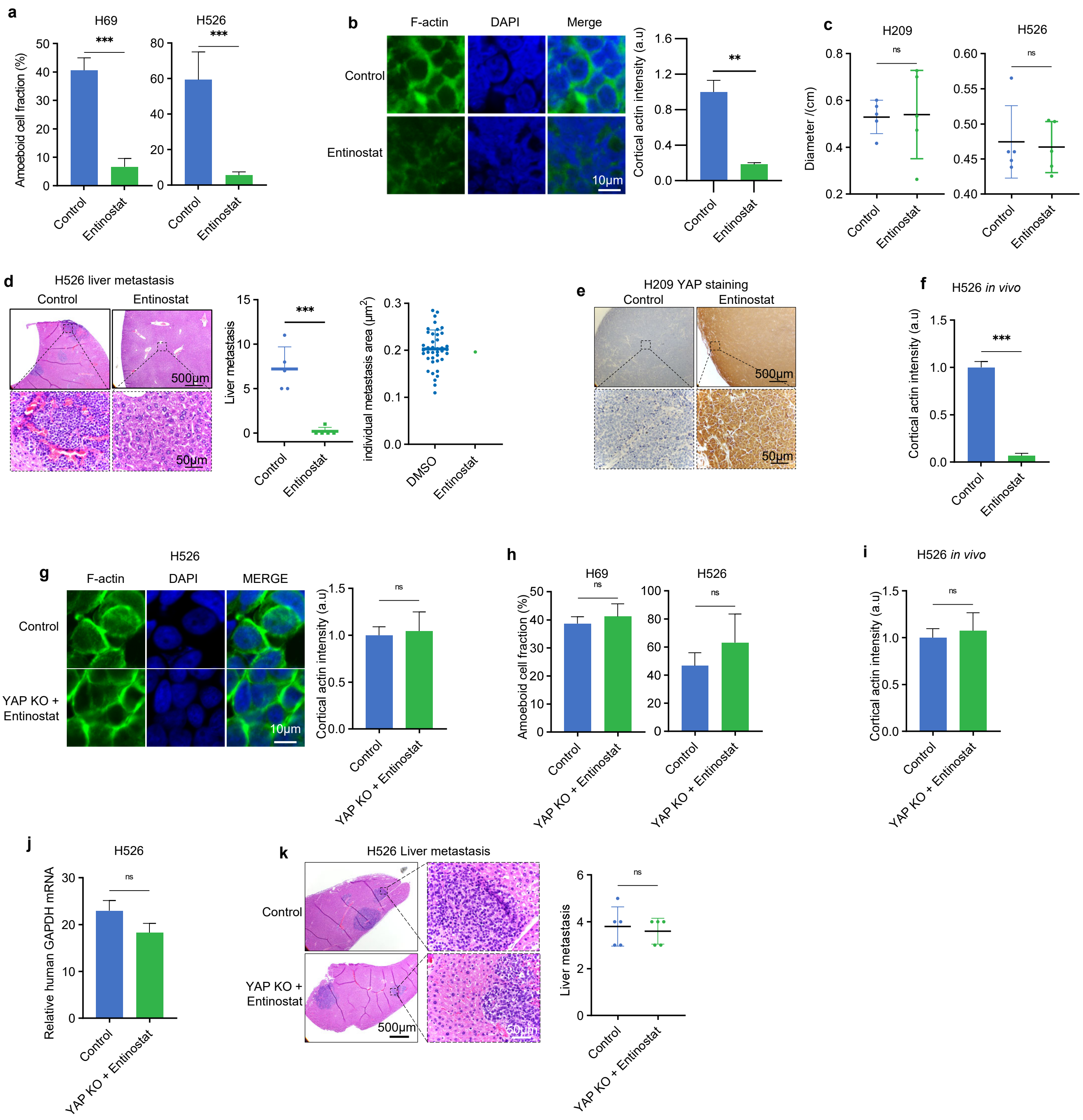

Supplementary Figure 6

Entinostat inhibits SCLC metastasis

a. Entinostat (1μM for 24 hours) inhibits H69 and H526 cells amoeboid migration under confinement.
b. Entinostat decrease H526 cells F-actin intensity *in vitro.* The fluorescence image and quantification of Lifeact-TagGFP are shown in the left and right panels, respectively.
c. Entinostat has no effect on the growth of primary tumors from H209 and H526
d. Entinostat inhibits liver metastasis of orthotopically grafted H526 cells. HE staining shows liver metastasis. Quantification of liver metastasis sites per mouse is shown in the right panel
e. Entinostat upregulates YAP protein in the H209 orthotopic model.
f. Entinostat treatment decreases F-actin intensity in H526 xenografted tumors.
g. Entinostat acts through YAP to decrease H526 cell F-actin intensity in vitro. The fluorescence image and quantification of Lifeact-TagGFP are shown in the left and right panels, respectively.
h. YAP is required for entinostat to inhibit H69 and H526 cell amoeboid cell migration.
i. Entinostat acts through YAP to inhibit F-actin in the H526 orthotopic model.
j. YAP KO blocks the inhibitory effect of entinostat on CTCs in H526 cells.
k. YAP is required for entinostat to inhibit H526 liver metastasis.

Source data are provided as a Source data file.

Supplementary Table 1: E2F7 massspectrometry							
Accession	-10lgP	Coverage (%)	Coverage (%) Sample 1	Area Sample 1	#Peptides	#Unique	scores in background
Q96AV8|E2F7_HUMAN	331.98	85	85	5.41E+10	222	217	0
Q13813|SPTN1_HUMAN	279.32	54	54	9.23E+08	126	125	288.66
Q01082|SPTB2_HUMAN	275.18	69	69	1.29E+09	154	132	276.8
P60709|ACTB_HUMAN	270.65	94	94		69	0	233.62
P63261|ACTG_HUMAN	270.65	94	94		69	0	0
P04264|K2C1_HUMAN	270.46	80	80	5.24E+09	75	61	248.98
P11142|HSP7C_HUMAN	264.82	77	77	2.48E+09	61	44	197.26
P13645|K1C10_HUMAN	261.23	68	68	2.54E+09	67	56	244.6
O60341|KDM1A_HUMAN	256.25	80	80	1.15E+09	61	61	0
P07437|TBB5_HUMAN	255.38	87	87	1.80E+09	65	10	195.24
P52732|KIF11_HUMAN	254.64	66	66	1.49E+09	70	70	241.48
O43795|MYO1B_HUMAN	249.85	58	58	6.27E+08	67	60	187.29
P35579|MYH9_HUMAN	248.47	43	43	2.00E+08	75	62	218.67
P68371|TBB4B_HUMAN	247.87	87	87	3.07E+06	61	2	190.1
Q13885|TBB2A_HUMAN	247.18	87	87	9.59E+06	61	3	0
Q9BVA1|TBB2B_HUMAN	246.67	87	87	7.23E+07	61	2	186.85
O14744|ANM5_HUMAN	246.6	77	77	8.79E+09	85	83	229.76
P35527|K1C9_HUMAN	246.27	84	84	1.87E+09	56	55	250.52
Q71U36|TBA1A_HUMAN	245.06	84	84		49	0	187.01
P68363|TBA1B_HUMAN	243.7	84	84		51	0	193.37
Q9BQE3|TBA1C_HUMAN	241.91	84	84	2.72E+07	48	2	186.14
Q9Y230|RUVB2_HUMAN	238.75	84	84	1.11E+09	41	41	187.7
P19338|NUCL_HUMAN	236.89	71	71	2.90E+09	69	69	201.3
O75366|AVIL_HUMAN	236.71	73	73	1.08E+09	54	51	195.86
P78527|PRKDC_HUMAN	235.84	31	31	1.86E+08	97	96	167.85
Q9UM54|MYO6_HUMAN	234.94	50	50	5.15E+08	61	59	208.07
Q13509|TBB3_HUMAN	234.44	82	82		51	0	176.25
P20929|NEBU_HUMAN	232.49	14	14	3.48E+08	85	85	282.23
P04350|TBB4A_HUMAN	231.85	77	77	9.74E+05	46	2	176.62
P21333|FLNA_HUMAN	228.61	37	37	2.02E+08	72	66	236.33
P35908|K22E_HUMAN	228.45	79	79	1.09E+09	60	44	259.57
P25705|ATPA_HUMAN	225.74	67	67	3.93E+08	36	35	195.16
tr|A0A0B4J269|A0A0B4J269	223.89	45	45	2.80E+05	49	1	172.53
Q08211|DHX9_HUMAN	222.6	35	35	1.99E+08	41	41	228.71
O00159|MYO1C_HUMAN	221.92	52	52	2.39E+08	55	51	206.89
P09327|VILI_HUMAN	221.72	73	73	4.98E+08	49	46	166.91
Q14558|KPRA_HUMAN	221.18	84	84	4.29E+08	30	27	190.56
P11021|GRP78_HUMAN	220.21	62	62	3.42E+08	39	37	177.07
P05141|ADT2_HUMAN	220.17	89	89	2.87E+08	35	17	120.62
O60256|KPRB_HUMAN	218.56	80	80	1.38E+09	31	28	191.27
P04259|K2C6B_HUMAN	216.59	53	53	2.53E+06	38	1	162.17
P68032|ACTC_HUMAN	216.47	69	69		45	0	181.44
P68366|TBA4A_HUMAN	215.89	83	83	8.18E+07	45	8	175.43
P08779|K1C16_HUMAN	215.77	76	76	2.09E+08	37	22	155.46
Q9Y5B9|SP16H_HUMAN	214.6	50	50	2.40E+08	51	49	154.01
Q13748|TBA3C_HUMAN	213.91	62	62		33	0	176.79

Q15208|STK38_HUMAN	213.47	70	70	1.78E+09	41	34	202.28
P62736|ACTA_HUMAN	212.92	66	66		41	0	0
P63267|ACTH_HUMAN	212.92	66	66		41	0	174.85
P36578|RL4_HUMAN	211.81	57	57	1.09E+09	46	46	191.61
Q9BQA1|MEP50_HUMAN	211.73	96	96	2.78E+09	33	33	197.23
P0DMV8|HS71A_HUMAN	210.94	73	73	2.67E+08	43	27	129.36
P0DMV9|HS71B_HUMAN	210.94	73	73	2.67E+08	43	27	129.36
P46821|MAP1B_HUMAN	209.92	26	26	1.02E+08	53	52	173.39
P60891|PRPS1_HUMAN	207.79	81	81	3.02E+08	36	14	171.67
P48668|K2C6C_HUMAN	207.31	54	54		38	0	159.36
Q9Y6Y0|NS1BP_HUMAN	207.18	70	70	7.41E+08	34	34	143.02
P62701|RS4X_HUMAN	207.13	70	70	2.66E+08	32	14	176.99
P02538|K2C6A_HUMAN	205.72	53	53	1.22E+07	38	2	158.49
P53621|COPA_HUMAN	205.25	41	41	1.33E+08	36	35	117.09
P62424|RL7A_HUMAN	205.03	60	60	8.26E+08	26	25	179.33
P48643|TCPE_HUMAN	204.72	70	70	2.58E+08	30	29	137.82
P06576|ATPB_HUMAN	204.66	80	80	3.39E+08	28	28	162.62
P10809|CH60_HUMAN	204.06	57	57	1.06E+08	26	26	188.61
P38646|GRP75_HUMAN	203.77	67	67	4.84E+08	36	35	113.08
P68104|EF1A1_HUMAN	203.72	78	78	2.95E+05	29	1	185.95
P11908|PRPS2_HUMAN	203.4	82	82	1.88E+08	33	16	152.02
Q9Y265|RUVB1_HUMAN	202.15	77	77	8.91E+08	38	38	160.87
P05388|RLA0_HUMAN	201.68	90	90	3.28E+08	35	18	182.59
Q6PEY2|TBA3E_HUMAN	201.67	56	56		26	0	163.97
Q05639|EF1A2_HUMAN	201.29	64	64	1.01E+08	28	10	187.96
P13647|K2C5_HUMAN	199.76	59	59	1.37E+08	39	24	201.83
P12236|ADT3_HUMAN	199.7	81	81	1.21E+08	29	7	127.21
Q13367|AP3B2_HUMAN	199.19	48	48	1.25E+08	42	35	104.49
P13010|XRCC5_HUMAN	198.47	69	69	1.67E+08	38	36	130.21
O43175|SERA_HUMAN	197.97	64	64	2.21E+08	27	26	114.18
O60841|IF2P_HUMAN	197.7	42	42	1.39E+08	36	34	187.37
P54652|HSP72_HUMAN	197.62	22	22	2.32E+07	20	3	127.35
Q00839|HNRPU_HUMAN	197.16	46	46	3.62E+08	46	44	148.23
Q5VTE0|EF1A3_HUMAN	196.98	62	62		26	0	185.95
P68133|ACTS_HUMAN	196.69	57	57		41	0	179.66
P39023|RL3_HUMAN	196.4	57	57	7.85E+08	31	30	136.53
Q00610|CLH1_HUMAN	195.62	28	28	7.12E+07	38	38	209.92
Q562R1|ACTBL_HUMAN	195.34	43	43	1.79E+07	22	3	160.42
O94832|MYO1D_HUMAN	195.09	41	41	1.03E+08	34	32	214.85
Q9NVI7|ATD3A_HUMAN	193.56	54	54	5.65E+07	32	12	167.93
Q02978|M2OM_HUMAN	193.22	64	64	2.61E+08	23	23	121.87
O75592|MYCB2_HUMAN	193.18	12	12	4.03E+07	41	40	178.69
P12956|XRCC6_HUMAN	192.79	55	55	1.28E+08	33	33	157.72
P23458|JAK1_HUMAN	192.66	46	46	1.75E+08	48	45	193.79
O43143|DHX15_HUMAN	192.55	39	39	1.44E+08	28	27	215.95
O95714|HERC2_HUMAN	192.33	15	15	4.75E+07	44	43	166.78
Q9UJS0|CMC2_HUMAN	190.63	63	63	2.69E+08	36	24	116.14
O75643|U520_HUMAN	190.45	32	32	1.00E+08	49	49	182.02
P49411|EFTU_HUMAN	190.13	68	68	5.08E+08	30	29	105.43
Q9UKL0|RCOR1_HUMAN	188.97	56	56	5.76E+08	30	25	0

P09874|PARP1_HUMAN	188.75	37	37	5.51E+07	27	27	107.75
Q9NY65|TBA8_HUMAN	188.32	38	38		24	0	0
P26640|SYVC_HUMAN	187.77	24	24	3.81E+07	19	19	144.46
Q92769|HDAC2_HUMAN	187.36	68	68	1.59E+08	27	15	0
P61160|ARP2_HUMAN	187.12	65	65	2.69E+08	24	22	160.33
Q9BUF5|TBB6_HUMAN	186.32	45	45	3.32E+07	27	1	149.7
Q15393|SF3B3_HUMAN	186.12	29	29	4.89E+07	23	23	196.52
P23396|RS3_HUMAN	185.55	74	74	6.70E+08	28	28	176.1
Q14204|DYHC1_HUMAN	184.89	17	17	1.08E+08	61	60	126.32
P12235|ADT1_HUMAN	184.52	55	55	2.37E+07	20	2	105.87
P02533|K1C14_HUMAN	183.73	61	61	2.69E+07	30	1	190.51
Q7L2E3|DHX30_HUMAN	183.28	32	32	7.67E+07	32	31	181.35
P22090|RS4Y1_HUMAN	183.07	53	53	2.38E+06	19	2	146.33
P33993|MCM7_HUMAN	182.96	45	45	1.31E+08	27	25	117.5
Q6UB35|C1TM_HUMAN	182.54	25	25	1.71E+07	16	16	40.96
Q16531|DDB1_HUMAN	182.31	33	33	9.33E+07	30	30	130.53
Q9P258|RCC2_HUMAN	182.26	61	61	1.10E+08	25	25	141.87
P15924|DESP_HUMAN	181.87	17	17	5.89E+07	36	34	173.95
Q04695|K1C17_HUMAN	179.46	61	61	2.95E+07	26	12	138.73
Q9GZR7|DDX24_HUMAN	178.14	33	33	4.09E+07	23	21	117.33
Q14839|CHD4_HUMAN	177.29	30	30	5.90E+07	37	24	144.33
Q6P2Q9|PRP8_HUMAN	177.03	13	13	3.44E+07	23	22	168.46
Q9BRS2|RIOK1_HUMAN	176.68	44	44	1.85E+08	21	21	161.46
O75746|CMC1_HUMAN	176.61	58	58	9.25E+07	34	22	117.27
P46781|RS9_HUMAN	176.25	53	53	4.91E+08	26	26	129.86
Q9UBW7|ZMYM2_HUMAN	175.49	35	35	1.48E+08	34	34	0
Q9BVQ7|SPA5L_HUMAN	174.94	40	40	3.94E+07	17	17	70.51
Q3ZCM7|TBB8_HUMAN	174.77	27	27		20	0	0
Q92673|SORL_HUMAN	174.14	20	20	1.42E+08	31	31	187.01
Q9BUA3|CK084_HUMAN	174.13	61	61	6.90E+07	16	16	169.29
Q8NB90|SPAT5_HUMAN	174.1	37	37	3.09E+07	20	18	81.85
Q08945|SSRP1_HUMAN	173.74	39	39	1.95E+08	25	23	139.29
Q13200|PSMD2_HUMAN	173.22	27	27	5.21E+07	18	17	112.66
Q6S8J3|POTEE_HUMAN	173.16	11	11		23	0	156.98
Q12905|ILF2_HUMAN	172.97	62	62	1.18E+08	16	16	140.95
P18124|RL7_HUMAN	172.93	63	63	9.70E+08	28	26	163.01
P11310|ACADM_HUMAN	172.89	36	36	6.82E+07	13	13	46.93
P30153|2AAA_HUMAN	171.91	49	49	1.18E+08	23	19	0
P61158|ARP3_HUMAN	171.45	60	60	2.42E+08	21	16	160.09
Q12906|ILF3_HUMAN	171.28	40	40	8.68E+07	27	24	114.69
P49368|TCPG_HUMAN	171.14	41	41	6.19E+07	19	17	133.88
A6NNZ2|TBB8L_HUMAN	170.6	26	26		19	0	141.12
P62753|RS6_HUMAN	170.09	47	47	3.34E+08	19	19	146.11
O00425|IF2B3_HUMAN	169.44	44	44	6.03E+07	21	18	178.97
Q8WWY3|PRP31_HUMAN	169.29	49	49	3.06E+08	22	22	154.28
Q92616|GCN1_HUMAN	169.19	14	14	1.64E+07	25	24	31.81
P51114|FXR1_HUMAN	169.17	34	34	4.65E+07	14	13	158.33
Q07065|CKAP4_HUMAN	168.8	43	43	4.91E+07	21	20	154.23
Q9Y383|LC7L2_HUMAN	168.77	49	49	1.49E+08	20	16	132.88
Q92598|HS105_HUMAN	168.06	36	36	6.57E+07	24	21	101.01

P22695|QCR2_HUMAN	167.99	47	47	1.49E+08	17	17	90.5
P46777|RL5_HUMAN	167.73	58	58	2.86E+08	21	21	149
Q9Y4I1|MYO5A_HUMAN	167.73	19	19	5.68E+07	30	29	177.05
Q02878|RL6_HUMAN	167.71	49	49	8.15E+08	23	23	156.61
Q9BUJ2|HNRL1_HUMAN	167.54	30	30	7.73E+07	19	17	181.19
P23588|IF4B_HUMAN	167.07	39	39	7.27E+08	20	20	127.43
P50416|CPT1A_HUMAN	165.22	32	32	9.68E+07	21	21	88.92
Q14195|DPYL3_HUMAN	165.15	48	48	7.77E+07	18	13	154.2
O75533|SF3B1_HUMAN	164.88	25	25	3.23E+07	23	21	212.51
Q15029|U5S1_HUMAN	164.44	33	33	5.59E+07	23	22	173
P21108|PRPS3_HUMAN	164.36	25	25		15	0	114.89
P45954|ACDSB_HUMAN	164.33	46	46	8.71E+07	14	14	40.39
A3KMH1|VWA8_HUMAN	163.68	21	21	3.12E+07	28	28	118.71
P26641|EF1G_HUMAN	163.58	36	36	1.98E+08	13	13	145.19
Q9Y2J2|E41L3_HUMAN	163.32	28	28	7.29E+07	25	25	141.5
P62917|RL8_HUMAN	163.21	51	51	5.30E+08	20	20	139.64
P07814|SYEP_HUMAN	163.14	23	23	2.89E+07	23	23	106.89
P04844|RPN2_HUMAN	162.88	36	36	4.98E+07	13	13	47.18
Q15057|ACAP2_HUMAN	162.75	43	43	1.21E+08	29	26	133.26
O15084|ANR28_HUMAN	162.42	16	16	2.83E+07	14	11	173.52
Q14152|EIF3A_HUMAN	162.15	25	25	5.99E+07	25	24	170.28
P98175|RBM10_HUMAN	162	33	33	2.16E+08	22	21	212.16
Q9UQ03|COR2B_HUMAN	161.88	42	42	1.24E+08	17	17	100.5
O95757|HS74L_HUMAN	161.67	28	28	4.36E+07	19	16	97.69
P25205|MCM3_HUMAN	161.6	30	30	4.71E+07	18	17	135.19
P40939|ECHA_HUMAN	161.39	38	38	7.18E+07	20	20	68.79
P08238|HS90B_HUMAN	161.36	35	35	6.40E+07	24	12	173.76
P42704|LPPRC_HUMAN	161.08	15	15	1.95E+07	20	18	136.97
O43390|HNRPR_HUMAN	160.89	38	38	5.13E+07	20	12	146.16
P40227|TCPZ_HUMAN	160.77	37	37	3.51E+07	16	15	188.45
P17812|PYRG1_HUMAN	160.42	31	31	3.03E+07	16	14	136.51
P62829|RL23_HUMAN	160.09	48	48	9.18E+07	10	10	109.28
P17987|TCPA_HUMAN	160.01	50	50	7.12E+07	19	18	154.41
P15880|RS2_HUMAN	159.65	40	40	3.32E+08	14	14	138.51
Q5T2N8|ATD3C_HUMAN	159.48	34	34		15	0	122.45
Q13310|PABP4_HUMAN	159.47	40	40	4.42E+07	19	10	191.49
P43246|MSH2_HUMAN	158.88	27	27	5.08E+07	21	21	98.26
P46459|NSF_HUMAN	158.87	41	41	3.46E+07	20	19	30.68
P27708|PYR1_HUMAN	158.44	15	15	2.46E+07	21	21	95.59
O95831|AIFM1_HUMAN	158.39	48	48	1.11E+08	21	21	35.28
Q07020|RL18_HUMAN	158.12	42	42	7.57E+08	13	13	122.52
Q14687|GSE1_HUMAN	158.03	26	26	1.60E+08	22	20	0
Q16891|MIC60_HUMAN	157.85	38	38	6.15E+07	20	20	72.47
P41091|IF2G_HUMAN	157.27	47	47	9.29E+07	16	16	154.16
P35580|MYH10_HUMAN	157.17	13	13	7.92E+06	21	12	163.49
Q9Y262|EIF3L_HUMAN	157.07	23	23	2.00E+07	13	13	129.89
P41250|SYG_HUMAN	157.05	33	33	5.12E+07	18	18	84.29
Q6PKG0|LARP1_HUMAN	156.81	19	19	1.95E+07	16	16	150.42
P06396|GELS_HUMAN	156.37	40	40	5.05E+07	18	18	71.6
P62333|PRS10_HUMAN	155.13	40	40	3.49E+07	12	12	0

P62195|PRS8_HUMAN	154.8	37	37	3.02E+07	10	9	0
Q8NHW5|RLA0L_HUMAN	154.48	39	39		16	0	151.35
P05198|IF2A_HUMAN	154.39	67	67	9.72E+07	18	18	147.21
P06748|NPM_HUMAN	154.32	53	53	3.44E+08	16	15	100.91
P78371|TCPB_HUMAN	154.28	43	43	4.58E+07	18	18	163.21
P32969|RL9_HUMAN	154.22	72	72	1.82E+08	14	14	127.46
Q15365|PCBP1_HUMAN	154.02	51	51	1.98E+07	12	8	56.22
P48735|IDHP_HUMAN	153.96	33	33	4.64E+07	14	13	91.51
Q9H2U1|DHX36_HUMAN	153.86	22	22	1.98E+07	14	11	175.74
Q9ULV0|MYO5B_HUMAN	153.74	20	20	5.68E+07	26	26	165.71
A5A3E0|POTEF_HUMAN	153.47	9	9	1.43E+06	20	1	143.05
Q5JUX0|SPIN3_HUMAN	153.42	55	55	3.41E+07	10	6	140.57
Q14498|RBM39_HUMAN	153.21	47	47	1.63E+08	19	18	106.14
Q7Z406|MYH14_HUMAN	152.86	20	20	2.80E+07	30	23	175.64
P61247|RS3A_HUMAN	152.84	56	56	2.97E+08	20	20	158.28
P11940|PABP1_HUMAN	152.79	31	31	3.41E+07	18	4	183.22
P42166|LAP2A_HUMAN	152.57	33	33	2.92E+07	17	17	0
Q9HCE1|MOV10_HUMAN	152.56	29	29	3.98E+07	26	25	148.55
P63151|2ABA_HUMAN	152.5	53	53	6.57E+07	17	11	115.07
Q8TD47|RS4Y2_HUMAN	151.49	41	41	1.06E+06	16	1	129
Q9H3U1|UN45A_HUMAN	151.39	12	12	8.14E+06	8	8	0
O15144|ARPC2_HUMAN	151.19	80	80	2.06E+08	21	21	163.67
Q16795|NDUA9_HUMAN	150.87	33	33	2.77E+07	11	11	0
Q13547|HDAC1_HUMAN	150.63	44	44	6.36E+06	17	5	0
Q12965|MYO1E_HUMAN	150.22	21	21	3.32E+07	20	15	96.86
P27635|RL10_HUMAN	150.11	65	65	1.52E+08	20	8	142.32
Q99714|HCD2_HUMAN	149.81	62	62	2.40E+07	10	10	83.28
Q9Y657|SPIN1_HUMAN	149.62	68	68	6.89E+07	11	7	116.57
Q06830|PRDX1_HUMAN	149.11	59	59	3.61E+07	12	9	112.06
P14868|SYDC_HUMAN	148.99	42	42	3.56E+07	20	20	127.89
O60506|HNRPQ_HUMAN	148.63	42	42	8.44E+07	24	16	164.23
P55209|NP1L1_HUMAN	148.57	43	43	6.32E+07	11	9	114.36
P26373|RL13_HUMAN	148.53	39	39	5.41E+08	15	15	117.3
P36542|ATPG_HUMAN	148.4	44	44	1.08E+08	12	12	120.35
Q9Y678|COPG1_HUMAN	148.32	23	23	1.97E+07	14	12	56.29
P61978|HNRPK_HUMAN	148.28	38	38	9.58E+07	16	16	186.24
Q9P219|DAPLE_HUMAN	147.89	19	19	4.14E+07	24	24	157.89
O95793|STAU1_HUMAN	147.86	23	23	4.51E+07	15	15	103.34
P30041|PRDX6_HUMAN	147.85	40	40	3.88E+07	10	10	148.24
O76021|RL1D1_HUMAN	147.41	31	31	2.37E+07	13	13	50.71
P46779|RL28_HUMAN	147.36	29	29	1.49E+08	11	11	75.3
P34932|HSP74_HUMAN	147.34	28	28	2.70E+07	19	18	0
Q9Y2H1|ST38L_HUMAN	146.74	36	36	2.57E+07	17	10	124.56
Q13363|CTBP1_HUMAN	146.7	53	53	2.21E+08	22	15	0
P31943|HNRH1_HUMAN	146.61	44	44	9.18E+07	18	9	158.57
P0CG38|POTEI_HUMAN	146.53	7	7		18	0	124.03
Q13435|SF3B2_HUMAN	145.75	26	26	4.42E+07	17	16	154.26
P34931|HS71L_HUMAN	145.67	20	20		11	0	104.6
P50990|TCPQ_HUMAN	145.16	36	36	3.03E+07	13	13	147.52
P62280|RS11_HUMAN	144.92	59	59	2.55E+08	15	15	121.25

Q9P1Y5|CAMP3_HUMAN	144.92	26	26	3.67E+07	17	16	162.25
Q7Z794|K2C1B_HUMAN	144.87	30	30	4.38E+07	17	9	102.58
Q9BYX7|ACTBM_HUMAN	144.78	20	20		16	0	0
Q14697|GANAB_HUMAN	144.64	20	20	2.78E+07	15	15	149.66
P61313|RL15_HUMAN	144.48	43	43	4.27E+08	11	11	119.85
P62249|RS16_HUMAN	144.3	51	51	2.76E+08	11	11	132.79
P62241|RS8_HUMAN	144.25	61	61	4.22E+08	16	16	146.29
P26599|PTBP1_HUMAN	144.05	45	45	3.02E+07	11	10	140.66
P62191|PRS4_HUMAN	143.96	38	38	3.58E+07	12	11	107.67
Q9BQG0|MBB1A_HUMAN	143.91	18	18	2.44E+07	19	19	50.46
P04843|RPN1_HUMAN	143.18	27	27	5.57E+07	11	11	115.2
P18621|RL17_HUMAN	142.94	51	51	3.00E+08	14	14	136.26
tr|A0A0A6YYL6|A0A0A6YYL6	142.94	41	41	3.00E+08	14	14	136.26
Q02543|RL18A_HUMAN	142.87	52	52	2.58E+08	17	17	116.2
O00303|EIF3F_HUMAN	142.79	34	34	2.83E+07	8	8	126.13
P62906|RL10A_HUMAN	142.76	58	58	3.01E+08	15	15	131.15
P46778|RL21_HUMAN	142.42	41	41	1.44E+08	14	13	109.95
Q9NUL3|STAU2_HUMAN	142.42	32	32	2.81E+07	14	14	120.65
P16989|YBOX3_HUMAN	142.19	44	44	1.84E+07	12	7	78.31
P17066|HSP76_HUMAN	142.04	16	16		10	0	93.85
P50991|TCPD_HUMAN	141.85	39	39	2.88E+07	14	13	152.32
P52701|MSH6_HUMAN	141.84	18	18	3.05E+07	20	18	0
P11387|TOP1_HUMAN	141.5	25	25	5.81E+07	19	14	138.52
Q9UQ35|SRRM2_HUMAN	140.73	10	10	3.87E+07	18	18	197.6
Q15750|TAB1_HUMAN	140.6	41	41	4.87E+07	13	13	154.82
Q9UKN8|TF3C4_HUMAN	140.6	29	29	1.79E+07	14	14	0
P14625|ENPL_HUMAN	140.39	28	28	5.56E+07	17	16	140.39
P62081|RS7_HUMAN	140.05	65	65	2.39E+08	11	11	96.92
P55072|TERA_HUMAN	139.99	29	29	2.54E+07	18	17	142.45
P62277|RS13_HUMAN	139.9	58	58	3.08E+08	17	16	152.22
Q06210|GFPT1_HUMAN	139.83	25	25	1.59E+07	13	13	120.91
P51532|SMCA4_HUMAN	139.68	13	13	3.45E+06	15	9	88.95
O75531|BAF_HUMAN	139.66	70	70	7.66E+07	8	8	0
P35998|PRS7_HUMAN	139.56	48	48	4.16E+07	19	18	87.37
Q5T9A4|ATD3B_HUMAN	139.55	27	27	1.75E+07	16	2	146.59
P38606|VATA_HUMAN	139.25	39	39	2.68E+07	13	13	39.81
P28331|NDUS1_HUMAN	139	23	23	1.69E+07	12	12	66.95
P06493|CDK1_HUMAN	138.72	55	55	3.80E+07	15	13	74.69
P07900|HS90A_HUMAN	138.72	34	34	1.29E+07	16	8	155.03
P62244|RS15A_HUMAN	138.33	64	64	2.48E+08	13	13	134.84
P08195|4F2_HUMAN	138.32	27	27	4.32E+07	11	11	47.17
P62269|RS18_HUMAN	138.27	47	47	2.48E+08	12	11	128.49
P62913|RL11_HUMAN	137.82	50	50	3.00E+08	9	9	98.16
Q13263|TIF1B_HUMAN	137.39	30	30	6.32E+07	17	17	139.51
P23258|TBG1_HUMAN	137.31	47	47	2.91E+07	13	13	0
Q13561|DCTN2_HUMAN	137.13	32	32	2.53E+07	12	12	71.27
P62750|RL23A_HUMAN	136.76	45	45	2.04E+08	10	10	135.93
Q00325|MPCP_HUMAN	136.75	39	39	1.90E+08	17	16	70.82
O15143|ARC1B_HUMAN	136.68	40	40	6.54E+07	13	12	115.74
O95678|K2C75_HUMAN	136.4	18	18	0.00E+00	14	1	116.99

P12004|PCNA_HUMAN	136.36	57	57	1.20E+08	12	12	83.67
P62910|RL32_HUMAN	136.2	41	41	1.28E+08	11	11	0
Q92747|ARC1A_HUMAN	136.2	53	53	8.64E+07	14	13	121.91
P67809|YBOX1_HUMAN	136.04	48	48	1.13E+08	13	8	105.35
P55084|ECHB_HUMAN	135.72	43	43	4.73E+07	15	15	0
P55265|DSRAD_HUMAN	135.71	15	15	1.68E+07	15	15	91.06
Q96GQ7|DDX27_HUMAN	135.33	22	22	4.11E+07	16	16	101.41
P29144|TPP2_HUMAN	135.18	15	15	1.48E+07	11	11	113.98
P12035|K2C3_HUMAN	135.06	8	8		10	0	119.74
P52597|HNRPF_HUMAN	135.03	39	39	5.30E+07	11	8	119.55
tr|B4DLN1|B4DLN1_HUMAN	134.83	22	22	4.86E+07	10	10	0
P14923|PLAK_HUMAN	134.59	21	21	1.56E+07	10	9	155.51
P33992|MCM5_HUMAN	134.29	25	25	2.19E+07	15	15	121.58
O00567|NOP56_HUMAN	134.28	35	35	2.92E+07	15	14	90.62
Q8TAQ2|SMRC2_HUMAN	134.12	9	9	5.99E+06	9	6	25.95
Q92499|DDX1_HUMAN	133.98	24	24	3.46E+07	13	13	120.92
P27348|1433T_HUMAN	133.97	59	59	5.35E+07	11	9	0
Q06787|FMR1_HUMAN	133.8	31	31	1.63E+07	13	11	113.97
P05787|K2C8_HUMAN	133.69	23	23	2.01E+07	14	6	115.73
Q9Y3I0|RTCB_HUMAN	133.67	33	33	2.97E+07	10	10	106.21
P17980|PRS6A_HUMAN	133.63	38	38	3.24E+07	11	11	102.63
Q9NR30|DDX21_HUMAN	133.48	18	18	1.73E+07	13	9	65.5
Q01546|K22O_HUMAN	133.12	12	12	1.27E+07	13	2	121.37
O94906|PRP6_HUMAN	132.45	12	12	1.86E+07	9	9	81.01
O14980|XPO1_HUMAN	132.41	10	10	1.16E+07	8	7	69.64
Q99575|POP1_HUMAN	132.23	20	20	2.21E+07	12	12	49.4
P07237|PDIA1_HUMAN	131.86	23	23	1.88E+07	11	11	145.93
P54105|ICLN_HUMAN	131.68	62	62	4.05E+08	7	7	123.8
P31946|1433B_HUMAN	131.57	52	52	1.19E+07	9	4	0
Q6P3W7|SCYL2_HUMAN	131.54	25	25	4.38E+07	17	17	151.22
Q9H845|ACAD9_HUMAN	131.05	25	25	1.48E+07	9	9	0
Q12789|TF3C1_HUMAN	130.78	11	11	1.93E+07	15	14	100.09
P46782|RS5_HUMAN	130.71	49	49	5.49E+07	9	9	80.76
P0CG39|POTEJ_HUMAN	130.58	6	6		11	0	0
P07910|HNRPC_HUMAN	130.45	23	23	4.66E+07	7	4	152.36
O75976|CBPD_HUMAN	129.87	15	15	1.19E+07	13	13	106.57
P05387|RLA2_HUMAN	129.86	98	98	1.87E+08	10	9	123.98
P08708|RS17_HUMAN	129.73	55	55	8.33E+07	7	7	104.35
Q96AQ6|PBIP1_HUMAN	129.64	27	27	5.10E+07	14	14	0
P63104|1433Z_HUMAN	129.55	64	64	5.18E+07	14	10	30.31
Q9H3G5|CPVL_HUMAN	129.46	37	37	6.85E+07	9	9	88.39
Q5XKE5|K2C79_HUMAN	129.41	11	11	1.58E+06	10	1	123.38
Q8IZL8|PELP1_HUMAN	129.1	12	12	1.63E+07	8	8	0
A2RRP1|NBAS_HUMAN	128.84	7	7	5.51E+06	12	11	42.38
P22087|FBRL_HUMAN	128.8	45	45	5.94E+07	11	11	129.9
Q8TDI0|CHD5_HUMAN	128.53	8	8		12	0	111.48
P62263|RS14_HUMAN	128.47	43	43	1.67E+08	8	8	122.47
P52272|HNRPM_HUMAN	128.44	27	27	3.70E+07	14	14	166.58
Q9NVP1|DDX18_HUMAN	128.39	17	17	1.46E+07	9	9	47.55
P40429|RL13A_HUMAN	128.27	44	44	3.77E+08	18	18	126.65

P56192|SYMC_HUMAN	128.18	20	20	3.71E+07	15	15	0
Q9NXF1|TEX10_HUMAN	127.85	16	16	1.74E+07	12	10	46.97
P05023|AT1A1_HUMAN	127.51	17	17	1.08E+07	14	6	43.43
P63173|RL38_HUMAN	127.27	57	57	6.78E+07	6	6	85.79
P48444|COPD_HUMAN	127.21	32	32	2.43E+07	12	12	69.69
P48047|ATPO_HUMAN	127.07	52	52	3.49E+07	9	9	104.8
O75688|PPM1B_HUMAN	127.06	26	26	4.70E+07	10	9	151.44
Q9H4B7|TBB1_HUMAN	126.87	9	9		8	0	105.03
P11388|TOP2A_HUMAN	126.78	12	12	1.41E+07	13	9	112.27
Q15020|SART3_HUMAN	126.67	19	19	1.84E+07	12	11	96.02
P07339|CATD_HUMAN	126.52	34	34	5.48E+05	12	1	115.7
Q9H9B4|SFXN1_HUMAN	126.37	41	41	1.27E+07	9	6	0
Q9Y4P3|TBL2_HUMAN	126.28	23	23	1.28E+07	7	7	55.04
Q9Y2T7|YBOX2_HUMAN	126.04	35	35	3.89E+06	9	7	68.5
Q66LE6|2ABD_HUMAN	125.56	31	31	1.81E+06	10	4	0
O15020|SPTN2_HUMAN	125.49	7	7	3.39E+07	18	2	112.69
P13639|EF2_HUMAN	125.48	21	21	1.89E+07	12	11	83.52
P56545|CTBP2_HUMAN	125.26	23	23	1.94E+07	10	3	0
P62841|RS15_HUMAN	125.14	43	43	1.16E+08	4	4	0
O75131|CPNE3_HUMAN	124.89	20	20	1.60E+07	8	8	117.98
P16615|AT2A2_HUMAN	124.25	23	23	3.75E+07	18	13	0
P53618|COPB_HUMAN	124.03	24	24	2.92E+07	15	14	74.05
P83731|RL24_HUMAN	123.98	42	42	7.24E+07	8	8	116.14
P30837|AL1B1_HUMAN	123.97	29	29	8.32E+06	10	9	0
Q9Y4E8|UBP15_HUMAN	123.88	7	7	4.32E+06	7	7	87.13
Q9NYL9|TMOD3_HUMAN	123.72	36	36	1.98E+07	9	9	77.28
Q92922|SMRC1_HUMAN	123.68	10	10	1.65E+07	9	6	81.76
Q92841|DDX17_HUMAN	123.6	15	15	4.48E+06	10	6	177.78
Q15366|PCBP2_HUMAN	123.44	30	30	1.94E+06	8	3	56.23
P35606|COPB2_HUMAN	123.42	21	21	2.29E+07	14	14	0
Q99832|TCPH_HUMAN	123.27	27	27	1.53E+07	11	10	153.39
P49916|DNLI3_HUMAN	122.96	17	17	1.71E+07	12	12	0
P35268|RL22_HUMAN	122.72	62	62	8.77E+07	8	7	88.27
P55884|EIF3B_HUMAN	122.42	34	34	5.56E+07	16	16	136.32
Q08J23|NSUN2_HUMAN	122.05	23	23	2.69E+07	11	11	56.55
Q9Y4F1|FARP1_HUMAN	122.03	19	19	1.27E+07	11	11	90.36
Q8IYB3|SRRM1_HUMAN	121.95	19	19	3.40E+07	13	13	137.16
Q8N1N4|K2C78_HUMAN	121.86	18	18	6.94E+06	12	6	125.68
tr|A0A1B0GU03|A0A1B0GU(	121.44	27	27	0.00E+00	12	1	115.7
Q96HS1|PGAM5_HUMAN	121.04	65	65	1.52E+08	15	14	133.32
Q99460|PSMD1_HUMAN	120.63	20	20	2.21E+07	12	12	112.67
Q96L21|RL10L_HUMAN	120.32	51	51	1.69E+06	13	1	0
Q9BUQ8|DDX23_HUMAN	119.84	17	17	2.09E+07	12	12	65.29
Q16875|F263_HUMAN	119.63	25	25	8.80E+06	9	7	113.6
P50914|RL14_HUMAN	119.42	37	37	4.14E+08	12	11	116.1
P05455|LA_HUMAN	119.14	26	26	1.65E+07	11	11	78.17
P55795|HNRH2_HUMAN	119.01	32	32	9.24E+06	12	4	138.04
O00203|AP3B1_HUMAN	118.94	6	6		7	0	0
Q99613|EIF3C_HUMAN	118.92	13	13	3.11E+07	11	11	131.57
P38919|IF4A3_HUMAN	118.8	31	31	1.50E+07	10	8	123.64

P62847|RS24_HUMAN	118.71	32	32	1.98E+08	7	7	104.28
Q96BD5|PF21A_HUMAN	118.25	17	17	1.86E+07	8	8	0
P61981|1433G_HUMAN	118.06	55	55	3.28E+07	9	6	0
Q8N3C0|ASCC3_HUMAN	117.94	5	5	1.00E+07	8	8	119.67
P46776|RL27A_HUMAN	117.92	40	40	2.60E+08	10	10	86.81
P30050|RL12_HUMAN	117.86	45	45	2.28E+08	7	7	110.45
Q9Y4L1|HYOU1_HUMAN	117.52	23	23	8.21E+07	18	18	141.86
Q9UKA4|AKA11_HUMAN	117.37	10	10	8.58E+06	10	10	0
Q02880|TOP2B_HUMAN	117.36	10	10	6.72E+06	11	8	126.54
Q14194|DPYL1_HUMAN	117.23	18	18	4.39E+06	7	4	81.69
Q14974|IMB1_HUMAN	117.2	12	12	2.26E+07	8	8	66.55
P16104|H2AX_HUMAN	117.15	52	52		6	0	130.48
P59998|ARPC4_HUMAN	117.06	61	61	7.94E+07	9	4	111.91
O75489|NDUS3_HUMAN	117	44	44	2.21E+07	11	9	75.45
Q14681|KCTD2_HUMAN	116.71	35	35	4.70E+07	6	5	111.13
P39019|RS19_HUMAN	116.62	53	53	1.08E+08	11	11	112.94
P22102|PUR2_HUMAN	116.54	13	13	3.17E+07	9	9	34.54
O00148|DX39A_HUMAN	116.52	12	12	7.26E+05	6	1	0
Q53GQ0|DHB12_HUMAN	116.29	25	25	1.94E+07	6	6	0
P16403|H12_HUMAN	116.2	23	23		6	0	105.18
Q9BRJ6|CG050_HUMAN	115.9	43	43	8.91E+06	8	8	0
Q9UMS4|PRP19_HUMAN	115.89	48	48	4.25E+07	14	13	127.15
O00178|GTPB1_HUMAN	115.85	24	24	1.11E+07	8	8	88.65
P26368|U2AF2_HUMAN	115.57	39	39	4.89E+07	9	9	121.35
Q3KQU3|MA7D1_HUMAN	115.4	12	12	9.61E+06	6	6	0
Q9BZE4|NOG1_HUMAN	115.4	25	25	1.24E+07	10	10	63.49
P11586|C1TC_HUMAN	114.8	15	15	1.64E+07	11	11	92.01
P78362|SRPK2_HUMAN	114.34	25	25	2.49E+07	10	8	52.67
Q16777|H2A2C_HUMAN	114.17	58	58	1.08E+07	6	1	143.67
Q6FI13|H2A2A_HUMAN	114.17	58	58	1.08E+07	6	1	143.67
O00231|PSD11_HUMAN	113.86	26	26	1.57E+07	9	9	98.54
O95782|AP2A1_HUMAN	113.51	14	14	3.83E+06	9	6	85.92
O43242|PSMD3_HUMAN	113.5	29	29	1.97E+07	10	10	77.06
Q92900|RENT1_HUMAN	113.19	15	15	2.30E+07	15	14	184.36
P16402|H13_HUMAN	113.13	17	17		5	0	105.18
P62316|SMD2_HUMAN	113.12	57	57	4.30E+07	7	7	96.82
P13637|AT1A3_HUMAN	113.1	12	12		9	0	0
Q99623|PHB2_HUMAN	112.89	28	28	1.25E+07	6	6	99.51
Q3ZCQ8|TIM50_HUMAN	112.86	23	23	2.14E+07	7	7	0
P04908|H2A1B_HUMAN	112.7	58	58	2.96E+06	7	2	124.41
Q7L7L0|H2A3_HUMAN	112.7	58	58	2.96E+06	7	2	124.41
Q93077|H2A1C_HUMAN	112.7	58	58	2.96E+06	7	2	124.41
A6NHL2|TBAL3_HUMAN	112.66	11	11	3.06E+05	7	1	68.18
Q9NP66|HM20A_HUMAN	112.57	25	25	3.93E+07	7	7	0
P17858|PFKAL_HUMAN	112.55	19	19	1.01E+07	10	7	0
P37108|SRP14_HUMAN	112.33	41	41	7.37E+07	7	7	89.39
P84098|RL19_HUMAN	112.23	32	32	1.23E+08	10	10	68.12
Q14203|DCTN1_HUMAN	112.23	12	12	7.74E+06	10	9	73.32
Q9P2K3|RCOR3_HUMAN	111.72	15	15	1.12E+06	7	2	0
P62318|SMD3_HUMAN	111.6	51	51	7.29E+07	6	6	66.2

O60264|SMCA5_HUMAN	111.52	14	14	7.06E+06	13	8	0
P0C0S8|H2A1_HUMAN	111.26	58	58		6	0	139.63
P20671|H2A1D_HUMAN	111.26	58	58		6	0	139.63
Q96KK5|H2A1H_HUMAN	111.26	59	59		6	0	139.63
Q99878|H2A1J_HUMAN	111.26	59	59		6	0	139.63
Q9BTM1|H2AJ_HUMAN	111.26	58	58		6	0	139.63
tr|A0A0U1RRH7|A0A0U1RRH	111.26	44	44		6	0	139.63
Q96NB2|SFXN2_HUMAN	111.2	21	21	8.81E+06	6	6	0
Q7Z2T5|TRM1L_HUMAN	111.14	21	21	1.71E+07	8	8	0
P62851|RS25_HUMAN	111.1	35	35	1.84E+08	8	8	103.74
Q9BWM7|SFXN3_HUMAN	111	26	26	7.04E+06	7	5	0
Q8IUE6|H2A2B_HUMAN	110.98	52	52	2.23E+06	5	1	128.53
P35249|RFC4_HUMAN	110.93	23	23	9.33E+06	7	7	57.72
P62888|RL30_HUMAN	110.93	70	70	1.00E+08	9	8	118.98
Q9Y2W1|TR150_HUMAN	110.85	18	18	3.36E+07	13	13	135.89
Q8TDN6|BRX1_HUMAN	110.67	40	40	4.29E+07	9	8	0
P57772|SELB_HUMAN	110.41	14	14	4.00E+06	5	5	0
P19012|K1C15_HUMAN	110.39	11	11		8	0	95.79
P32119|PRDX2_HUMAN	110.27	47	47	1.75E+07	10	9	64.34
Q8WXI9|P66B_HUMAN	110.16	23	23	5.53E+06	7	5	62.49
Q9Y224|CN166_HUMAN	109.74	38	38	7.90E+06	6	6	116.95
P14618|KPYM_HUMAN	109.65	23	23	9.31E+06	6	6	160.11
Q96SB4|SRPK1_HUMAN	109.52	14	14	1.16E+07	6	4	81.71
P17480|UBF1_HUMAN	109.31	16	16	2.35E+07	10	10	35.18
P61353|RL27_HUMAN	109.03	52	52	2.89E+08	11	11	85.13
P62805|H4_HUMAN	108.99	55	55	3.16E+07	6	6	118.67
P49327|FAS_HUMAN	108.93	6	6	8.07E+06	9	9	91.64
O60524|NEMF_HUMAN	108.9	11	11	1.32E+07	10	10	106.74
Q9P2R7|SUCB1_HUMAN	108.82	20	20	1.88E+07	9	9	0
O75955|FLOT1_HUMAN	108.78	26	26	7.66E+06	8	8	130.76
P61254|RL26_HUMAN	108.68	46	46	1.72E+08	9	9	75.1
Q9UHB6|LIMA1_HUMAN	108.56	12	12	9.22E+06	7	7	0
O75569|PRKRA_HUMAN	108.33	45	45	3.00E+07	10	9	108.45
P43243|MATR3_HUMAN	108.25	24	24	7.36E+07	13	13	156.05
tr|G3V3G9|G3V3G9_HUMAN	108.21	15	15	1.28E+07	9	9	0
O15294|OGT1_HUMAN	108.13	10	10	2.12E+07	8	8	101.72
P50402|EMD_HUMAN	108.07	35	35	4.19E+07	8	8	0
Q6KB66|K2C80_HUMAN	108.02	21	21	3.45E+06	9	7	55.21
O43318|M3K7_HUMAN	108.01	14	14	8.74E+06	7	7	118.16
P55036|PSMD4_HUMAN	107.99	25	25	1.08E+07	7	7	0
O60762|DPM1_HUMAN	107.84	51	51	1.92E+07	9	8	30.85
Q13838|DX39B_HUMAN	107.69	10	10		5	0	72.32
Q9UHX1|PUF60_HUMAN	107.62	37	37	4.63E+07	12	12	133.27
Q02413|DSG1_HUMAN	107.55	13	13	1.18E+07	9	9	78.53
P62266|RS23_HUMAN	107.54	36	36	1.05E+08	7	7	102.75
O14828|SCAM3_HUMAN	107.45	22	22	1.43E+07	5	5	58.63
Q13823|NOG2_HUMAN	107.38	16	16	1.33E+07	7	7	91.36
O14579|COPE_HUMAN	107.28	49	49	2.12E+07	8	8	0
P61163|ACTZ_HUMAN	107.14	41	41	2.11E+07	7	3	89.76
P78316|NOP14_HUMAN	106.91	7	7	1.02E+07	7	7	86.65

Q01813|PFKAP_HUMAN	106.83	16	16	9.09E+06	9	6	70.94
Q9H1A4|APC1_HUMAN	106.57	8	8	1.81E+07	12	12	24.1
Q7Z6Z7|HUWE1_HUMAN	106.48	4	4	5.15E+06	12	11	54.78
P09543|CN37_HUMAN	106.37	28	28	9.46E+06	10	9	0
Q14160|SCRIB_HUMAN	106.2	6	6	6.28E+06	8	6	0
Q9UJA5|TRM6_HUMAN	106.13	14	14	6.20E+06	5	5	0
Q96H55|MYO19_HUMAN	106.11	6	6	2.23E+06	4	4	0
P04062|GLCM_HUMAN	105.72	24	24	3.69E+07	9	9	49.4
P05783|K1C18_HUMAN	105.29	20	20	5.96E+06	6	5	61.09
P30876|RPB2_HUMAN	105.23	8	8	5.83E+06	8	8	93.76
Q13574|DGKZ_HUMAN	105.13	13	13	1.46E+07	8	7	86.62
Q07955|SRSF1_HUMAN	105.07	41	41	7.11E+07	9	8	143.38
Q86WJ1|CHD1L_HUMAN	105.03	12	12	4.42E+06	10	10	22.06
Q16629|SRSF7_HUMAN	104.57	27	27	2.67E+07	8	7	95.39
O95347|SMC2_HUMAN	104.4	11	11	1.37E+07	8	8	0
Q1KMD3|HNRL2_HUMAN	104.18	33	33	1.86E+07	15	14	146.1
tr|H3BQZ7|H3BQZ7_HUMAN	104.18	33	33	1.86E+07	15	14	146.1
P31689|DNJA1_HUMAN	104.14	34	34	3.09E+07	9	7	33.56
Q9UI10|EI2BD_HUMAN	104.09	19	19	8.38E+06	6	6	0
P60866|RS20_HUMAN	104.01	24	24	7.40E+07	4	4	100.09
Q2NL82|TSR1_HUMAN	103.97	24	24	1.75E+07	12	12	96.59
P11277|SPTB1_HUMAN	103.91	4	4		11	0	119.29
O00442|RTCA_HUMAN	103.64	24	24	1.09E+07	7	7	37.6
Q9Y2X3|NOP58_HUMAN	103.45	13	13	7.84E+06	5	5	103.44
Q92522|H1X_HUMAN	103.42	20	20	2.38E+07	6	6	69.9
O00268|TAF4_HUMAN	102.73	15	15	4.78E+06	9	7	27.2
Q9BZX2|UCK2_HUMAN	102.16	25	25	1.04E+07	4	4	0
Q9Y3U8|RL36_HUMAN	102.14	42	42	1.55E+08	9	9	113.46
P08727|K1C19_HUMAN	102.03	14	14	4.14E+06	8	1	85.27
Q09028|RBBP4_HUMAN	102.02	41	41	2.94E+07	9	4	115.41
P08729|K2C7_HUMAN	101.92	6	6		7	0	101.21
P62826|RAN_HUMAN	101.92	26	26	1.70E+07	5	5	50.28
Q9H0U4|RAB1B_HUMAN	101.85	43	43	2.39E+06	6	3	0
P39656|OST48_HUMAN	101.83	33	33	4.02E+07	9	9	57.67
Q9NQX4|MYO5C_HUMAN	101.69	10	10	4.63E+06	10	8	78.33
Q9P0J0|NDUAD_HUMAN	101.69	44	44	2.66E+07	5	5	0
Q14318|FKBP8_HUMAN	101.68	14	14	6.90E+06	5	5	0
Q9NW13|RBM28_HUMAN	101.63	13	13	8.32E+06	8	8	91.35
Q9BQ39|DDX50_HUMAN	101.38	11	11	2.75E+06	7	3	0
Q9NYU2|UGGG1_HUMAN	101.33	6	6	5.98E+06	9	8	69.8
Q96ST3|SIN3A_HUMAN	101.13	11	11	7.10E+06	11	11	0
Q9H361|PABP3_HUMAN	100.99	17	17	1.32E+06	10	1	137.36
Q8NE71|ABCF1_HUMAN	100.62	16	16	1.90E+07	11	11	88.42
Q8TDY2|RBCC1_HUMAN	100.46	8	8	1.01E+07	8	8	103.1
P50993|AT1A2_HUMAN	100.44	9	9		7	0	0
P30154|2AAB_HUMAN	100.37	5	5		4	0	0
O60832|DKC1_HUMAN	100.3	30	30	2.90E+07	11	11	80.3
P19013|K2C4_HUMAN	100.19	9	9	3.45E+06	6	1	100.37
P51570|GALK1_HUMAN	100.13	21	21	4.97E+06	6	6	0
P43686|PRS6B_HUMAN	99.71	34	34	2.19E+07	9	8	72.56

O15355|PPM1G_HUMAN	99.59	25	25	2.80E+07	7	7	0
Q9Y4B6|VPRBP_HUMAN	99.07	6	6	5.12E+06	6	5	0
P53999|TCP4_HUMAN	98.83	51	51	6.05E+07	9	8	75.93
Q9BVI4|NOC4L_HUMAN	98.77	28	28	1.28E+07	9	9	87.64
Q92947|GCDH_HUMAN	98.52	14	14	1.35E+07	4	4	0
Q13347|EIF3I_HUMAN	98.17	28	28	1.85E+07	6	6	121.64
P53985|MOT1_HUMAN	98.14	11	11	4.11E+07	6	6	0
Q9UNF1|MAGD2_HUMAN	98.11	18	18	1.00E+07	9	8	0
P62714|PP2AB_HUMAN	98.1	35	35	1.70E+07	6	6	30.35
P67775|PP2AA_HUMAN	98.1	35	35	1.70E+07	6	6	30.35
O75964|ATP5L_HUMAN	97.83	48	48	3.18E+07	4	4	0
Q9UBF2|COPG2_HUMAN	97.79	15	15	2.51E+06	8	6	0
O14880|MGST3_HUMAN	97.5	53	53	1.08E+07	4	4	54.76
O43837|IDH3B_HUMAN	97.43	10	10	2.95E+06	4	4	0
P04083|ANXA1_HUMAN	97.27	27	27	1.07E+07	6	6	72.62
P04406|G3P_HUMAN	97.13	55	55	4.74E+07	10	10	130.71
O75179|ANR17_HUMAN	97.07	3	3	1.30E+06	6	3	21.87
Q9NQ29|LUC7L_HUMAN	97.03	15	15	5.21E+05	5	1	0
Q00577|PURA_HUMAN	96.97	43	43	5.76E+06	9	5	104.52
O00410|IPO5_HUMAN	96.95	13	13	1.07E+07	10	10	84.77
P62899|RL31_HUMAN	96.4	41	41	2.60E+08	7	7	103.96
Q9H936|GHC1_HUMAN	96.12	42	42	2.28E+07	10	6	0
P42285|SK2L2_HUMAN	96.06	10	10	8.75E+06	9	9	105.2
Q01130|SRSF2_HUMAN	96.02	17	17	7.26E+07	4	2	109.18
Q9UKV3|ACINU_HUMAN	96	7	7	1.64E+07	6	6	96.62
Q9UHL4|DPP2_HUMAN	95.73	11	11	4.31E+06	4	4	0
Q9H4L4|SENP3_HUMAN	95.7	16	16	4.47E+06	5	5	0
P17844|DDX5_HUMAN	95.64	7	7	1.37E+05	5	1	151.18
P42766|RL35_HUMAN	95.55	31	31	1.57E+08	7	6	95.25
Q9Y4W6|AFG32_HUMAN	95.53	12	12	4.89E+06	8	8	0
Q7RTS7|K2C74_HUMAN	95.5	12	12	3.93E+04	11	1	90.16
Q12873|CHD3_HUMAN	95.25	6	6	1.28E+06	10	3	104.99
Q14240|IF4A2_HUMAN	95.22	15	15	8.01E+05	5	1	67.22
tr|A0A0A6YYG9|A0A0A6YYG	95.17	8	8		5	0	103.47
Q99733|NP1L4_HUMAN	95.14	25	25	2.23E+07	8	6	52.13
Q9H2V7|SPNS1_HUMAN	95.03	16	16	1.05E+07	4	4	0
O00483|NDUA4_HUMAN	94.89	68	68	1.22E+07	4	4	0
P46783|RS10_HUMAN	94.81	24	24	9.83E+06	5	2	107.54
O15371|EIF3D_HUMAN	94.42	18	18	1.73E+07	7	6	106.42
Q9BRT8|CBWD1_HUMAN	94.39	21	21	1.28E+06	6	1	0
Q12756|KIF1A_HUMAN	94.17	8	8	7.98E+06	9	8	44.83
Q96A33|CCD47_HUMAN	93.82	24	24	1.82E+07	8	8	0
Q00005|2ABB_HUMAN	93.66	11	11		4	0	0
Q9H6T3|RPAP3_HUMAN	93.62	10	10	2.56E+06	5	5	66.6
P18887|XRCC1_HUMAN	93.49	10	10	7.59E+06	5	5	0
P41252|SYIC_HUMAN	93.45	8	8	1.26E+07	8	7	93.83
Q86VP6|CAND1_HUMAN	93.44	11	11	7.30E+06	10	9	45.36
Q9NVE7|PANK4_HUMAN	93.36	9	9	4.94E+06	4	4	0
Q93008|USP9X_HUMAN	93.31	3	3	2.01E+06	7	3	127.08
O15027|SC16A_HUMAN	93.3	7	7	1.03E+07	9	9	29.11

Q5XKP0|MIC13_HUMAN	93.26	74	74	8.96E+06	4	4	0
P05386|RLA1_HUMAN	92.91	71	71	1.96E+08	5	4	63.04
Q12874|SF3A3_HUMAN	92.82	21	21	8.91E+06	6	6	103.11
Q9UKI9|PO2F3_HUMAN	92.67	24	24	2.00E+05	8	2	52.15
Q15084|PDIA6_HUMAN	92.64	22	22	4.87E+06	4	4	0
Q9GZS3|WDR61_HUMAN	92.6	17	17	2.45E+06	3	3	0
P60228|EIF3E_HUMAN	92.52	18	18	1.45E+07	7	7	152.63
Q9UBC5|MYO1A_HUMAN	92.5	7	7	1.32E+05	6	2	90.79
Q14247|SRC8_HUMAN	92.35	24	24	2.08E+07	10	10	117.9
Q9UNM6|PSD13_HUMAN	92.21	30	30	1.03E+07	9	8	40.24
E9PAV3|NACAM_HUMAN	92.16	6	6	1.41E+07	8	8	57.82
O96019|ACL6A_HUMAN	92.1	16	16	7.69E+06	5	4	0
Q86Y46|K2C73_HUMAN	92.05	7	7		9	0	88.16
P60842|IF4A1_HUMAN	91.89	12	12		4	0	62.11
Q9P1U1|ARP3B_HUMAN	91.72	10	10		5	0	94.16
P51116|FXR2_HUMAN	91.61	15	15	8.87E+06	6	5	99.56
Q92614|MY18A_HUMAN	91.6	4	4	4.34E+06	6	5	0
Q9Y2W6|TDRKH_HUMAN	91.56	8	8	9.00E+06	3	3	0
O75306|NDUS2_HUMAN	91.53	32	32	1.56E+07	10	10	0
P13646|K1C13_HUMAN	91.53	9	9		5	0	75.39
O14646|CHD1_HUMAN	91.39	6	6	4.41E+06	8	5	77.66
Q96QR8|PURB_HUMAN	91.33	32	32	5.49E+06	7	3	56.41
P57721|PCBP3_HUMAN	91.3	16	16		5	0	45.76
P35749|MYH11_HUMAN	91.03	3	3	1.42E+06	7	2	77.69
P16401|H15_HUMAN	90.98	15	15	1.07E+07	5	4	48.19
Q16769|QPCT_HUMAN	90.91	37	37	2.31E+07	8	8	0
P62314|SMD1_HUMAN	90.8	37	37	7.04E+07	3	3	114.76
Q9NXS2|QPCTL_HUMAN	90.68	25	25	8.63E+06	6	6	0
O00165|HAX1_HUMAN	90.66	33	33	2.19E+07	6	6	0
Q96JN8|NEUL4_HUMAN	90.58	4	4	4.83E+06	4	4	0
Q9NRF8|PYRG2_HUMAN	90.54	5	5	5.04E+05	3	1	72.38
Q14254|FLOT2_HUMAN	90.49	25	25	6.21E+06	7	7	101.47
Q9UEG4|ZN629_HUMAN	90.39	5	5	4.70E+06	4	4	0
Q12912|LRMP_HUMAN	90.37	17	17	5.64E+06	5	5	0
O15397|IPO8_HUMAN	90.36	8	8	5.57E+06	5	5	0
P53007|TXTP_HUMAN	90.29	21	21	2.13E+07	6	6	32.19
P81605|DCD_HUMAN	89.99	35	35	1.65E+07	4	4	119.27
Q9P2E9|RRBP1_HUMAN	89.9	6	6	4.32E+06	6	6	0
P25789|PSA4_HUMAN	89.88	15	15	3.83E+07	4	4	91.52
P51665|PSMD7_HUMAN	89.87	23	23	1.81E+07	4	4	0
Q96AB3|ISOC2_HUMAN	89.6	47	47	7.20E+06	4	4	0
O14983|AT2A1_HUMAN	89.55	10	10	5.17E+05	6	2	0
Q14964|RB39A_HUMAN	89.45	21	21	1.74E+06	5	2	0
P48556|PSMD8_HUMAN	89.41	18	18	5.84E+06	6	6	29.43
O43684|BUB3_HUMAN	89.35	25	25	9.69E+06	6	6	62.99
Q9BQ67|GRWD1_HUMAN	89.29	31	31	1.43E+07	9	8	56.28
O94973|AP2A2_HUMAN	89.04	6	6	6.41E+05	4	1	70.4
P52756|RBM5_HUMAN	88.87	15	15	8.66E+06	6	5	77.04
Q9BV38|WDR18_HUMAN	88.83	19	19	1.34E+07	6	6	0
P11171|41_HUMAN	88.73	8	8	1.06E+07	7	6	29.08

Q14CN4|K2C72_HUMAN	88.61	7	7		7	0	72.54
P43490|NAMPT_HUMAN	88.45	22	22	1.52E+07	8	8	0
P61026|RAB10_HUMAN	88.45	18	18	1.60E+06	4	1	44.69
O95299|NDUAA_HUMAN	88.41	26	26	1.73E+07	6	6	52.85
P07355|ANXA2_HUMAN	88.38	18	18	4.91E+06	5	5	0
O60825|F262_HUMAN	88.21	10	10	1.57E+06	4	2	80.26
P35251|RFC1_HUMAN	88.19	7	7	4.50E+06	7	7	91.94
P54136|SYRC_HUMAN	88.19	13	13	1.15E+07	7	7	94.84
Q8IWZ3|ANKH1_HUMAN	88.13	4	4	6.30E+05	5	2	0
Q3SY84|K2C71_HUMAN	88.06	9	9	3.09E+06	9	1	86.64
Q8NHQ9|DDX55_HUMAN	87.8	14	14	4.42E+06	6	6	40.67
P06702|S10A9_HUMAN	87.64	53	53	9.84E+06	4	4	0
O15511|ARPC5_HUMAN	87.59	52	52	6.09E+07	6	6	76.99
Q4V339|CBWD6_HUMAN	87.59	17	17		5	0	0
Q5JTY5|CBWD3_HUMAN	87.59	17	17		5	0	0
P48634|PRC2A_HUMAN	87.53	5	5	2.27E+07	6	6	162.6
P14859|PO2F1_HUMAN	87.38	7	7		6	0	52.15
Q9P2R3|ANFY1_HUMAN	87.28	7	7	4.18E+06	5	5	65.25
Q9NTJ3|SMC4_HUMAN	87.23	9	9	5.29E+06	9	8	0
P56385|ATP5I_HUMAN	87.2	51	51	1.72E+07	3	3	29.84
P36404|ARL2_HUMAN	87.06	30	30	3.54E+06	5	4	0
Q00059|TFAM_HUMAN	87	23	23	8.22E+06	7	7	41.62
Q58FF8|H90B2_HUMAN	86.85	18	18		6	0	0
Q16555|DPYL2_HUMAN	86.44	8	8		3	0	39.21
O00571|DDX3X_HUMAN	86.41	11	11		6	0	192.77
O15523|DDX3Y_HUMAN	86.41	11	11		6	0	178.63
P35659|DEK_HUMAN	86.35	18	18	2.28E+07	6	6	65.85
P0C2W1|FBSP1_HUMAN	86.21	27	27	4.81E+06	4	4	0
Q969G3|SMCE1_HUMAN	86.15	13	13	1.36E+07	4	4	71.72
O60830|TI17B_HUMAN	86.13	47	47	6.27E+06	4	4	0
P41219|PERI_HUMAN	85.84	17	17	4.89E+06	6	3	42.05
O14925|TIM23_HUMAN	85.76	40	40	4.99E+06	3	3	0
Q5SRD1|TI23B_HUMAN	85.76	32	32	4.99E+06	3	3	0
Q9NY93|DDX56_HUMAN	85.75	11	11	2.46E+06	5	5	45.65
P29692|EF1D_HUMAN	85.65	28	28	3.61E+07	8	6	84
Q6NXG1|ESRP1_HUMAN	85.56	5	5	7.14E+05	3	1	0
P62937|PPIA_HUMAN	85.53	49	49	1.42E+07	6	6	89.04
Q7Z3Y7|K1C28_HUMAN	85.5	6	6		4	0	98.48
Q8N5Z5|KCD17_HUMAN	85.22	27	27	2.33E+07	4	4	69.82
Q13868|EXOS2_HUMAN	85.06	19	19	5.45E+06	3	3	0
Q9NTI5|PDS5B_HUMAN	84.97	7	7	1.04E+07	7	6	40.61
Q14137|BOP1_HUMAN	84.82	17	17	1.68E+07	9	9	0
Q7L014|DDX46_HUMAN	84.68	8	8	8.34E+06	7	6	95.81
O95373|IPO7_HUMAN	84.21	8	8	1.13E+07	6	6	28.44
P09661|RU2A_HUMAN	83.83	37	37	1.29E+07	6	6	107.46
P09086|PO2F2_HUMAN	83.57	9	9		5	0	52.15
Q13247|SRSF6_HUMAN	83.55	14	14	3.42E+07	6	3	84.12
P16152|CBR1_HUMAN	83.47	31	31	6.82E+06	6	4	76.48
Q9H0U3|MAGT1_HUMAN	83.44	14	14	1.49E+07	5	5	0
Q9NYF8|BCLF1_HUMAN	83.44	11	11	9.48E+06	7	7	100.73

O60814|H2B1K_HUMAN	83.21	38	38	8.54E+06	5	1	66.34
P57053|H2BFS_HUMAN	83.21	38	38	8.54E+06	5	1	66.34
P58876|H2B1D_HUMAN	83.21	38	38	8.54E+06	5	1	0
P62807|H2B1C_HUMAN	83.21	38	38	8.54E+06	5	1	0
Q5QNW6|H2B2F_HUMAN	83.21	38	38	8.54E+06	5	1	0
Q93079|H2B1H_HUMAN	83.21	38	38	8.54E+06	5	1	0
Q99877|H2B1N_HUMAN	83.21	38	38	8.54E+06	5	1	0
Q99879|H2B1M_HUMAN	83.21	38	38	8.54E+06	5	1	0
P23528|COF1_HUMAN	83.13	44	44	1.33E+07	4	4	91.86
Q96IU4|ABHEB_HUMAN	82.78	28	28	4.48E+06	4	4	0
Q16576|RBBP7_HUMAN	82.49	30	30	8.70E+06	8	3	118.39
P11177|ODPB_HUMAN	82.46	13	13	6.76E+06	5	4	0
Q9Y4W2|LAS1L_HUMAN	82.45	10	10	7.52E+06	5	5	0
Q7Z3Y8|K1C27_HUMAN	82.26	7	7		4	0	84.86
Q9UJX4|APC5_HUMAN	82.25	12	12	5.32E+06	6	6	0
Q5T749|KPRP_HUMAN	82.14	12	12	3.38E+07	6	6	0
P20042|IF2B_HUMAN	81.9	27	27	1.48E+07	7	7	90.22
Q52LJ0|FA98B_HUMAN	81.84	17	17	4.19E+06	5	4	0
Q9UQ88|CD11A_HUMAN	81.64	7	7	5.12E+06	5	4	43.3
Q9BTT6|LRRC1_HUMAN	81.62	10	10	1.84E+06	4	2	0
Q9UH99|SUN2_HUMAN	81.48	12	12	4.71E+06	6	6	77.84
Q9UN81|LORF1_HUMAN	81.39	10	10	4.30E+06	4	4	60.94
P69905|HBA_HUMAN	81.33	35	35	2.82E+07	4	4	67.16
Q9NZI8|IF2B1_HUMAN	80.97	13	13	2.64E+06	5	3	65.24
Q9H0A0|NAT10_HUMAN	80.96	12	12	8.81E+06	8	8	36.96
Q9Y285|SYFA_HUMAN	80.72	26	26	1.20E+07	6	6	30.41
Q9NSB2|KRT84_HUMAN	80.7	5	5	1.77E+05	6	1	82.55
Q969P6|TOP1M_HUMAN	80.54	7	7	0.00E+00	6	1	64.01
Q15233|NONO_HUMAN	80.16	13	13	5.22E+06	5	4	131.94
P51571|SSRD_HUMAN	80.09	31	31	1.50E+07	4	4	0
Q16643|DREB_HUMAN	80.08	12	12	5.21E+06	4	4	0
P27816|MAP4_HUMAN	80.03	9	9	1.12E+06	6	6	47.97
O15145|ARPC3_HUMAN	80	19	19	8.73E+07	3	3	79.32
Q99848|EBP2_HUMAN	79.91	22	22	1.14E+07	5	5	0
Q14315|FLNC_HUMAN	79.71	2	2	1.77E+05	6	2	67.56
O00541|PESC_HUMAN	79.67	8	8	9.03E+06	5	5	0
Q9P035|HACD3_HUMAN	79.56	25	25	2.82E+07	6	5	23.34
Q9UJX5|APC4_HUMAN	79.47	7	7	2.52E+06	4	4	0
O75027|ABCB7_HUMAN	79.32	7	7	2.77E+06	4	4	0
O60701|UGDH_HUMAN	79.31	15	15	3.81E+06	6	6	72.09
O75143|ATG13_HUMAN	79.03	10	10	2.11E+06	4	3	0
O75369|FLNB_HUMAN	78.97	2	2	1.51E+06	6	1	65.97
P24534|EF1B_HUMAN	78.87	27	27	1.37E+06	4	3	59.41
P11717|MPRI_HUMAN	78.85	5	5	6.30E+06	9	9	64.71
P78347|GTF2I_HUMAN	78.6	7	7	5.75E+06	6	6	37.33
P31040|SDHA_HUMAN	78.57	6	6	3.90E+06	3	3	0
Q9Y3F4|STRAP_HUMAN	78.44	19	19	4.57E+06	4	4	75.17
Q96CW1|AP2M1_HUMAN	78.43	22	22	3.23E+07	8	8	81.33
Q3V6T2|GRDN_HUMAN	78.29	4	4	2.79E+06	7	5	52.43
O75477|ERLN1_HUMAN	78.21	20	20	6.43E+05	5	2	37.03

P0C7P4|UCRIL_HUMAN	78.11	30	30	6.99E+06	6	5	41.56
P47985|UCRI_HUMAN	78.11	31	31	6.99E+06	6	5	0
Q95604|1C17_HUMAN	78.04	20	20	5.97E+06	5	1	0
P57088|TMM33_HUMAN	77.82	17	17	7.77E+06	3	3	46.26
Q9BZQ6|EDEM3_HUMAN	77.71	8	8	2.61E+06	5	5	0
P62854|RS26_HUMAN	77.62	34	34	2.33E+08	4	4	72.32
Q99865|SPI2A_HUMAN	77.56	17	17	1.20E+06	3	2	0
Q9BPZ2|SPI2B_HUMAN	77.56	17	17	1.20E+06	3	2	0
P49207|RL34_HUMAN	77.53	22	22	1.33E+08	5	5	77.64
Q05519|SRS11_HUMAN	77.3	12	12	2.53E+07	4	4	89.47
Q15397|PUM3_HUMAN	77.19	10	10	4.42E+06	7	6	0
O75390|CISY_HUMAN	77.05	14	14	3.86E+06	4	4	89.76
A0AVK6|E2F8_HUMAN	76.96	6	6	1.40E+07	6	3	0
Q93009|UBP7_HUMAN	76.95	9	9	1.33E+07	9	9	83.29
O95864|FADS2_HUMAN	76.9	7	7	2.53E+06	2	2	0
Q7Z478|DHX29_HUMAN	76.82	4	4	9.37E+05	4	3	41.44
P19388|RPAB1_HUMAN	76.8	16	16	2.34E+06	3	3	0
P28340|DPOD1_HUMAN	76.75	5	5	1.72E+06	5	4	0
Q96L58|B3GT6_HUMAN	76.64	11	11	1.25E+06	3	3	0
B2RXH8|HNRC2_HUMAN	76.55	8	8		3	0	117.39
B7ZW38|HNRC3_HUMAN	76.55	8	8		3	0	123.77
O60812|HNRC1_HUMAN	76.55	8	8		3	0	123.77
P0DMR1|HNRC4_HUMAN	76.55	8	8		3	0	123.77
O60716|CTND1_HUMAN	76.45	6	6	3.88E+06	5	5	0
Q9UJV9|DDX41_HUMAN	76.29	14	14	4.61E+06	5	5	57.57
Q13619|CUL4A_HUMAN	76.24	10	10	1.03E+06	5	2	21.94
P62820|RAB1A_HUMAN	76.07	12	12		3	0	0
Q92930|RAB8B_HUMAN	76.07	12	12		3	0	0
Q9NS69|TOM22_HUMAN	76.07	43	43	7.51E+06	3	3	0
Q96DA2|RB39B_HUMAN	76.03	11	11		3	0	0
Q9UN37|VPS4A_HUMAN	75.92	13	13	5.03E+06	4	3	21.8
tr|I3L4J1|I3L4J1_HUMAN	75.92	14	14	5.03E+06	4	3	0
Q14692|BMS1_HUMAN	75.69	5	5	2.24E+06	5	5	80.5
Q9UG63|ABCF2_HUMAN	75.68	13	13	2.67E+07	8	8	0
O00743|PPP6_HUMAN	75.65	20	20	1.52E+07	4	4	91.49
P30101|PDIA3_HUMAN	75.65	15	15	6.19E+06	6	6	120.3
P08237|PFKAM_HUMAN	75.57	7	7	1.95E+06	4	2	23.51
Q5T4S7|UBR4_HUMAN	75.54	1	1	1.71E+06	5	5	34.61
tr|S4R435|S4R435_HUMAN	75.47	9	9		3	0	65.02
Q13162|PRDX4_HUMAN	75.15	25	25	2.78E+06	5	3	88.12
Q96GM8|TOE1_HUMAN	75.08	12	12	5.34E+06	4	4	54.71
Q9BZJ0|CRNL1_HUMAN	75.05	7	7	4.76E+06	6	6	44.44
O94905|ERLN2_HUMAN	74.91	13	13	8.38E+05	4	1	48.57
P78559|MAP1A_HUMAN	74.84	2	2	2.15E+06	4	3	56.47
Q969X5|ERGI1_HUMAN	74.83	14	14	6.06E+06	4	4	0
Q96GM5|SMRD1_HUMAN	74.78	6	6	1.56E+06	2	1	0
Q86U42|PABP2_HUMAN	74.62	24	24	8.60E+06	4	4	80.81
P0C0S5|H2AZ_HUMAN	74.61	46	46	6.86E+06	4	2	68.1
Q71UI9|H2AV_HUMAN	74.61	46	46	6.86E+06	4	2	68.1
P53634|CATC_HUMAN	74.6	13	13	3.36E+06	3	3	0

Q9Y6M1|IF2B2_HUMAN	74.54	7	7	5.31E+05	3	1	82.41
Q9BVP2|GNL3_HUMAN	74.52	21	21	1.52E+07	8	8	74.55
Q9UKV8|AGO2_HUMAN	74.37	8	8	2.13E+06	6	3	60.41
Q9H0C2|ADT4_HUMAN	74.16	9	9		3	0	69.42
P25398|RS12_HUMAN	73.78	39	39	8.45E+07	4	4	82.77
O00487|PSDE_HUMAN	73.73	19	19	1.19E+07	4	4	0
Q9NZB2|F120A_HUMAN	73.73	6	6	5.94E+06	6	6	100.42
P31153|METK2_HUMAN	73.7	16	16	6.18E+06	5	4	75.7
Q13620|CUL4B_HUMAN	73.57	8	8	2.14E+06	6	2	0
Q9NZ01|TECR_HUMAN	73.5	19	19	2.96E+07	6	6	0
P14136|GFAP_HUMAN	73.47	4	4		3	0	63.88
Q13148|TADBP_HUMAN	73.46	12	12	3.63E+06	3	3	25.78
P49821|NDUV1_HUMAN	73.37	15	15	5.02E+06	4	4	27.97
Q5SY16|NOL9_HUMAN	73.35	20	20	1.54E+07	7	7	0
P46087|NOP2_HUMAN	73.29	12	12	7.19E+06	8	8	0
O15269|SPTC1_HUMAN	73.22	12	12	6.10E+06	4	4	0
Q9H082|RB33B_HUMAN	73.22	10	10	2.56E+05	3	1	0
Q15008|PSMD6_HUMAN	73.06	15	15	8.40E+06	5	5	74.75
Q9H307|PININ_HUMAN	72.94	7	7	4.75E+06	4	4	79.78
O15042|SR140_HUMAN	72.85	9	9	8.50E+06	7	7	156.07
P06899|H2B1J_HUMAN	72.66	38	38		6	0	60.74
P23527|H2B1O_HUMAN	72.66	38	38		6	0	0
P33778|H2B1B_HUMAN	72.66	38	38		6	0	0
Q16778|H2B2E_HUMAN	72.66	38	38		6	0	0
Q99456|K1C12_HUMAN	72.64	4	4		4	0	69.97
Q9P265|DIP2B_HUMAN	72.63	6	6	4.95E+06	7	7	44.78
P27694|RFA1_HUMAN	72.62	9	9	2.73E+06	4	4	0
Q13523|PRP4B_HUMAN	72.57	4	4	6.40E+05	4	4	23.74
O95232|LC7L3_HUMAN	72.54	15	15	2.43E+07	6	6	84.76
P30043|BLVRB_HUMAN	72.41	45	45	2.70E+06	4	4	0
Q9UPN7|PP6R1_HUMAN	72.32	10	10	2.02E+07	4	4	0
Q8NFW8|NEUA_HUMAN	72.07	12	12	5.71E+06	3	3	0
Q96SI9|STRBP_HUMAN	72.06	5	5		3	0	50.26
O00507|USP9Y_HUMAN	72.03	2	2		4	0	0
Q12904|AIMP1_HUMAN	71.94	26	26	6.18E+06	4	4	61.2
Q9UBS4|DJB11_HUMAN	71.8	15	15	3.04E+06	3	3	0
P35232|PHB_HUMAN	71.7	32	32	7.51E+06	5	5	44.62
P13804|ETFA_HUMAN	71.69	25	25	4.25E+06	4	4	0
O14735|CDIPT_HUMAN	71.56	16	16	3.60E+06	3	3	0
Q31612|1B73_HUMAN	71.54	16	16		4	0	0
O60884|DNJA2_HUMAN	71.53	9	9	2.73E+06	3	3	0
Q7L1I2|SV2B_HUMAN	71.53	9	9	7.39E+06	5	5	60.04
Q9Y3D7|TIM16_HUMAN	71.34	35	35	4.94E+06	3	3	0
Q9NUQ6|SPS2L_HUMAN	70.95	5	5	3.89E+06	3	3	29.31
P47897|SYQ_HUMAN	70.93	10	10	3.78E+06	7	6	62.6
Q5C9Z4|NOM1_HUMAN	70.73	8	8	4.17E+06	5	5	0
P04637|P53_HUMAN	70.61	8	8	1.35E+06	2	2	33.31
Q86YP4|P66A_HUMAN	70.51	12	12	8.43E+06	5	4	0
O00160|MYO1F_HUMAN	70.48	3	3		4	0	0
Q14103|HNRPD_HUMAN	70.47	9	9	1.76E+06	4	1	103.67

O15372|EIF3H_HUMAN	70.44	21	21	7.99E+06	5	5	0
P84085|ARF5_HUMAN	70.38	36	36	8.41E+06	6	2	0
O60313|OPA1_HUMAN	70.14	6	6	2.33E+06	5	5	62.34
O00264|PGRC1_HUMAN	70.11	43	43	5.34E+06	5	5	0
Q9BRL6|SRSF8_HUMAN	70.01	11	11	3.87E+05	3	1	0
Q9NUD5|ZCHC3_HUMAN	70.01	15	15	1.47E+06	5	4	98.02
Q00535|CDK5_HUMAN	69.87	12	12	1.18E+06	4	2	0
P14678|RSMB_HUMAN	69.78	28	28	2.80E+06	4	1	57.69
Q15024|EXOS7_HUMAN	69.74	26	26	3.77E+06	5	5	0
O75947|ATP5H_HUMAN	69.67	34	34	1.15E+07	4	4	0
Q8WXF1|PSPC1_HUMAN	69.59	12	12	5.70E+06	4	4	22.36
Q8WZ42|TITIN_HUMAN	69.57	1	1	5.86E+06	14	10	58.51
Q15717|ELAV1_HUMAN	69.43	14	14	3.83E+06	3	3	141.8
P28370|SMCA1_HUMAN	69.4	5	5		6	0	0
Q8NBX0|SCPDL_HUMAN	69.4	21	21	3.19E+06	5	5	0
Q96DI7|SNR40_HUMAN	69.4	26	26	5.66E+06	5	4	0
P40937|RFC5_HUMAN	69.21	20	20	4.19E+06	4	4	96.35
P51531|SMCA2_HUMAN	69.15	6	6	1.14E+06	9	3	0
Q53H12|AGK_HUMAN	69.12	22	22	9.76E+06	5	5	36.9
Q9H857|NT5D2_HUMAN	69.12	21	21	7.93E+06	7	7	0
A5YKK6|CNOT1_HUMAN	68.98	3	3	3.11E+07	5	5	48.95
Q5VWQ0|RSBN1_HUMAN	68.75	5	5	3.48E+06	3	3	56.89
O75367|H2AY_HUMAN	68.46	12	12	3.29E+05	2	1	57.54
O94776|MTA2_HUMAN	68.16	8	8	3.92E+06	5	4	107.56
P02768|ALBU_HUMAN	68.03	9	9	1.00E+07	3	3	113.63
Q8IWI9|MGAP_HUMAN	67.86	3	3	4.00E+06	7	6	87.8
P48729|KC1A_HUMAN	67.8	9	9	1.98E+06	2	2	68.28
O75063|XYLK_HUMAN	67.54	12	12	1.99E+06	3	3	0
Q00526|CDK3_HUMAN	67.47	12	12	4.31E+05	3	1	0
Q13330|MTA1_HUMAN	67.34	6	6	1.37E+06	4	2	61.78
P16278|BGAL_HUMAN	67.33	12	12	4.64E+06	5	5	63.45
Q9ULX6|AKP8L_HUMAN	67.28	6	6	6.43E+06	3	3	20.3
Q7L2H7|EIF3M_HUMAN	67.24	15	15	8.11E+06	4	4	59.43
Q96EY1|DNJA3_HUMAN	67.05	9	9	2.73E+06	3	3	0
Q06265|EXOS9_HUMAN	67.03	5	5	1.88E+06	2	2	47.97
Q9Y241|HIG1A_HUMAN	66.94	24	24	2.92E+06	1	1	0
tr|C9JAW5|C9JAW5_HUMAN	66.94	27	27	2.92E+06	1	1	0
P00367|DHE3_HUMAN	66.89	14	14	3.47E+06	5	2	0
Q9H254|SPTN4_HUMAN	66.88	2	2		6	0	79.97
Q9H6T0|ESRP2_HUMAN	66.85	5	5	1.45E+05	3	1	0
Q08378|GOGA3_HUMAN	66.56	5	5	2.49E+06	5	5	152.6
P28288|ABCD3_HUMAN	66.45	8	8	5.79E+06	6	5	47.01
Q12931|TRAP1_HUMAN	66.44	12	12	9.65E+04	4	3	46.41
O76031|CLPX_HUMAN	66.07	7	7	3.80E+06	3	3	0
P18077|RL35A_HUMAN	65.93	29	29	1.71E+08	6	6	83.29
P62258|1433E_HUMAN	65.8	25	25	2.36E+07	4	3	0
O00411|RPOM_HUMAN	65.67	7	7	4.28E+06	6	6	20.78
Q96A65|EXOC4_HUMAN	65.67	8	8	3.03E+06	5	5	0
Q9UKU7|ACAD8_HUMAN	65.57	8	8	4.37E+06	3	3	0
O00422|SAP18_HUMAN	65.52	26	26	5.07E+06	4	4	0

O75362|ZN217_HUMAN	65.5	4	4	1.26E+06	4	3	0
Q5D862|FILA2_HUMAN	65.27	4	4	1.32E+07	3	3	35.15
Q5VT06|CE350_HUMAN	65.22	3	3	2.90E+06	5	5	80.74
Q9NX63|MIC19_HUMAN	65.18	18	18	3.81E+06	3	3	0
Q7L311|ARMX2_HUMAN	65.07	7	7	2.54E+06	4	4	0
Q8WUY1|THEM6_HUMAN	64.98	17	17	4.23E+06	3	3	0
P20340|RAB6A_HUMAN	64.95	12	12	7.46E+05	2	1	0
P62995|TRA2B_HUMAN	64.94	24	24	1.90E+07	6	5	85.74
P42025|ACTY_HUMAN	64.92	23	23		4	0	0
O95716|RAB3D_HUMAN	64.87	6	6		2	0	0
P20336|RAB3A_HUMAN	64.87	6	6		2	0	0
Q96E17|RAB3C_HUMAN	64.87	6	6		2	0	0
P53597|SUCA_HUMAN	64.86	18	18	7.09E+06	5	5	0
Q9Y512|SAM50_HUMAN	64.74	19	19	5.82E+06	5	4	0
P40616|ARL1_HUMAN	64.57	17	17	4.41E+06	2	2	0
Q96P70|IPO9_HUMAN	64.46	4	4	4.06E+06	3	3	0
P61204|ARF3_HUMAN	64.42	30	30	2.27E+06	5	1	0
P84077|ARF1_HUMAN	64.42	30	30	2.27E+06	5	1	0
tr|F5H423|F5H423_HUMAN	64.42	26	26	2.27E+06	5	1	0
Q05823|RN5A_HUMAN	64.4	5	5	3.37E+06	4	4	0
Q9UM00|TMCO1_HUMAN	64.38	20	20	1.04E+07	3	3	39.1
Q9Y5M8|SRPRB_HUMAN	64.24	17	17	1.81E+06	3	3	0
P49458|SRP09_HUMAN	64.22	31	31	9.45E+06	3	3	0
O75534|CSDE1_HUMAN	64.13	5	5	4.10E+06	5	5	49
P63162|RSMN_HUMAN	64.1	12	12		3	0	52.81
Q92974|ARHG2_HUMAN	64.04	9	9	9.30E+06	5	5	169.4
P63167|DYL1_HUMAN	64.01	27	27	1.32E+07	3	2	30.82
Q7Z4V5|HDGR2_HUMAN	63.86	17	17	4.21E+06	6	6	0
P12268|IMDH2_HUMAN	63.72	10	10	4.60E+06	3	3	95.85
P11166|GTR1_HUMAN	63.63	6	6	6.37E+06	5	4	25.23
Q8WXG6|MADD_HUMAN	63.61	4	4	1.49E+06	4	4	0
O15381|NVL_HUMAN	63.46	2	2	3.28E+05	1	1	0
O94805|ACL6B_HUMAN	63.44	13	13	3.30E+06	4	3	0
Q9NYK5|RM39_HUMAN	63.22	11	11	1.11E+06	3	3	0
Q14258|TRI25_HUMAN	63.15	8	8	1.10E+06	4	4	34.27
Q9Y6C9|MTCH2_HUMAN	63.14	18	18	1.28E+07	4	4	56.32
Q6N021|TET2_HUMAN	63.13	2	2	1.29E+06	4	2	26.01
P08865|RSSA_HUMAN	63.12	18	18	7.19E+06	4	4	92.29
Q99459|CDC5L_HUMAN	63.11	5	5	3.37E+06	3	3	85.49
Q13155|AIMP2_HUMAN	63.06	17	17	3.44E+06	3	3	0
Q8N6R0|MET13_HUMAN	63	5	5	1.57E+06	3	3	0
Q9UKM9|RALY_HUMAN	62.87	25	25	1.53E+07	5	5	105.91
P60174|TPIS_HUMAN	62.86	13	13	2.84E+05	2	2	0
O14647|CHD2_HUMAN	62.78	2	2		3	0	67.05
Q08380|LG3BP_HUMAN	62.69	9	9	1.04E+07	4	4	0
P45880|VDAC2_HUMAN	62.66	10	10	4.23E+06	3	3	59.22
Q04837|SSBP_HUMAN	62.58	36	36	6.01E+06	4	4	50.73
Q5UIP0|RIF1_HUMAN	62.57	2	2	8.87E+05	3	3	49.63
O75400|PR40A_HUMAN	62.53	7	7	1.55E+07	5	5	117.92
Q9P2E5|CHPF2_HUMAN	62.42	5	5	5.58E+05	2	2	0

Q01780|EXOSX_HUMAN	62.34	6	6	1.01E+07	3	3	59.62
Q9Y2T4|2ABG_HUMAN	62.34	5	5		3	0	0
Q5JTZ9|SYAM_HUMAN	62.29	4	4	1.32E+06	3	3	0
P24941|CDK2_HUMAN	62.26	6	6		2	0	0
P09429|HMGB1_HUMAN	62.18	14	14	9.54E+06	2	2	66.23
Q9Y5Q8|TF3C5_HUMAN	62.18	13	13	3.92E+06	5	5	0
Q13015|AF1Q_HUMAN	62.17	76	76	1.84E+06	3	3	0
Q92750|TAF4B_HUMAN	61.87	4	4	0.00E+00	3	1	0
Q07021|C1QBP_HUMAN	61.85	17	17	1.96E+07	2	2	0
Q86XI2|CNDG2_HUMAN	61.85	4	4	1.40E+06	4	3	0
P38935|SMBP2_HUMAN	61.8	6	6	2.08E+06	3	3	21.26
Q8N8A2|ANR44_HUMAN	61.8	4	4		3	0	69.9
Q12800|TFCP2_HUMAN	61.6	7	7	1.93E+05	2	1	0
Q9NXV2|KCTD5_HUMAN	61.56	21	21	4.27E+07	3	2	103.37
P60900|PSA6_HUMAN	61.46	13	13	1.84E+06	2	2	0
Q13144|EI2BE_HUMAN	61.43	7	7	2.35E+06	3	3	0
Q10570|CPSF1_HUMAN	61.32	3	3	1.78E+06	4	4	37.64
P62140|PP1B_HUMAN	61.26	6	6	3.57E+05	2	1	25.26
O76094|SRP72_HUMAN	61.2	7	7	1.43E+06	3	3	0
Q9UJW0|DCTN4_HUMAN	61.18	6	6	1.44E+06	2	2	0
P62158|CALM_HUMAN	61.1	62	62	1.15E+07	4	4	0
Q6STE5|SMRD3_HUMAN	60.97	4	4		1	0	0
Q92925|SMRD2_HUMAN	60.97	4	4		1	0	33.12
Q9UBV2|SE1L1_HUMAN	60.92	4	4	1.47E+06	2	2	0
P04899|GNAI2_HUMAN	60.85	13	13	4.78E+05	4	1	0
Q5RKV6|EXOS6_HUMAN	60.85	10	10	2.28E+06	2	2	66.6
O95983|MBD3_HUMAN	60.76	16	16	1.94E+06	3	3	0
O15075|DCLK1_HUMAN	60.49	7	7	2.89E+06	4	3	46.49
Q9BW92|SYTM_HUMAN	60.49	7	7	2.83E+06	3	3	0
Q9NSD9|SYFB_HUMAN	60.48	7	7	3.43E+06	3	3	72.02
Q9H1K4|GHC2_HUMAN	60.46	12	12		4	0	0
Q96FJ2|DYL2_HUMAN	60.42	27	27	1.36E+06	2	1	0
P08574|CY1_HUMAN	60.34	9	9	2.23E+07	3	3	45.48
Q13490|BIRC2_HUMAN	60.28	9	9	3.56E+06	4	4	0
Q66PJ3|AR6P4_HUMAN	60.24	7	7	2.60E+06	2	2	56.26
Q96P63|SPB12_HUMAN	60.14	7	7	1.31E+06	3	3	0
O14967|CLGN_HUMAN	60.01	10	10	1.69E+06	3	3	0
P43307|SSRA_HUMAN	59.84	8	8	1.24E+07	2	2	0
O75912|DGKI_HUMAN	59.83	4	4	2.40E+05	2	1	0
Q86UE4|LYRIC_HUMAN	59.82	10	10	5.63E+06	4	4	69.76
Q9Y3Z3|SAMH1_HUMAN	59.81	10	10	5.21E+06	5	5	0
P60660|MYL6_HUMAN	59.73	27	27	1.14E+07	3	3	78.9
P40938|RFC3_HUMAN	59.7	7	7	2.95E+06	2	2	36.61
Q8TBX8|PI42C_HUMAN	59.62	13	13	3.15E+06	4	4	0
Q8IXI1|MIRO2_HUMAN	59.47	6	6	9.57E+04	3	1	30.81
Q6ZRV2|FA83H_HUMAN	59.42	5	5	1.91E+06	3	3	82.06
P12074|CX6A1_HUMAN	59.21	36	36	3.38E+06	2	2	0
tr|H0YIV9|H0YIV9_HUMAN	59.21	23	23	3.38E+06	2	2	0
O00139|KIF2A_HUMAN	59.13	3	3	5.05E+05	2	2	0
Q13428|TCOF_HUMAN	59.1	3	3	5.80E+07	4	4	26.97

P84103|SRSF3_HUMAN	58.97	24	24	3.44E+07	5	4	68.15
P51153|RAB13_HUMAN	58.94	12	12	5.42E+05	2	1	0
P23526|SAHH_HUMAN	58.9	7	7	1.50E+06	3	3	87.08
Q08170|SRSF4_HUMAN	58.89	9	9	3.62E+06	4	2	60.93
P33121|ACSL1_HUMAN	58.79	8	8	1.12E+06	4	3	0
P32322|P5CR1_HUMAN	58.76	9	9	8.23E+05	2	2	0
Q9H269|VPS16_HUMAN	58.76	4	4	1.05E+06	2	2	0
Q6P158|DHX57_HUMAN	58.51	3	3	1.66E+06	4	3	76.09
P10644|KAP0_HUMAN	58.36	6	6	3.49E+05	2	1	0
O75828|CBR3_HUMAN	58.22	9	9		2	0	0
Q8IXB1|DJC10_HUMAN	57.89	7	7	3.01E+06	4	3	42.6
Q9BPX5|ARP5L_HUMAN	57.83	33	33	8.10E+06	2	2	57.54
Q9H0S4|DDX47_HUMAN	57.82	12	12	1.01E+06	4	3	0
Q56VL3|OCAD2_HUMAN	57.66	14	14	6.95E+06	2	2	43.66
Q9Y4D8|HECD4_HUMAN	57.62	1	1	1.07E+06	3	2	52.02
P24539|AT5F1_HUMAN	57.56	20	20	1.94E+07	4	4	0
O43933|PEX1_HUMAN	57.46	2	2	0.00E+00	2	1	31.83
Q8TEQ6|GEMI5_HUMAN	57.41	3	3	3.55E+07	3	3	0
P0DN79|CBSL_HUMAN	57.34	17	17	2.72E+06	4	3	39.21
P35520|CBS_HUMAN	57.34	17	17	2.72E+06	4	3	39.21
Q9Y2T2|AP3M1_HUMAN	57.31	15	15	1.41E+06	5	3	0
Q9BSJ2|GCP2_HUMAN	57.18	4	4	1.77E+06	3	3	0
Q8IZQ5|SELH_HUMAN	57.08	34	34	2.44E+06	3	3	39.61
Q92820|GGH_HUMAN	57.08	12	12	1.97E+06	2	2	0
O95816|BAG2_HUMAN	56.83	24	24	8.17E+06	4	4	79.45
O14617|AP3D1_HUMAN	56.79	3	3	1.43E+06	2	2	0
Q14527|HLTF_HUMAN	56.64	2	2	7.25E+05	2	2	0
P61619|S61A1_HUMAN	56.63	8	8	2.35E+06	2	2	0
A0FGR8|ESYT2_HUMAN	56.49	5	5	1.62E+06	3	3	0
Q9UH62|ARMX3_HUMAN	56.45	8	8	5.07E+06	3	3	36.04
Q8N163|CCAR2_HUMAN	56.31	8	8	1.81E+06	4	4	177.85
Q9UL18|AGO1_HUMAN	56.31	4	4	3.90E+05	3	1	40.19
P50336|PPOX_HUMAN	56.26	10	10	1.20E+06	3	3	0
Q7LGA3|HS2ST_HUMAN	56.19	16	16	1.53E+06	3	3	0
P07686|HEXB_HUMAN	56.11	11	11	2.88E+06	4	4	0
Q16836|HCDH_HUMAN	56.1	14	14	8.97E+05	3	3	0
Q96S59|RANB9_HUMAN	55.95	3	3	2.00E+06	2	2	23.49
P20648|ATP4A_HUMAN	55.89	3	3		3	0	29.46
Q9ULK5|VANG2_HUMAN	55.67	4	4	4.20E+05	2	2	0
Q96EY7|PTCD3_HUMAN	55.62	5	5	1.37E+06	3	3	86.92
Q96P50|ACAP3_HUMAN	55.61	4	4	1.18E+06	3	1	0
Q03393|PTPS_HUMAN	55.42	32	32	7.91E+06	3	3	0
Q9GZL7|WDR12_HUMAN	55.41	14	14	3.07E+06	3	3	0
Q9Y2P8|RCL1_HUMAN	55.39	8	8	1.79E+06	3	3	34.27
O95400|CD2B2_HUMAN	55.25	6	6	1.44E+06	1	1	42.23
P05109|S10A8_HUMAN	55.16	34	34	7.98E+06	2	2	0
Q13242|SRSF9_HUMAN	55.15	8	8	1.12E+06	2	1	75.58
P08579|RU2B_HUMAN	55.13	29	29	2.70E+06	4	4	105.65
P50213|IDH3A_HUMAN	54.96	7	7	1.28E+06	2	2	0
P60981|DEST_HUMAN	54.88	10	10	7.08E+05	1	1	0

P50851|LRBA_HUMAN	54.84	1	1	3.44E+05	3	2	0
Q15771|RAB30_HUMAN	54.78	16	16	0.00E+00	2	1	0
Q8WUA4|TF3C2_HUMAN	54.7	7	7	3.91E+06	4	3	23.04
Q96JP5|ZFP91_HUMAN	54.69	13	13	1.23E+06	3	3	0
P61513|RL37A_HUMAN	54.66	28	28	2.14E+07	2	2	0
Q15149|PLEC_HUMAN	54.57	1	1	4.00E+05	3	1	62.51
O43264|ZW10_HUMAN	54.56	3	3	9.09E+05	2	2	0
Q8WVV4|POF1B_HUMAN	54.54	8	8	2.19E+06	3	3	22.64
Q13098|CSN1_HUMAN	54.48	6	6	4.45E+05	1	1	0
Q9UDR5|AASS_HUMAN	54.44	6	6	2.40E+06	3	3	0
A4D1E9|GTPBA_HUMAN	54.42	9	9	1.04E+06	3	3	0
P38117|ETFB_HUMAN	54.39	16	16	2.54E+06	3	3	0
Q13438|OS9_HUMAN	54.27	9	9	3.90E+06	4	4	0
P08754|GNAI3_HUMAN	54.23	9	9		3	0	0
P63096|GNAI1_HUMAN	54.23	9	9		3	0	0
O95785|WIZ_HUMAN	54.21	6	6	5.45E+06	3	3	0
Q9NZI7|UBIP1_HUMAN	54.15	6	6	1.23E+05	2	1	0
P04792|HSPB1_HUMAN	54.14	24	24	3.99E+06	3	3	0
Q9H0Q0|FA49A_HUMAN	54.03	9	9	1.06E+06	2	2	0
Q9Y277|VDAC3_HUMAN	54.03	8	8	3.99E+05	2	1	0
P18583|SON_HUMAN	54.02	2	2	1.65E+06	3	3	0
Q9Y3L5|RAP2C_HUMAN	53.88	11	11	1.16E+06	2	1	0
P51991|ROA3_HUMAN	53.86	9	9	7.71E+06	3	3	123.63
Q96DU9|PABP5_HUMAN	53.78	3	3		1	0	52.88
Q96EQ8|RN125_HUMAN	53.64	12	12	0.00E+00	2	1	0
O14979|HNRDL_HUMAN	53.6	2	2		2	0	84.93
Q9H0E2|TOLIP_HUMAN	53.58	5	5	4.07E+05	2	1	45.71
Q96BW9|TAM41_HUMAN	53.56	10	10	1.87E+06	3	3	0
A1L020|MEX3A_HUMAN	53.36	5	5	4.76E+05	2	1	79.4
O95168|NDUB4_HUMAN	53.33	39	39	1.10E+07	4	4	46.13
P12532|KCRU_HUMAN	53.23	7	7	2.03E+06	2	2	0
P62136|PP1A_HUMAN	53.19	3	3		1	0	0
Q96FW1|OTUB1_HUMAN	53.19	10	10	1.18E+06	2	2	29.68
Q86W42|THOC6_HUMAN	53.14	7	7	8.61E+05	2	1	0
Q9BT88|SYT11_HUMAN	53.1	5	5	2.68E+05	2	2	0
P31930|QCR1_HUMAN	53.04	12	12	2.30E+06	3	3	0
Q9BRX2|PELO_HUMAN	52.71	9	9	4.23E+06	2	2	0
P36957|ODO2_HUMAN	52.61	7	7	1.73E+06	3	3	57.25
Q15287|RNPS1_HUMAN	52.57	19	19	1.49E+07	3	3	66.28
P19474|RO52_HUMAN	52.55	4	4	3.82E+06	2	2	0
Q92542|NICA_HUMAN	52.51	6	6	2.78E+06	3	3	0
Q6PGP7|TTC37_HUMAN	52.4	1	1	3.98E+05	1	1	0
Q96I25|SPF45_HUMAN	52.37	8	8	1.87E+06	4	3	114.79
Q8N1I0|DOCK4_HUMAN	52.25	1	1	3.46E+05	2	2	0
P47914|RL29_HUMAN	52.18	15	15	1.24E+08	3	3	66.32
Q9BW72|HIG2A_HUMAN	52.15	20	20	1.05E+06	1	1	0
P49448|DHE4_HUMAN	52.07	8	8		3	0	0
Q86U38|NOP9_HUMAN	52.02	8	8	3.04E+06	3	3	43.48
Q5JQF8|PAP1M_HUMAN	51.92	5	5		2	0	71.81
P18085|ARF4_HUMAN	51.9	26	26	5.70E+06	4	1	0

Q9NVH1|DJC11_HUMAN	51.89	4	4	2.85E+06	2	2	59.59
P46060|RAGP1_HUMAN	51.88	8	8	2.00E+06	3	3	0
P48645|NMU_HUMAN	51.87	21	21	3.55E+06	2	2	0
Q16880|CGT_HUMAN	51.76	6	6	9.21E+05	3	3	0
P23246|SFPQ_HUMAN	51.73	7	7	1.59E+06	4	3	141.77
Q9H019|MFR1L_HUMAN	51.43	11	11	1.39E+06	2	2	0
P08670|VIME_HUMAN	51.39	8	8	8.89E+04	3	1	28.66
O60645|EXOC3_HUMAN	51.38	3	3	9.82E+05	2	2	0
Q14894|CRYM_HUMAN	51.36	9	9	1.28E+06	2	2	40.84
Q9NV70|EXOC1_HUMAN	51.23	6	6	2.88E+06	3	3	0
Q16718|NDUA5_HUMAN	51.22	22	22	2.32E+06	2	2	0
P13861|KAP2_HUMAN	51.16	10	10	7.66E+05	3	2	78.12
A0AV96|RBM47_HUMAN	51.13	11	11	3.04E+06	4	4	68.79
P35269|T2FA_HUMAN	51.12	10	10	1.25E+06	3	3	0
P62304|RUXE_HUMAN	51.09	29	29	1.10E+07	3	3	110.09
Q5VTJ3|KLD7A_HUMAN	51.09	9	9	5.81E+05	2	2	0
Q00341|VIGLN_HUMAN	51.04	2	2	4.91E+05	2	2	21.78
P17612|KAPCA_HUMAN	50.99	8	8	1.04E+06	2	2	23.54
Q9BVJ7|DUS23_HUMAN	50.83	9	9	6.70E+05	1	1	0
O00716|E2F3_HUMAN	50.74	3	3	4.04E+05	1	1	0
tr|A0A075B6S2|A0A075B6S2	50.7	17	17	5.57E+07	3	3	104.85
tr|A0A0A0MRZ7|A0A0A0MR	50.7	17	17	5.57E+07	3	3	104.85
Q8NCA5|FA98A_HUMAN	50.66	5	5	3.58E+07	2	1	0
Q99805|TM9S2_HUMAN	50.66	2	2	7.21E+05	1	1	0
P42695|CNDD3_HUMAN	50.56	2	2	1.68E+06	2	2	0
Q6NUK1|SCMC1_HUMAN	50.55	4	4	8.46E+05	2	1	0
Q9BW19|KIFC1_HUMAN	50.48	3	3		2	0	46.37
P37802|TAGL2_HUMAN	50.47	6	6	3.98E+05	1	1	61.51
Q9Y6U3|ADSV_HUMAN	50.36	9	9	2.76E+06	4	4	73.7
O75351|VPS4B_HUMAN	50.3	5	5	4.44E+05	2	1	0
Q9NWT1|PK1IP_HUMAN	50.27	9	9	1.80E+06	2	2	0
Q9NQT4|EXOS5_HUMAN	50.13	9	9	2.96E+06	2	2	0
O60674|JAK2_HUMAN	49.92	1	1		2	0	53.84
P54707|AT12A_HUMAN	49.85	2	2		2	0	0
Q8IUR7|ARMC8_HUMAN	49.71	4	4	7.08E+05	2	2	0
P68400|CSK21_HUMAN	49.62	10	10	6.80E+06	2	2	76.93
Q8NEV1|CSK23_HUMAN	49.62	10	10	6.80E+06	2	2	0
Q9NU22|MDN1_HUMAN	49.57	1	1	1.09E+06	4	4	0
P57678|GEMI4_HUMAN	49.47	2	2	6.87E+05	2	1	0
Q96AY4|TTC28_HUMAN	49.47	1	1	5.25E+05	4	2	74.57
Q9NRX2|RM17_HUMAN	49.31	9	9	2.94E+05	1	1	0
O75122|CLAP2_HUMAN	49.25	2	2	1.15E+06	2	2	93.9
Q6PD62|CTR9_HUMAN	49.23	6	6	3.36E+06	4	4	0
P26196|DDX6_HUMAN	49.2	8	8	9.19E+05	3	2	78.67
P49761|CLK3_HUMAN	49.11	5	5	4.41E+05	2	2	0
Q12933|TRAF2_HUMAN	49.11	5	5	5.84E+05	2	2	0
Q8IXI2|MIRO1_HUMAN	49.06	4	4	9.24E+05	2	1	0
Q9NUQ2|PLCE_HUMAN	49.01	3	3	1.94E+06	1	1	0
O00267|SPT5H_HUMAN	48.96	2	2	9.14E+05	2	2	0
O43776|SYNC_HUMAN	48.94	3	3	9.87E+05	2	1	0

P15056|BRAF_HUMAN	48.94	3	3	0.00E+00	2	1	0
Q13418|ILK_HUMAN	48.74	3	3	3.49E+05	1	1	0
O75323|NIPS2_HUMAN	48.62	18	18	3.37E+06	3	2	67.54
Q8N1F7|NUP93_HUMAN	48.52	8	8	2.23E+06	3	3	0
Q96BN8|OTUL_HUMAN	48.44	11	11	2.88E+05	2	2	0
Q13042|CDC16_HUMAN	48.34	5	5	2.46E+06	2	2	0
P05091|ALDH2_HUMAN	48.3	5	5	5.58E+05	2	1	28.97
Q4G0P3|HYDIN_HUMAN	48.27	1	1	2.35E+06	3	2	0
O00391|QSOX1_HUMAN	48.26	7	7	2.08E+06	3	3	0
Q15046|SYK_HUMAN	48.16	6	6	3.87E+06	2	2	0
Q8IWX8|CHERP_HUMAN	48.05	4	4	2.11E+06	3	3	134.61
Q9H078|CLPB_HUMAN	47.97	5	5	1.07E+06	2	2	0
Q9Y463|DYR1B_HUMAN	47.85	4	4	1.51E+06	3	3	0
O00471|EXOC5_HUMAN	47.83	4	4	2.64E+05	1	1	31.45
Q9H0E3|SP130_HUMAN	47.79	2	2	9.64E+05	2	2	0
P36915|GNL1_HUMAN	47.69	2	2	5.69E+05	1	1	0
Q86UQ4|ABCAD_HUMAN	47.55	0	0		3	0	0
O43707|ACTN4_HUMAN	47.53	4	4		3	0	90.41
Q92828|COR2A_HUMAN	47.51	5	5	2.11E+06	3	3	0
P46940|IQGA1_HUMAN	47.5	3	3	8.79E+05	3	3	83.23
Q658Y4|F91A1_HUMAN	47.49	2	2	1.78E+06	1	1	73.6
P51648|AL3A2_HUMAN	47.48	4	4	7.92E+05	2	1	0
P63010|AP2B1_HUMAN	47.42	6	6	3.55E+06	4	4	46.57
P49406|RM19_HUMAN	47.41	8	8	4.16E+05	2	2	0
Q16656|NRF1_HUMAN	47.33	7	7	1.10E+07	2	2	0
O94766|B3GA3_HUMAN	47.29	11	11	2.52E+06	3	3	0
O43290|SNUT1_HUMAN	47.24	6	6	2.54E+05	3	3	98.61
Q96KP1|EXOC2_HUMAN	47.16	3	3	6.81E+05	2	2	0
Q96FX7|TRM61_HUMAN	47.14	18	18	9.01E+06	3	3	0
Q92618|ZN516_HUMAN	47.12	3	3	1.72E+06	3	2	0
P27824|CALX_HUMAN	47.05	7	7	6.93E+06	3	3	53.2
Q6ZXV5|TMTC3_HUMAN	46.88	2	2	1.04E+06	1	1	0
P82673|RT35_HUMAN	46.87	15	15	3.28E+06	3	3	25.11
Q14257|RCN2_HUMAN	46.76	10	10	3.01E+06	2	2	0
Q9Y3Y2|CHTOP_HUMAN	46.73	9	9	1.47E+06	2	2	0
P83111|LACTB_HUMAN	46.68	5	5	2.41E+06	2	2	35.34
P29508|SPB3_HUMAN	46.59	5	5	9.85E+05	2	2	0
Q7Z5P9|MUC19_HUMAN	46.5	2	2	4.60E+06	6	6	25.33
P61018|RAB4B_HUMAN	46.47	5	5		1	0	0
Q6IQ22|RAB12_HUMAN	46.47	5	5		1	0	28.25
Q7Z6P3|RAB44_HUMAN	46.47	2	2		1	0	0
Q86YS6|RAB43_HUMAN	46.47	5	5		1	0	0
Q9BZG1|RAB34_HUMAN	46.47	4	4		1	0	0
Q15027|ACAP1_HUMAN	46.46	4	4	2.38E+05	3	1	44.47
Q5VU43|MYOME_HUMAN	46.41	1	1	4.89E+06	2	2	48.31
Q9H5H4|ZN768_HUMAN	46.38	6	6	4.09E+06	3	3	0
Q9NPD3|EXOS4_HUMAN	46.32	11	11	8.84E+05	2	2	0
P04181|OAT_HUMAN	45.93	13	13	2.06E+06	4	4	0
O95573|ACSL3_HUMAN	45.88	7	7	2.69E+06	3	3	0
Q9Y2R4|DDX52_HUMAN	45.78	8	8	1.78E+06	3	3	0

P51617|IRAK1_HUMAN	45.66	5	5	8.08E+05	2	2	0
Q9BYG3|MK67I_HUMAN	45.56	9	9	2.02E+06	2	2	35
Q8IV63|VRK3_HUMAN	45.4	8	8	1.53E+06	3	3	0
Q8NI60|ADCK3_HUMAN	45.35	5	5	2.68E+05	2	2	0
P12036|NFH_HUMAN	45.19	1	1		2	0	37.73
Q96L92|SNX27_HUMAN	45.08	5	5	5.75E+05	2	2	0
O95563|MPC2_HUMAN	45.07	9	9	9.01E+05	1	1	0
O43824|GTPB6_HUMAN	44.99	4	4	4.60E+05	2	2	0
Q9BQ70|TCF25_HUMAN	44.96	4	4	7.85E+05	2	2	20.09
Q2M2I5|K1C24_HUMAN	44.9	7	7	0.00E+00	2	1	38.31
P10619|PPGB_HUMAN	44.88	6	6	8.38E+05	2	2	0
Q86TN4|TRPT1_HUMAN	44.87	6	6	6.09E+05	1	1	0
O14497|ARI1A_HUMAN	44.84	2	2	3.11E+06	3	3	37.16
Q13608|PEX6_HUMAN	44.83	3	3	7.70E+05	3	3	0
P28838|AMPL_HUMAN	44.8	4	4	4.13E+05	2	2	0
tr|A0A0A6YYH1|A0A0A6YYH	44.75	3	3	1.36E+05	1	1	0
Q96JJ7|TMX3_HUMAN	44.71	6	6	9.90E+05	2	2	0
P82930|RT34_HUMAN	44.7	7	7	1.48E+06	2	1	33.16
Q8IWS0|PHF6_HUMAN	44.68	6	6	3.58E+06	2	2	0
tr|M0R2C6|M0R2C6_HUMA	44.64	5	5	5.73E+05	2	2	71.1
Q15428|SF3A2_HUMAN	44.59	4	4	2.04E+06	1	1	0
Q7Z333|SETX_HUMAN	44.55	1	1	4.74E+06	3	1	0
Q969L4|LSM10_HUMAN	44.46	14	14	5.35E+05	1	1	0
P00352|AL1A1_HUMAN	44.44	5	5	5.34E+05	2	1	28.97
Q8IV08|PLD3_HUMAN	44.44	4	4	1.47E+06	2	2	0
Q9UKX3|MYH13_HUMAN	44.42	1	1		1	0	36.17
Q9Y295|DRG1_HUMAN	44.39	6	6	7.02E+04	2	2	47.95
Q68E01|INT3_HUMAN	44.3	2	2	7.51E+05	1	1	0
Q9BYD2|RM09_HUMAN	44.23	6	6	1.88E+05	1	1	0
P28066|PSA5_HUMAN	44.11	16	16	5.24E+06	3	2	0
Q9BXP5|SRRT_HUMAN	44.08	2	2	4.90E+05	2	2	75.67
P49321|NASP_HUMAN	44	4	4	2.68E+06	3	3	0
Q9UL15|BAG5_HUMAN	44	8	8	8.20E+05	2	2	0
Q9BV44|THUM3_HUMAN	43.89	3	3	5.20E+05	1	1	0
Q9BTY7|HGH1_HUMAN	43.78	7	7	1.26E+06	2	2	0
O60568|PLOD3_HUMAN	43.73	4	4	1.15E+06	2	2	0
Q9H6R4|NOL6_HUMAN	43.72	3	3	3.96E+05	3	2	0
P82650|RT22_HUMAN	43.7	10	10	1.21E+06	3	3	0
A6NHT5|HMX3_HUMAN	43.68	15	15	1.51E+06	3	3	49.42
Q13610|PWP1_HUMAN	43.66	4	4	4.02E+06	2	2	0
Q7Z7G8|VP13B_HUMAN	43.66	1	1	1.29E+05	2	2	20.13
Q9Y3D9|RT23_HUMAN	43.61	11	11	1.65E+06	2	2	21.62
Q7L0J3|SV2A_HUMAN	43.44	9	9	1.30E+07	4	4	0
P51398|RT29_HUMAN	43.42	3	3	3.72E+05	1	1	48.83
P46934|NEDD4_HUMAN	43.16	1	1	2.10E+05	2	1	0
O60783|RT14_HUMAN	43.11	22	22	1.84E+06	2	2	0
Q09666|AHNK_HUMAN	43.09	1	1	9.35E+04	3	3	67.82
Q8N1F8|S11IP_HUMAN	42.99	2	2	6.38E+05	2	2	0
Q5HYI8|RABL3_HUMAN	42.94	8	8	1.29E+06	2	2	0
Q5JWF2|GNAS1_HUMAN	42.94	3	3	6.58E+05	3	1	0

Q9NX58|LYAR_HUMAN	42.82	9	9	3.22E+06	2	2	42.55
Q9BYD3|RM04_HUMAN	42.81	15	15	6.77E+05	3	3	0
Q6UX07|DHR13_HUMAN	42.78	5	5	3.10E+05	2	1	0
Q7Z3Y9|K1C26_HUMAN	42.74	2	2		1	0	53.26
P02458|CO2A1_HUMAN	42.7	6	6	1.55E+06	5	5	0
Q13867|BLMH_HUMAN	42.61	7	7	3.39E+06	2	2	0
P62875|RPAB5_HUMAN	42.56	16	16	6.86E+05	1	1	0
P50542|PEX5_HUMAN	42.3	4	4	1.34E+06	2	2	0
P53677|AP3M2_HUMAN	42.27	5	5		2	0	0
P35609|ACTN2_HUMAN	42.24	2	2		2	0	53.8
P42224|STAT1_HUMAN	42.22	3	3	1.21E+06	2	1	0
Q15459|SF3A1_HUMAN	42.17	3	3	4.43E+06	2	2	112.81
Q3L8U1|CHD9_HUMAN	42.11	1	1	7.90E+04	2	1	28.14
Q9UKJ3|GPTC8_HUMAN	42.05	2	2	5.90E+05	2	2	39.68
A6NFI3|ZN316_HUMAN	41.92	2	2	1.53E+05	1	1	0
Q02338|BDH_HUMAN	41.88	7	7	1.70E+06	2	2	0
Q6ZV70|LANC3_HUMAN	41.87	5	5	6.78E+05	1	1	0
Q5JTV8|TOIP1_HUMAN	41.86	2	2	1.07E+06	1	1	0
Q9UJX2|CDC23_HUMAN	41.68	3	3	1.69E+06	2	2	0
Q9UHD8|SEPT9_HUMAN	41.55	3	3	1.15E+06	2	2	0
Q92620|PRP16_HUMAN	41.54	1	1	3.98E+05	1	1	47.89
O75494|SRS10_HUMAN	41.53	15	15	8.76E+06	3	3	79.05
Q96TA2|YMEL1_HUMAN	41.44	6	6	1.27E+06	3	3	0
P98169|ZXDB_HUMAN	41.39	4	4	3.08E+06	2	1	0
Q9HCE6|ARGAL_HUMAN	41.37	3	3	4.63E+05	2	2	0
P42357|HUTH_HUMAN	41.31	3	3	7.42E+05	1	1	0
tr|G3V325|G3V325_HUMAN	41.21	3	3	1.34E+07	2	2	71.62
Q5H9R7|PP6R3_HUMAN	41.02	4	4	2.86E+06	4	4	57.32
Q9BTC8|MTA3_HUMAN	41	6	6	4.57E+05	3	1	40.61
P43357|MAGA3_HUMAN	40.96	4	4	2.92E+05	1	1	0
P82933|RT09_HUMAN	40.91	4	4	9.64E+05	2	2	71.94
P30419|NMT1_HUMAN	40.88	6	6	3.77E+05	2	2	0
P0DN76|U2AF5_HUMAN	40.82	22	22	1.22E+07	3	3	32.07
Q8IWA4|MFN1_HUMAN	40.72	3	3	1.23E+06	2	2	0
Q9UBK9|UXT_HUMAN	40.52	9	9	9.67E+05	2	2	0
Q29RF7|PDS5A_HUMAN	40.48	3	3	7.02E+05	2	2	24.17
O75396|SC22B_HUMAN	40.44	10	10	8.66E+03	2	2	0
P13798|ACPH_HUMAN	40.41	5	5	0.00E+00	2	2	0
P09622|DLDH_HUMAN	40.34	4	4	1.74E+06	2	2	0
Q9C0C9|UBE2O_HUMAN	40.33	1	1	4.67E+05	1	1	0
Q9Y3D0|MIP18_HUMAN	40.33	13	13	2.49E+06	1	1	0
Q13206|DDX10_HUMAN	40.31	3	3	1.63E+06	2	2	0
O94887|FARP2_HUMAN	40.26	2	2	5.22E+05	1	1	44.48
P12814|ACTN1_HUMAN	40.23	2	2		2	0	69.87
O95602|RPA1_HUMAN	40.16	2	2	5.28E+05	2	2	48.87
Q14683|SMC1A_HUMAN	40.08	2	2	5.34E+05	2	1	0
tr|K7ERQ8|K7ERQ8_HUMAN	40.03	11	11	4.19E+05	2	1	0
Q86U06|RBM23_HUMAN	40.02	4	4		1	0	0
Q03518|TAP1_HUMAN	40	3	3	4.86E+05	1	1	0
Q9H9G7|AGO3_HUMAN	40	3	3		2	0	49.98

Q6NY19|KANK3_HUMAN	39.81	4	4	5.11E+06	3	2	23.17
Q08188|TGM3_HUMAN	39.77	4	4	2.74E+05	2	2	0
Q04917|1433F_HUMAN	39.69	8	8	1.95E+05	2	1	0
Q96P11|NSUN5_HUMAN	39.66	6	6	9.91E+05	2	2	33.21
P04049|RAF1_HUMAN	39.61	2	2		1	0	0
Q9UII4|HERC5_HUMAN	39.61	3	3	0.00E+00	2	2	0
P85037|FOXK1_HUMAN	39.57	5	5	2.36E+06	3	3	23.17
P62879|GBB2_HUMAN	39.53	6	6	8.49E+05	1	1	0
Q8IX01|SUGP2_HUMAN	39.51	1	1	3.36E+05	1	1	27.36
Q99996|AKAP9_HUMAN	39.38	1	1	1.09E+05	3	1	63.7
Q969L2|MAL2_HUMAN	39.23	6	6	6.55E+05	1	1	0
Q02224|CENPE_HUMAN	39.12	1	1	1.62E+06	3	1	26.56
Q6PCB5|RSBNL_HUMAN	39.08	4	4	2.52E+06	3	3	37.49
P06241|FYN_HUMAN	39	1	1		1	0	0
P08631|HCK_HUMAN	39	2	2		1	0	0
P11362|FGFR1_HUMAN	39	1	1		1	0	0
P11802|CDK4_HUMAN	39	3	3		1	0	0
P22607|FGFR3_HUMAN	39	1	1		1	0	0
Q07002|CDK18_HUMAN	39	2	2		1	0	0
Q14004|CDK13_HUMAN	39	1	1		1	0	26.04
Q15334|L2GL1_HUMAN	39	1	1	1.97E+05	1	1	0
Q8IZL9|CDK20_HUMAN	39	2	2		1	0	0
Q96Q40|CDK15_HUMAN	39	2	2		1	0	0
Q9NYV4|CDK12_HUMAN	39	1	1		1	0	26.04
Q8WUD4|CCD12_HUMAN	38.94	11	11	2.15E+05	2	1	0
Q7KZF4|SND1_HUMAN	38.87	3	3	9.93E+05	2	2	26.75
Q96TC7|RMD3_HUMAN	38.87	7	7	1.27E+06	2	2	0
O75874|IDHC_HUMAN	38.86	12	12	1.73E+07	4	2	0
Q5TH69|BIG3_HUMAN	38.85	1	1	7.28E+05	2	2	27.8
P51813|BMX_HUMAN	38.79	4	4	1.15E+06	2	2	26.12
Q12770|SCAP_HUMAN	38.55	1	1		1	0	0
Q8WXR4|MYO3B_HUMAN	38.5	1	1		1	0	31.06
Q5VTL8|PR38B_HUMAN	38.4	4	4	3.73E+06	2	2	88.01
O43149|ZZEF1_HUMAN	38.37	0	0		2	0	0
Q8NFQ8|TOIP2_HUMAN	38.37	2	2	4.44E+05	1	1	0
Q8N1T3|MYO1H_HUMAN	38.32	1	1		1	0	48.06
Q9NVC6|MED17_HUMAN	38.32	2	2	3.02E+05	1	1	0
Q92785|REQU_HUMAN	38.24	9	9	1.46E+06	2	2	0
Q76L83|ASXL2_HUMAN	38.2	4	4	8.97E+06	4	4	0
Q13045|FLII_HUMAN	38.08	2	2	1.08E+06	2	2	29.71
Q8NCW6|GLT11_HUMAN	37.94	2	2	4.00E+05	1	1	0
Q9H9P8|L2HDH_HUMAN	37.85	2	2	1.85E+05	1	1	0
Q86XN7|PRSR1_HUMAN	37.8	2	2	1.41E+06	1	1	0
Q15758|AAAT_HUMAN	37.74	4	4	7.78E+06	2	2	0
Q92526|TCPW_HUMAN	37.73	2	2		1	0	35.18
Q9NWB6|ARGL1_HUMAN	37.71	5	5	2.91E+06	2	2	0
A6NC98|CC88B_HUMAN	37.63	1	1	1.67E+06	2	1	0
P35250|RFC2_HUMAN	37.59	7	7	2.99E+06	2	2	0
Q9NYC9|DYH9_HUMAN	37.38	0	0		3	0	0
P00338|LDHA_HUMAN	37.35	6	6	2.21E+06	2	2	0

Q9BZL6|KPCD2_HUMAN	37.31	2	2	2.40E+05	2	1	0
Q9UID3|VPS51_HUMAN	37.27	2	2	2.90E+05	1	1	0
P61604|CH10_HUMAN	37.24	27	27	1.46E+07	2	2	0
Q5U5Q3|MEX3C_HUMAN	37.19	1	1		1	0	57.65
Q6ZN04|MEX3B_HUMAN	37.19	2	2		1	0	50.51
Q86XN8|MEX3D_HUMAN	37.19	1	1		1	0	47.81
O95613|PCNT_HUMAN	37.13	1	1	0.00E+00	2	1	55.58
P28072|PSB6_HUMAN	37.12	5	5	7.25E+05	1	1	0
Q07157|ZO1_HUMAN	37.08	1	1	1.34E+06	1	1	112.43
P62979|RS27A_HUMAN	37.01	12	12	7.16E+06	2	2	27.39
Q9UGI6|KCNN3_HUMAN	36.98	2	2	3.69E+05	1	1	0
tr|E5RI56|E5RI56_HUMAN	36.97	32	32	7.16E+05	1	1	0
Q8TCJ2|STT3B_HUMAN	36.87	2	2	6.15E+05	1	1	26.32
O76013|KRT36_HUMAN	36.83	1	1		1	0	0
Q14532|K1H2_HUMAN	36.83	2	2		1	0	0
Q92764|KRT35_HUMAN	36.83	2	2		1	0	0
P42771|CDN2A_HUMAN	36.71	8	8		1	0	0
P42772|CDN2B_HUMAN	36.71	9	9		1	0	0
Q66GS9|CP135_HUMAN	36.68	1	1		2	0	0
P08134|RHOC_HUMAN	36.67	5	5		1	0	0
P55042|RAD_HUMAN	36.67	3	3		1	0	0
Q9NP97|DLRB1_HUMAN	36.61	17	17	5.97E+05	1	1	0
Q9UHV9|PFD2_HUMAN	36.52	8	8	1.73E+06	1	1	50.88
P11413|G6PD_HUMAN	36.38	5	5	9.03E+05	2	2	0
Q9H8Y5|ANKZ1_HUMAN	36.38	5	5	2.84E+06	2	2	0
Q9NPJ6|MED4_HUMAN	36.37	9	9	1.71E+06	2	2	0
Q9Y2W2|WBP11_HUMAN	36.37	3	3	3.14E+06	2	2	33
P49755|TMEDA_HUMAN	36.34	12	12	1.67E+06	2	2	41.94
O14776|TCRG1_HUMAN	36.27	1	1	1.57E+06	2	2	0
P29597|TYK2_HUMAN	36.16	1	1		1	0	0
P82921|RT21_HUMAN	36.15	16	16	3.38E+05	1	1	0
O75832|PSD10_HUMAN	36.14	9	9	1.88E+06	2	2	0
Q16670|ZSC26_HUMAN	36.03	2	2	1.37E+05	1	1	0
Q9UDY4|DNJB4_HUMAN	35.99	11	11	4.45E+05	2	2	0
P21796|VDAC1_HUMAN	35.94	4	4		1	0	0
Q9H9J2|RM44_HUMAN	35.9	11	11	2.72E+05	2	2	0
P11216|PYGB_HUMAN	35.79	1	1	3.58E+05	1	1	0
Q9UKA9|PTBP2_HUMAN	35.77	2	2		1	0	96.85
Q8IX12|CCAR1_HUMAN	35.74	3	3	4.09E+05	2	2	33.84
Q86V15|CASZ1_HUMAN	35.62	1	1	3.92E+05	1	1	0
P78346|RPP30_HUMAN	35.56	4	4	1.88E+05	1	1	0
P22392|NDKB_HUMAN	35.54	14	14	4.25E+06	2	2	0
Q9BTT0|AN32E_HUMAN	35.51	5	5	1.78E+06	1	1	37.89
Q8WXG9|GPR98_HUMAN	35.47	0	0	0.00E+00	2	1	0
O94788|AL1A2_HUMAN	35.42	2	2		1	0	0
Q02388|CO7A1_HUMAN	35.3	2	2	1.85E+06	4	4	0
O43447|PPIH_HUMAN	35.28	7	7	2.71E+05	1	1	30.93
Q7Z2W4|ZCCHV_HUMAN	35.27	1	1	6.24E+05	1	1	0
Q9Y4G6|TLN2_HUMAN	35.23	1	1	4.07E+00	2	1	45.51
O60244|MED14_HUMAN	35.16	1	1	4.42E+05	1	1	0

P24752|THIL_HUMAN	35.02	9	9	2.84E+06	2	2	79.87
Q96PU5|NED4L_HUMAN	34.96	1	1		1	0	23.5
Q9UPE1|SRPK3_HUMAN	34.88	2	2		1	0	27.2
P31260|HXA10_HUMAN	34.86	3	3	3.60E+05	1	1	0
Q5TZA2|CROCC_HUMAN	34.79	0	0		1	0	25.93
Q92817|EVPL_HUMAN	34.79	0	0		1	0	0
P35222|CTNB1_HUMAN	34.74	1	1		1	0	62.98
Q9NWU2|GID8_HUMAN	34.74	7	7	7.15E+04	1	1	0
P58107|EPIPL_HUMAN	34.71	0	0		1	0	54.95
P25940|CO5A3_HUMAN	34.56	5	5	4.49E+05	4	4	0
Q9ULT8|HECD1_HUMAN	34.55	0	0		2	0	45.57
Q8WXA9|SREK1_HUMAN	34.31	3	3	1.64E+06	1	1	0
Q9ULH0|KDIS_HUMAN	34.24	1	1	5.29E+05	2	2	0
Q6ZU15|SEP14_HUMAN	34.22	7	7	1.76E+05	2	1	21.83
P07195|LDHB_HUMAN	34.17	5	5	1.62E+06	2	2	0
P08243|ASNS_HUMAN	34.14	3	3	1.40E+06	2	2	47.78
P15170|ERF3A_HUMAN	34.14	5	5	6.07E+05	2	1	84.09
Q9Y619|ORNT1_HUMAN	34.06	5	5	1.19E+06	1	1	0
Q86U86|PB1_HUMAN	33.98	1	1	2.89E+05	1	1	41.77
Q9NZR1|TMOD2_HUMAN	33.96	4	4	5.27E+05	1	1	0
P84095|RHOG_HUMAN	33.9	10	10	1.55E+06	1	1	0
P0CG40|SP9_HUMAN	33.87	9	9	5.66E+05	3	3	0
Q08554|DSC1_HUMAN	33.82	3	3	1.50E+06	2	2	33.22
P07305|H10_HUMAN	33.81	4	4	2.59E+06	1	1	46.1
Q13595|TRA2A_HUMAN	33.73	6	6	0.00E+00	2	1	72.48
Q13445|TMED1_HUMAN	33.72	4	4	9.88E+05	1	1	0
P55060|XPO2_HUMAN	33.7	1	1	5.34E+05	2	1	37.44
Q8ND56|LS14A_HUMAN	33.7	8	8	9.22E+06	3	3	75.72
Q9UI09|NDUAC_HUMAN	33.64	21	21	2.42E+06	2	2	0
Q15386|UBE3C_HUMAN	33.59	3	3	4.73E+05	2	1	38.75
Q99873|ANM1_HUMAN	33.54	3	3	4.61E+05	1	1	0
P05026|AT1B1_HUMAN	33.45	4	4	8.20E+05	1	1	0
O43660|PLRG1_HUMAN	33.43	7	7	1.92E+06	2	2	0
O14734|ACOT8_HUMAN	33.37	3	3	2.39E+06	1	1	0
Q9Y4D1|DAAM1_HUMAN	33.34	2	2	2.84E+05	2	1	0
P61626|LYSC_HUMAN	33.23	14	14	3.90E+06	2	2	33.1
Q2QGD7|ZXDC_HUMAN	33.16	2	2		1	0	0
Q8TD26|CHD6_HUMAN	33.08	0	0		1	0	31.82
Q9HCK8|CHD8_HUMAN	33.08	0	0		1	0	0
Q9P2D1|CHD7_HUMAN	33.08	0	0		1	0	0
Q16659|MK06_HUMAN	33.03	2	2		2	0	0
P21266|GSTM3_HUMAN	32.98	8	8	7.42E+05	1	1	0
P07384|CAN1_HUMAN	32.92	2	2	8.04E+05	2	2	0
O14654|IRS4_HUMAN	32.87	3	3	0.00E+00	2	2	0
Q9ULI0|ATD2B_HUMAN	32.85	2	2	2.90E+06	2	2	0
P17152|TMM11_HUMAN	32.81	8	8	1.72E+06	1	1	0
Q86VF2|IGFN1_HUMAN	32.79	2	2	2.62E+06	2	1	0
P19838|NFKB1_HUMAN	32.73	1	1		1	0	29.46
Q9NUA8|ZBT40_HUMAN	32.7	1	1		1	0	21.23
Q9BZF9|UACA_HUMAN	32.62	1	1		2	0	0

O95218|ZRAB2_HUMAN	32.6	3	3	1.11E+06	1	1	34.64
Q8IWJ2|GCC2_HUMAN	32.58	1	1		2	0	0
Q9H8M5|CNNM2_HUMAN	32.48	2	2	1.89E+06	2	1	0
P11498|PYC_HUMAN	32.43	1	1	2.68E+05	1	1	0
Q9H6A9|PCX3_HUMAN	32.43	1	1	4.22E+06	2	1	36.09
P50502|F10A1_HUMAN	32.38	3	3	1.17E+06	1	1	0
Q8IZP2|ST134_HUMAN	32.38	4	4	1.17E+06	1	1	0
Q8NFI4|F10A5_HUMAN	32.38	3	3	1.17E+06	1	1	0
Q03164|KMT2A_HUMAN	32.34	1	1	2.44E+06	2	2	0
Q15648|MED1_HUMAN	32.31	2	2	1.42E+06	2	2	42
Q9NQC8|IFT46_HUMAN	32.12	10	10	1.09E+06	2	2	0
Q15392|DHC24_HUMAN	32.02	4	4	1.20E+06	1	1	0
Q8IUG5|MY18B_HUMAN	31.83	0	0		1	0	0
Q96DT5|DYH11_HUMAN	31.7	0	0		2	0	0
Q8N4C8|MINK1_HUMAN	31.64	3	3	4.07E+05	2	1	26.58
P14406|CX7A2_HUMAN	31.57	12	12	1.39E+06	1	1	0
Q96GA3|LTV1_HUMAN	31.52	4	4	1.07E+06	1	1	21.94
P07951|TPM2_HUMAN	31.48	13	13	5.05E+06	3	3	0
P52435|RPB11_HUMAN	31.47	35	35	7.15E+05	2	2	0
P09211|GSTP1_HUMAN	31.35	8	8	4.55E+06	1	1	42.63
O00585|CCL21_HUMAN	31.34	5	5		1	0	0
Q9H9T3|ELP3_HUMAN	31.32	3	3	6.48E+05	2	2	0
Q5VST9|OBSCN_HUMAN	31.31	1	1	7.08E+05	3	3	25.95
Q02241|KIF23_HUMAN	31.29	1	1		1	0	0
P43362|MAGA9_HUMAN	31.19	9	9	6.98E+06	2	2	0
Q15072|OZF_HUMAN	31.19	4	4	1.85E+05	1	1	0
O43299|AP5Z1_HUMAN	31.13	3	3	0.00E+00	2	1	0
Q96JB1|DYH8_HUMAN	31.11	0	0		2	0	28.9
Q14202|ZMYM3_HUMAN	30.87	1	1	8.38E+05	2	2	0
Q9H9F9|ARP5_HUMAN	30.86	2	2	3.88E+05	1	1	0
Q7Z4W1|DCXR_HUMAN	30.71	16	16	1.15E+06	2	2	0
O15357|SHIP2_HUMAN	30.69	1	1		1	0	0
Q92835|SHIP1_HUMAN	30.69	1	1		1	0	0
P0C091|FREM3_HUMAN	30.67	1	1	7.32E+05	2	1	33.03
Q9Y3R5|DOP2_HUMAN	30.61	1	1	0.00E+00	2	1	0
Q9Y5B6|PAXB1_HUMAN	30.6	1	1	6.40E+04	1	1	31.07
Q8WXD9|CSKI1_HUMAN	30.55	3	3	4.09E+05	2	2	0
Q15274|NADC_HUMAN	30.49	3	3	2.38E+05	1	1	71.25
P15502|ELN_HUMAN	30.45	6	6	1.60E+06	2	2	57.64
O43303|CP110_HUMAN	30.44	2	2	1.57E+06	1	1	30.02
P11117|PPAL_HUMAN	30.43	3	3	2.36E+05	1	1	0
O60732|MAGC1_HUMAN	30.41	1	1	1.52E+05	1	1	0
Q8IWT6|LRC8A_HUMAN	30.31	2	2	1.79E+06	2	1	23.17
P53420|CO4A4_HUMAN	30.26	1	1	2.92E+07	2	2	0
O75317|UBP12_HUMAN	30.24	2	2	2.19E+05	1	1	0
P62068|UBP46_HUMAN	30.24	2	2	2.19E+05	1	1	0
Q9UPY3|DICER_HUMAN	30.2	2	2	5.19E+05	2	2	38.18
Q6NZY4|ZCHC8_HUMAN	30.1	3	3	1.91E+06	1	1	0
Q13243|SRSF5_HUMAN	30.09	9	9	2.44E+06	2	1	49.83
P35080|PROF2_HUMAN	30.08	6	6	3.43E+06	1	1	0

Q9UJ14|GGT7_HUMAN	29.99	5	5	9.83E+05	2	2	0
A8MW92|P20L1_HUMAN	29.97	4	4	1.56E+06	2	2	27.19
Q96T23|RSF1_HUMAN	29.96	1	1	1.34E+05	1	1	0
Q9H497|TOR3A_HUMAN	29.84	4	4	1.65E+05	1	1	0
Q13427|PPIG_HUMAN	29.76	1	1	9.23E+05	1	1	0
Q6DD88|ATLA3_HUMAN	29.68	2	2	7.23E+04	1	1	0
Q7L2J0|MEPCE_HUMAN	29.62	3	3	1.26E+06	2	2	29.24
Q9BZ23|PANK2_HUMAN	29.6	5	5	2.07E+05	2	1	0
Q9Y6J0|CABIN_HUMAN	29.49	1	1	4.03E+06	2	2	0
Q96AE4|FUBP1_HUMAN	29.42	1	1		1	0	44.51
Q96I24|FUBP3_HUMAN	29.42	1	1		1	0	64.67
Q96JQ0|PCD16_HUMAN	29.4	1	1	1.07E+05	2	2	0
Q9P266|JCAD_HUMAN	29.32	2	2	2.04E+06	2	2	0
P15586|GNS_HUMAN	29.29	2	2	4.09E+05	1	1	0
Q9H0U6|RM18_HUMAN	29.04	5	5	5.78E+05	1	1	0
P31323|KAP3_HUMAN	29.02	4	4		1	0	38.86
Q07283|TRHY_HUMAN	29.01	1	1	0.00E+00	2	1	0
A2IDD5|CCD78_HUMAN	28.99	1	1		1	0	0
Q8WXH0|SYNE2_HUMAN	28.99	0	0		1	0	0
Q96EK4|THA11_HUMAN	28.96	3	3	7.88E+05	1	1	0
Q9H7E9|CH033_HUMAN	28.94	7	7	1.11E+06	1	1	0
O75376|NCOR1_HUMAN	28.9	1	1	7.21E+05	2	1	0
Q8WU90|ZC3HF_HUMAN	28.89	2	2	7.69E+05	1	1	0
Q9NX20|RM16_HUMAN	28.76	7	7	8.21E+05	1	1	0
Q9UFH2|DYH17_HUMAN	28.73	1	1	2.68E+05	2	1	0
Q8N7J2|AMER2_HUMAN	28.66	3	3	1.22E+06	2	2	0
Q9HDB9|GAK5_HUMAN	28.6	3	3	1.76E+05	1	1	0
P36776|LONM_HUMAN	28.56	1	1	1.72E+05	1	1	0
P23490|LORI_HUMAN	28.43	4	4	3.13E+06	1	1	0
Q52LW3|RHG29_HUMAN	28.42	1	1		2	0	24.24
P25686|DNJB2_HUMAN	28.36	4	4	4.61E+05	1	1	0
P42345|MTOR_HUMAN	28.35	1	1	2.08E+05	2	1	0
P51843|NR0B1_HUMAN	28.3	3	3	7.91E+05	2	1	0
P04114|APOB_HUMAN	28.28	0	0		1	0	0
Q9Y4F3|MARF1_HUMAN	28.28	0	0		1	0	0
P12277|KCRB_HUMAN	28.23	3	3	4.07E+05	1	1	54.02
Q8IVF4|DYH10_HUMAN	28.17	0	0	1.72E+05	2	1	25.92
Q92905|CSN5_HUMAN	28.15	2	2	4.93E+04	1	1	0
P59047|NALP5_HUMAN	28.14	1	1	5.26E+05	2	1	0
A2A288|ZC12D_HUMAN	27.95	1	1		1	0	0
P25391|LAMA1_HUMAN	27.95	0	0		1	0	0
Q9BYJ9|YTHD1_HUMAN	27.85	3	3	5.30E+05	1	1	0
Q15776|ZKSC8_HUMAN	27.83	2	2	6.98E+05	1	1	0
Q9NZT1|CALL5_HUMAN	27.81	16	16	1.24E+06	1	1	0
Q16822|PCKGM_HUMAN	27.8	2	2	3.63E+05	1	1	0
Q5VTQ0|TT39B_HUMAN	27.78	2	2	2.01E+06	1	1	0
tr|H0Y858|H0Y858_HUMAN	27.78	1	1		1	0	23.04
O75153|CLU_HUMAN	27.76	3	3	1.73E+06	2	2	0
P39880|CUX1_HUMAN	27.73	3	3	3.70E+05	2	2	0
Q9NZJ4|SACS_HUMAN	27.73	1	1	0.00E+00	2	1	0

P29401|TKT_HUMAN	27.66	2	2	6.06E+05	1	1	81.87
P35475|IDUA_HUMAN	27.55	3	3	7.03E+05	1	1	0
Q8TDM6|DLG5_HUMAN	27.55	1	1	1.57E+05	2	2	21.95
Q5VIR6|VPS53_HUMAN	27.53	1	1	3.98E+05	1	1	0
Q8NF91|SYNE1_HUMAN	27.53	0	0	3.46E+06	2	2	0
Q9BV73|CP250_HUMAN	27.51	0	0		1	0	0
Q9BZ76|CNTP3_HUMAN	27.51	0	0		1	0	0
O15061|SYNEM_HUMAN	27.48	0	0		1	0	0
P17661|DESM_HUMAN	27.48	1	1		1	0	30.61
Q8N283|ANR35_HUMAN	27.48	1	1		1	0	0
Q9ULE0|WWC3_HUMAN	27.48	1	1		1	0	0
Q9UM73|ALK_HUMAN	27.48	0	0		1	0	0
Q9Y2D5|AKAP2_HUMAN	27.48	1	1		1	0	0
Q96DV4|RM38_HUMAN	27.42	2	2	4.13E+05	1	1	24.03
Q00973|B4GN1_HUMAN	27.17	1	1		1	0	0
Q4AC94|C2CD3_HUMAN	27.17	0	0		1	0	0
P05423|RPC4_HUMAN	27.13	6	6	3.54E+05	2	2	0
Q14145|KEAP1_HUMAN	27.13	3	3	2.23E+05	1	1	0
Q9H7Z7|PGES2_HUMAN	27.07	5	5	8.93E+03	1	1	0
Q86WT1|TT30A_HUMAN	27	2	2	7.81E+05	1	1	0
Q76FK4|NOL8_HUMAN	26.93	1	1		1	0	0
Q9Y4E5|ZN451_HUMAN	26.9	2	2	2.24E+05	1	1	0
Q6P996|PDXD1_HUMAN	26.88	1	1	4.23E+05	1	1	0
Q9H3N1|TMX1_HUMAN	26.87	4	4	2.37E+05	1	1	0
P56556|NDUA6_HUMAN	26.76	5	5	1.58E+06	1	1	0
P12259|FA5_HUMAN	26.73	0	0	1.25E+06	1	1	30.26
Q86TD4|SRCA_HUMAN	26.73	1	1	1.73E+06	1	1	20.07
Q6DN14|MCTP1_HUMAN	26.65	1	1	1.02E+06	1	1	0
Q92538|GBF1_HUMAN	26.65	0	0		1	0	36.9
O95104|SFR15_HUMAN	26.6	1	1		1	0	35.4
Q9UPN6|SCAF8_HUMAN	26.6	1	1		1	0	0
P02787|TRFE_HUMAN	26.58	2	2	6.48E+05	1	1	0
O15068|MCF2L_HUMAN	26.57	1	1		2	0	0
Q16181|SEPT7_HUMAN	26.56	2	2		1	0	30.41
Q92599|SEPT8_HUMAN	26.56	2	2		1	0	21.83
Q8N1B4|VPS52_HUMAN	26.49	2	2	7.56E+05	1	1	0
Q8WXX5|DNJC9_HUMAN	26.44	5	5	1.02E+06	1	1	100.18
Q9C0A1|ZFHX2_HUMAN	26.4	1	1	1.77E+06	2	2	0
P01860|IGHG3_HUMAN	26.39	6	6	3.72E+04	2	2	0
Q32P44|EMAL3_HUMAN	26.24	2	2	4.84E+06	1	1	0
Q13207|TBX2_HUMAN	26.2	8	8	3.21E+06	2	2	0
Q6NWY9|PR40B_HUMAN	26.17	2	2	6.57E+05	1	1	26.24
O95248|MTMR5_HUMAN	26.15	0	0		1	0	0
Q92878|RAD50_HUMAN	26.11	1	1	2.67E+05	1	1	0
Q9BXS5|AP1M1_HUMAN	26.07	2	2	2.45E+05	1	1	0
Q9Y6Q5|AP1M2_HUMAN	26.07	2	2	2.45E+05	1	1	0
Q15102|PA1B3_HUMAN	26.01	4	4	4.29E+05	1	1	30.75
Q9H6S0|YTDC2_HUMAN	25.91	3	3	1.42E+06	2	2	68.92
Q9Y5K3|PCY1B_HUMAN	25.91	3	3	2.93E+05	1	1	0
Q9H334|FOXP1_HUMAN	25.89	2	2	4.76E+05	1	1	0

Q5VZ46|K1614_HUMAN	25.79	1	1	6.98E+05	1	1	0
Q9BQT8|ODC_HUMAN	25.77	5	5	0.00E+00	1	1	0
Q96S55|WRIP1_HUMAN	25.76	1	1	6.57E+05	1	1	0
Q9NPL8|TIDC1_HUMAN	25.7	5	5	4.82E+05	1	1	0
Q8WU76|SCFD2_HUMAN	25.68	4	4	1.94E+05	2	2	0
P0DMV2|CT459_HUMAN	25.59	5	5	8.51E+05	1	1	0
Q9Y6D5|BIG2_HUMAN	25.52	1	1	1.43E+05	1	1	0
Q5JTH9|RRP12_HUMAN	25.5	1	1	3.96E+05	1	1	0
Q96Q15|SMG1_HUMAN	25.49	0	0		2	0	21.25
P23352|KALM_HUMAN	25.48	3	3	3.38E+06	2	2	0
Q01955|CO4A3_HUMAN	25.48	3	3	3.04E+04	2	2	0
P43361|MAGA8_HUMAN	25.43	4	4	8.84E+06	1	1	0
Q5VYK3|ECM29_HUMAN	25.43	0	0		1	0	0
Q63HN8|RN213_HUMAN	25.42	1	1	2.46E+04	2	2	21.71
Q7Z494|NPHP3_HUMAN	25.36	3	3	0.00E+00	2	2	0
Q9Y4H2|IRS2_HUMAN	25.3	1	1	1.35E+06	1	1	0
Q03828|EVX2_HUMAN	25.29	10	10	3.85E+06	2	2	0
Q14566|MCM6_HUMAN	25.24	1	1	2.37E+05	1	1	44.42
Q02750|MP2K1_HUMAN	25.23	3	3		1	0	0
tr|A0A1B0GUL7|A0A1B0GUL	25.23	3	3		1	0	0
Q13617|CUL2_HUMAN	25.22	1	1		1	0	0
Q9BYD6|RM01_HUMAN	25.17	3	3	5.99E+05	2	2	0
Q9NZW5|MPP6_HUMAN	25.17	2	2	4.39E+05	1	1	0
Q9NQ92|COPRS_HUMAN	25.14	14	14	2.13E+07	2	2	42.96
O14917|PCD17_HUMAN	25.02	2	2	2.41E+05	1	1	0
Q9UHR5|S30BP_HUMAN	25	4	4	7.27E+05	1	1	0
Q9P0M6|H2AW_HUMAN	24.97	5	5		1	0	23.76
Q9HBR0|S38AA_HUMAN	24.87	1	1	4.18E+04	1	1	0
Q9NRR4|RNC_HUMAN	24.85	1	1	0.00E+00	1	1	0
Q14667|K0100_HUMAN	24.84	1	1	6.72E+07	2	1	0
Q9H4A3|WNK1_HUMAN	24.83	1	1	9.41E+05	1	1	21.29
Q8WVM0|TFB1M_HUMAN	24.81	5	5	1.19E+06	1	1	0
Q92545|TM131_HUMAN	24.71	1	1	0.00E+00	1	1	0
Q8N3D4|EH1L1_HUMAN	24.65	1	1	0.00E+00	1	1	26.97
Q6DN03|H2B2C_HUMAN	24.64	5	5		2	0	20.05
Q6DRA6|H2B2D_HUMAN	24.64	6	6		2	0	0
Q8TDX9|PK1L1_HUMAN	24.64	0	0		1	0	34.08
A6NNT2|CP096_HUMAN	24.61	1	1		1	0	0
Q07864|DPOE1_HUMAN	24.58	1	1	7.68E+05	2	2	0
Q15772|SPEG_HUMAN	24.58	1	1	7.25E+07	2	2	0
O43772|MCAT_HUMAN	24.48	3	3	2.11E+06	1	1	0
Q9UJC3|HOOK1_HUMAN	24.48	1	1	3.90E+05	1	1	0
Q9UPT5|EXOC7_HUMAN	24.44	5	5	8.48E+05	1	1	0
tr|D6RIA3|D6RIA3_HUMAN	24.44	3	3	1.65E+05	2	2	0
Q2WGJ9|FR1L6_HUMAN	24.3	1	1	4.64E+03	1	1	0
Q9NX04|CA109_HUMAN	24.28	4	4	7.14E+05	1	1	0
Q9NR82|KCNQ5_HUMAN	24.25	2	2	5.11E+04	1	1	0
Q15475|SIX1_HUMAN	24.22	4	4	9.88E+04	1	1	0
Q6NUI6|CHADL_HUMAN	24.14	2	2	1.36E+06	1	1	0
O75445|USH2A_HUMAN	24.03	0	0		1	0	0

Q86YV9|HPS6_HUMAN	24.03	3	3	1.99E+05	1	1	0
Q8N5N7|RM50_HUMAN	23.86	12	12	0.00E+00	1	1	0
P62891|RL39_HUMAN	23.85	20	20	7.69E+06	1	1	0
Q59GN2|R39L5_HUMAN	23.85	20	20	7.69E+06	1	1	0
O00408|PDE2A_HUMAN	23.7	1	1		1	0	0
O75052|CAPON_HUMAN	23.63	7	7	2.27E+03	2	1	0
Q9NVH0|EXD2_HUMAN	23.56	2	2	4.13E+05	1	1	35.28
Q9UL03|INT6_HUMAN	23.56	2	2	6.96E+05	2	2	0
O14523|C2C2L_HUMAN	23.55	1	1		1	0	0
O60673|DPOLZ_HUMAN	23.55	0	0		1	0	0
P46939|UTRO_HUMAN	23.55	0	0		1	0	0
Q5JV73|FRPD3_HUMAN	23.55	0	0		1	0	0
Q7Z3V4|UBE3B_HUMAN	23.55	1	1		1	0	0
Q8WYP5|ELYS_HUMAN	23.55	0	0		1	0	0
Q9NRU3|CNNM1_HUMAN	23.55	1	1		1	0	0
Q9Y6B7|AP4B1_HUMAN	23.55	1	1		1	0	0
Q14957|NMDE3_HUMAN	23.52	0	0		1	0	0
Q8WUM4|PDC6I_HUMAN	23.5	1	1		1	0	0
Q5THJ4|VP13D_HUMAN	23.48	0	0		1	0	0
O15417|TNC18_HUMAN	23.46	1	1	2.58E+05	2	2	29.34
P09001|RM03_HUMAN	23.43	2	2	2.30E+05	1	1	0
O60512|B4GT3_HUMAN	23.29	3	3	1.44E+05	1	1	0
P02666|CASB_BOVIN	23.23	8	8	6.32E+05	1	1	36.96
Q9NZL4|HPBP1_HUMAN	23.17	3	3	1.74E+05	1	1	0
Q8IY26|PLPP6_HUMAN	23.12	3	3	5.00E+05	1	1	0
Q6ZQQ6|WDR87_HUMAN	23.1	1	1	1.54E+05	1	1	0
Q15735|PI5PA_HUMAN	23.07	3	3	0.00E+00	1	1	0
Q6P2D8|XRRA1_HUMAN	23.07	1	1		1	0	29.32
Q8N201|INT1_HUMAN	22.99	0	0		1	0	0
P12111|CO6A3_HUMAN	22.98	1	1	5.43E+05	1	1	0
Q15843|NEDD8_HUMAN	22.98	17	17	2.96E+05	1	1	0
Q9H0U9|TSYL1_HUMAN	22.91	3	3	1.43E+06	1	1	0
Q8NFJ8|BHE22_HUMAN	22.9	2	2		1	0	24.01
Q96EK2|PF21B_HUMAN	22.86	5	5	1.28E+05	1	1	0
Q92611|EDEM1_HUMAN	22.77	1	1		1	0	24.99
O15235|RT12_HUMAN	22.76	9	9	3.30E+05	1	1	0
Q7KZN9|COX15_HUMAN	22.72	2	2	4.07E+05	1	1	0
Q9H2D6|TARA_HUMAN	22.6	1	1	3.59E+05	1	1	0
Q14684|RRP1B_HUMAN	22.59	2	2	2.17E+05	1	1	20.99
Q8TE04|PANK1_HUMAN	22.59	2	2	1.62E+06	1	1	0
Q04637|IF4G1_HUMAN	22.57	1	1	3.44E+05	1	1	111.68
Q96EL2|RT24_HUMAN	22.55	5	5	8.98E+05	1	1	20.63
Q9NYQ6|CELR1_HUMAN	22.54	0	0		1	0	26.3
P24666|PPAC_HUMAN	22.51	6	6	3.92E+05	1	1	0
Q5VZ18|SHE_HUMAN	22.49	3	3	1.22E+06	1	1	0
P27361|MK03_HUMAN	22.46	3	3		1	0	0
P28482|MK01_HUMAN	22.46	3	3		1	0	0
P19823|ITIH2_HUMAN	22.45	2	2	2.09E+05	1	1	0
Q96BI1|S22AI_HUMAN	22.45	3	3	5.39E+05	1	1	0
Q8IZ52|CHSS2_HUMAN	22.35	2	2	3.07E+05	1	1	0

Q13671|RIN1_HUMAN	22.21	1	1		1	0	0
P54829|PTN5_HUMAN	22.15	1	1		1	0	0
Q12923|PTN13_HUMAN	22.15	0	0		1	0	0
O95996|APC2_HUMAN	22.14	0	0		1	0	23.17
Q13751|LAMB3_HUMAN	22.14	1	1		1	0	0
Q6ZRS2|SRCAP_HUMAN	22.14	0	0		1	0	32.4
Q8IYE0|CC146_HUMAN	22.14	1	1		1	0	23.17
Q96T76|MMS19_HUMAN	22.14	1	1	1.03E+06	1	1	0
Q63ZE4|S22AA_HUMAN	22.12	1	1	2.01E+05	1	1	0
Q92766|RREB1_HUMAN	22.09	1	1	1.32E+06	1	1	0
Q10469|MGAT2_HUMAN	22.08	2	2	4.80E+05	1	1	0
O75970|MPDZ_HUMAN	22.05	1	1	3.90E+05	1	1	0
O94762|RECQ5_HUMAN	22.03	1	1	1.22E+06	1	1	0
Q9UMN6|KMT2B_HUMAN	22	1	1	0.00E+00	1	1	0
Q8N841|TTLL6_HUMAN	21.98	1	1		1	0	0
Q8NEN0|ARMC2_HUMAN	21.98	1	1		1	0	0
O43150|ASAP2_HUMAN	21.93	1	1		1	0	0
Q9ULH1|ASAP1_HUMAN	21.93	1	1		1	0	0
Q93100|KPBB_HUMAN	21.89	1	1		1	0	0
Q01826|SATB1_HUMAN	21.85	1	1	4.39E+05	1	1	61.43
Q9NZJ5|E2AK3_HUMAN	21.78	1	1	6.06E+05	1	1	0
O14737|PDCD5_HUMAN	21.75	5	5		1	0	0
O15083|ERC2_HUMAN	21.75	1	1		1	0	0
O75691|UTP20_HUMAN	21.75	0	0		1	0	0
P56715|RP1_HUMAN	21.75	0	0		1	0	0
Q14746|COG2_HUMAN	21.75	1	1		1	0	0
Q2M389|WASH7_HUMAN	21.75	1	1		1	0	0
Q7Z2Y8|GVIN1_HUMAN	21.75	0	0		1	0	0
Q7Z460|CLAP1_HUMAN	21.75	0	0		1	0	26.86
Q96JJ3|ELMO2_HUMAN	21.75	1	1		1	0	0
Q9Y5Y2|NUBP2_HUMAN	21.71	3	3	1.70E+06	1	1	0
Q9C0H9|SRCN1_HUMAN	21.65	2	2	0.00E+00	1	1	0
P22234|PUR6_HUMAN	21.63	2	2		1	0	41.82
P61020|RAB5B_HUMAN	21.59	5	5		1	0	0
Q9UGP8|SEC63_HUMAN	21.59	2	2	9.56E+05	1	1	0
O60610|DIAP1_HUMAN	21.56	0	0		1	0	0
Q02846|GUC2D_HUMAN	21.56	1	1		1	0	0
Q96JM2|ZN462_HUMAN	21.56	0	0		1	0	0
Q9BZC7|ABCA2_HUMAN	21.56	0	0		1	0	0
Q9UBN4|TRPC4_HUMAN	21.56	1	1		1	0	0
Q9UPP1|PHF8_HUMAN	21.56	1	1		1	0	0
Q96A72|MGN2_HUMAN	21.52	11	11	2.00E+06	1	1	0
O95870|ABHGA_HUMAN	21.49	2	2	1.66E+05	1	1	0
Q15751|HERC1_HUMAN	21.44	0	0	9.57E+05	1	1	0
P30085|KCY_HUMAN	21.41	7	7	5.65E+04	1	1	0
P49591|SYSC_HUMAN	21.41	2	2	3.20E+05	1	1	54.38
Q15075|EEA1_HUMAN	21.4	0	0		1	0	0
Q8NDI1|EHBP1_HUMAN	21.4	0	0		1	0	0
Q8TB24|RIN3_HUMAN	21.4	1	1		1	0	0
Q96PE1|AGRA2_HUMAN	21.4	0	0		1	0	0

Q99698|LYST_HUMAN	21.4	0	0		1	0	0
P13667|PDIA4_HUMAN	21.36	2	2	2.71E+05	1	1	47.52
Q8TD57|DYH3_HUMAN	21.31	0	0		1	0	0
Q9P225|DYH2_HUMAN	21.31	0	0		1	0	0
A6NGR9|MROH6_HUMAN	21.3	1	1		1	0	0
Q9ULX3|NOB1_HUMAN	21.28	4	4	2.59E+05	1	1	0
Q13185|CBX3_HUMAN	21.27	8	8	1.27E+06	1	1	0
O75417|DPOLQ_HUMAN	21.21	0	0		1	0	28.84
Q8NI77|KI18A_HUMAN	21.21	1	1		1	0	0
P49756|RBM25_HUMAN	21.16	1	1	9.24E+05	1	1	0
Q13043|STK4_HUMAN	21.16	3	3	1.16E+06	1	1	0
Q92918|M4K1_HUMAN	21.06	2	2	6.07E+04	1	1	0
Q9NS87|KIF15_HUMAN	21.06	0	0		1	0	0
Q9Y388|RBMX2_HUMAN	21.06	6	6	2.40E+05	1	1	0
Q8IY37|DHX37_HUMAN	20.99	1	1	1.19E+05	1	1	30.74
Q8IY81|SPB1_HUMAN	20.92	1	1	6.02E+04	1	1	0
Q8NFC6|BD1L1_HUMAN	20.84	1	1	0.00E+00	1	1	0
Q8WY21|SORC1_HUMAN	20.78	1	1	1.11E+06	1	1	0
Q5JPB2|ZN831_HUMAN	20.71	1	1	1.32E+04	1	1	27.4
Q9BTC0|DIDO1_HUMAN	20.66	1	1	3.59E+05	1	1	0
Q9BXU7|UBP26_HUMAN	20.64	1	1		1	0	24.24
Q9Y4R8|TELO2_HUMAN	20.61	2	2	2.34E+05	1	1	0
Q14669|TRIPC_HUMAN	20.57	0	0		1	0	0
O95819|M4K4_HUMAN	20.52	1	1		1	0	0
P0C7X1|TBC3H_HUMAN	20.51	1	1	4.06E+05	1	1	0
P35125|UBP6_HUMAN	20.51	0	0	4.06E+05	1	1	0
P49815|TSC2_HUMAN	20.51	0	0		1	0	35.32
Q9UP95|S12A4_HUMAN	20.48	1	1		1	0	0
Q9H5Q4|TFB2M_HUMAN	20.47	5	5	6.14E+05	1	1	0
O43688|PLPP2_HUMAN	20.44	2	2		1	0	0
Q96PV0|SYGP1_HUMAN	20.41	1	1	0.00E+00	1	1	0
Q96L91|EP400_HUMAN	20.39	0	0	1.45E+05	1	1	0
Q8TF21|ANR24_HUMAN	20.34	3	3	0.00E+00	1	1	0
A6NHR9|SMHD1_HUMAN	20.33	0	0	2.27E+05	1	1	115.14
Q14008|CKAP5_HUMAN	20.33	1	1	4.58E+05	1	1	33.68
Q969V3|NCLN_HUMAN	20.33	2	2	2.48E+05	1	1	0
P98171|RHG04_HUMAN	20.3	1	1	1.20E+05	1	1	0
Q8WVC6|DCAKD_HUMAN	20.25	4	4	7.90E+04	1	1	0
P08240|SRPRA_HUMAN	20.23	4	4	8.58E+05	1	1	0
Q8IYQ7|THNS1_HUMAN	20.2	1	1		1	0	0
P38432|COIL_HUMAN	20.12	2	2	0.00E+00	1	1	0
Q9Y697|NFS1_HUMAN	20.08	4	4	0.00E+00	1	1	0
Q96G03|PGM2_HUMAN	20.07	1	1		1	0	0
Q9Y5E9|PCDBE_HUMAN	20.05	2	2	2.54E+06	1	1	0
Q9Y5F3|PCDB1_HUMAN	20.05	2	2	2.54E+06	1	1	0
Q562E7|WDR81_HUMAN	20.01	1	1	1.96E+06	1	1	0

Supplementary Table 2: RCOR1 Mass spectrometry							
Accession	-10lgP	Coverage (%)	Coverage (%) Sampl	Area Sample 2	#Peptides	#Unique	scores in background
O60341|KDM1A_HUMAN	409.44	90	90	6.13E+09	149	149	0
Q9UKL0|RCOR1_HUMAN	364.91	88	88	4.17E+09	96	82	0
Q9UBW7|ZMYM2_HUMAN	363.8	64	64	1.46E+09	90	90	0
O95071|UBR5_HUMAN	387.06	50	50	4.77E+08	121	121	58.61
Q14687|GSE1_HUMAN	316.56	48	48	9.35E+08	69	69	0
Q92769|HDAC2_HUMAN	298.5	71	71	1.29E+09	54	27	0
Q13547|HDAC1_HUMAN	288.98	65	65	3.20E+08	49	22	0
Q9P2K3|RCOR3_HUMAN	225.66	44	44	3.05E+07	27	12	0
Q96AV8|E2F7_HUMAN	295.39	55	55	4.89E+10	89	87	80.13
Q96BD5|PF21A_HUMAN	202.99	26	26	3.41E+07	13	13	0
Q9NP66|HM20A_HUMAN	187.69	40	40	1.06E+08	13	13	0
O75362|ZN217_HUMAN	186.11	30	30	3.15E+07	26	25	0
Q14202|ZMYM3_HUMAN	180.13	13	13	1.87E+07	14	13	0
P63167|DYL1_HUMAN	174.43	78	78	4.05E+07	11	4	0
Q92618|ZN516_HUMAN	170.41	16	16	1.02E+07	14	13	0
P23246|SFPQ_HUMAN	238.78	50	50	8.96E+07	30	29	69.37
Q9P258|RCC2_HUMAN	163.1	26	26	9.16E+06	11	11	0
Q9P0W2|HM20B_HUMAN	160.92	32	32	4.35E+07	13	13	0
P06748|NPM_HUMAN	149.49	29	29	2.28E+07	6	6	0
Q13363|CTBP1_HUMAN	148.14	22	22	7.84E+06	9	8	0
Q8NC51|PAIRB_HUMAN	197.42	40	40	1.73E+08	39	39	56.15
Q8IZ40|RCOR2_HUMAN	139.78	15	15	6.89E+07	9	4	0
P32969|RL9_HUMAN	135.88	29	29	3.53E+06	3	3	0
Q96FJ2|DYL2_HUMAN	168.07	53	53	6.25E+06	10	3	35.17
Q15233|NONO_HUMAN	131.99	28	28	1.13E+07	11	10	0
P10412|H14_HUMAN	131.67	16	16		5	0	0
P16402|H13_HUMAN	131.67	15	15		5	0	0
P08865|RSSA_HUMAN	202.26	58	58	6.75E+07	14	14	70.8
P0C0S8|H2A1_HUMAN	122.25	35	35	2.20E+05	4	1	0
P20671|H2A1D_HUMAN	122.25	35	35	2.20E+05	4	1	0
Q96KK5|H2A1H_HUMAN	122.25	36	36	2.20E+05	4	1	0
Q99878|H2A1J_HUMAN	122.25	36	36	2.20E+05	4	1	0
tr|A0A0U1RRH7|A0A0U1RR	122.25	27	27	2.20E+05	4	1	0
P12532|KCRU_HUMAN	121.27	19	19	6.71E+06	5	5	0
Q13263|TIF1B_HUMAN	203.48	40	40	7.86E+07	37	37	85.02
Q9C0C7|AMRA1_HUMAN	116.27	8	8	3.16E+06	6	6	0
O43390|HNRPR_HUMAN	113.05	11	11	1.19E+06	5	2	0
tr|A0A075B6R1|A0A075B6R	112.83	15	15		3	0	0
tr|A0A075B6S6|A0A075B6S	112.83	13	13		3	0	0
P62987|RL40_HUMAN	111.24	32	32	2.23E+07	4	3	0
Q96EK2|PF21B_HUMAN	111.08	17	17	3.42E+06	5	5	0
Q2VIR3|IF2GL_HUMAN	101.97	7	7	2.25E+06	3	3	0
O95714|HERC2_HUMAN	101.17	2	2	5.03E+06	7	7	0
P52272|HNRPM_HUMAN	98.42	11	11	1.49E+06	5	5	0
Q5VTD9|GFI1B_HUMAN	98.12	13	13	8.69E+06	4	3	0
Q14011|CIRBP_HUMAN	95.48	19	19	3.35E+06	2	2	0

P55795|HNRH2_HUMAN	93.11	9	9	1.42E+05	4	1	0
Q14444|CAPR1_HUMAN	92.77	4	4	3.02E+06	3	3	0
P63244|RACK1_HUMAN	165.42	57	57	2.00E+07	12	12	82.73
Q12905|ILF2_HUMAN	121.17	23	23	6.47E+06	6	6	40.09
Q92766|RREB1_HUMAN	80.63	2	2	3.21E+06	2	2	0
Q9UKL3|C8AP2_HUMAN	79.01	2	2	5.23E+06	4	4	0
Q5VZL5|ZMYM4_HUMAN	78.73	3	3	1.19E+07	4	3	0
Q02978|M2OM_HUMAN	78.65	11	11	8.83E+05	3	3	0
Q96CT7|CC124_HUMAN	77.5	16	16	3.51E+06	2	2	0
Q00688|FKBP3_HUMAN	76.74	10	10	1.23E+06	2	2	0
P63220|RS21_HUMAN	76.62	35	35	1.97E+06	2	2	0
P16401|H15_HUMAN	97.43	15	15	1.66E+06	3	3	21
Q9H2U1|DHX36_HUMAN	75.99	6	6	9.89E+05	3	3	0
Q01538|MYT1_HUMAN	74.78	7	7	2.86E+06	4	3	0
tr|A0A0G2JS52|A0A0G2JS52	74.78	9	9	2.86E+06	4	3	0
Q9H0C2|ADT4_HUMAN	73.16	13	13	5.08E+05	3	1	0
P52292|IMA1_HUMAN	72	6	6	1.77E+06	2	2	0
Q9UQ80|PA2G4_HUMAN	71.96	10	10	1.23E+06	3	3	0
Q14498|RBM39_HUMAN	110.7	15	15	3.84E+06	6	6	42.21
O60762|DPM1_HUMAN	67.6	19	19	1.71E+06	3	3	0
P62829|RL23_HUMAN	99.48	20	20	5.60E+06	2	2	32.14
Q13283|G3BP1_HUMAN	66.78	4	4	1.26E+06	1	1	0
P18077|RL35A_HUMAN	63.88	15	15	3.76E+06	3	3	0
P35637|FUS_HUMAN	63.14	6	6	1.59E+06	2	2	0
P31944|CASPE_HUMAN	83.95	17	17	1.36E+06	4	4	21.61
P84103|SRSF3_HUMAN	62.24	13	13	7.13E+06	2	1	0
P52732|KIF11_HUMAN	280.65	53	53	1.99E+08	50	50	220.21
P17858|PFKAL_HUMAN	124.93	8	8	5.23E+05	4	1	65.05
P46777|RL5_HUMAN	181.37	43	43	4.80E+07	13	13	122.84
O15379|HDAC3_HUMAN	58.4	4	4		3	0	0
Q08380|LG3BP_HUMAN	58.38	5	5	1.26E+06	2	2	0
Q7L1I2|SV2B_HUMAN	89.78	6	6	1.35E+06	3	3	32.34
O15075|DCLK1_HUMAN	81.36	6	6	5.09E+05	3	3	24.45
Q16658|FSCN1_HUMAN	56.9	5	5	4.69E+05	2	2	0
P51571|SSRD_HUMAN	55.38	17	17	1.54E+06	2	2	0
Q9UJU5|FOXD3_HUMAN	54.67	21	21	4.36E+06	5	5	0
P16403|H12_HUMAN	137.68	23	23	1.81E+06	6	1	83.8
P62244|RS15A_HUMAN	53.48	22	22	2.34E+06	2	2	0
P34932|HSP74_HUMAN	122.63	11	11	1.91E+06	5	4	69.99
P50402|EMD_HUMAN	51.61	5	5	8.24E+05	1	1	0
O15020|SPTN2_HUMAN	111.48	2	2	1.40E+05	5	1	60.42
Q9NR30|DDX21_HUMAN	169.32	15	15	8.45E+06	10	9	119.92
Q9NQH7|XPP3_HUMAN	82.82	11	11	1.73E+06	3	2	33.44
P78559|MAP1A_HUMAN	48.86	1	1	1.50E+06	3	2	0
B2RPK0|HGB1A_HUMAN	48.1	7	7	4.48E+05	1	1	0
Q99684|GFI1_HUMAN	47.84	9	9	9.32E+04	2	1	0
Q07157|ZO1_HUMAN	47.79	1	1	3.11E+05	1	1	0
P46779|RL28_HUMAN	47.7	20	20	8.61E+05	3	3	0
Q8NB90|SPAT5_HUMAN	47.38	3	3	2.73E+05	2	2	0
Q3ZCQ8|TIM50_HUMAN	102.87	12	12	4.56E+06	3	3	55.9

Q16795|NDUA9_HUMAN	46.75	5	5	3.00E+05	1	1	0
Q5W0B1|RN219_HUMAN	89.58	8	8	4.78E+06	4	4	43.41
Q16676|FOXD1_HUMAN	46.05	7	7	1.33E+06	3	3	0
Q8NEV1|CSK23_HUMAN	45.8	9	9	1.22E+06	2	2	0
O95816|BAG2_HUMAN	44.38	5	5	1.15E+06	1	1	0
Q96P63|SPB12_HUMAN	64.19	7	7	9.30E+05	2	2	20.47
P11277|SPTB1_HUMAN	42.9	0	0		1	0	0
P20290|BTF3_HUMAN	42.7	9	9	8.00E+05	1	1	0
Q14532|K1H2_HUMAN	42.46	2	2		1	0	0
Q92764|KRT35_HUMAN	42.46	2	2		1	0	0
P51991|ROA3_HUMAN	128.17	23	23	8.27E+06	8	8	85.8
P05387|RLA2_HUMAN	217.97	96	96	5.95E+07	22	22	176.93
Q99497|PARK7_HUMAN	40.96	14	14	5.86E+05	1	1	0
P43243|MATR3_HUMAN	40.81	4	4	4.76E+05	2	2	0
Q8WXI7|MUC16_HUMAN	40.11	1	1	6.18E+06	5	5	0
P0C2W1|FBSP1_HUMAN	39.82	3	3	2.91E+05	1	1	0
Q9HD67|MYO10_HUMAN	39.61	1	1	5.03E+05	1	1	0
P78406|RAE1L_HUMAN	38.8	4	4	6.18E+05	1	1	0
Q04837|SSBP_HUMAN	38.42	18	18	5.75E+05	2	2	0
O00483|NDUA4_HUMAN	38.33	27	27	9.57E+05	2	2	0
Q08170|SRSF4_HUMAN	38.09	2	2	1.47E+06	1	1	0
P62891|RL39_HUMAN	37.96	20	20	1.73E+06	1	1	0
Q59GN2|R39L5_HUMAN	37.96	20	20	1.73E+06	1	1	0
Q5T9A4|ATD3B_HUMAN	37.88	3	3	5.00E+05	2	2	0
Q9NVI7|ATD3A_HUMAN	37.88	3	3	5.00E+05	2	2	0
E9PAV3|NACAM_HUMAN	171.47	8	8	3.67E+07	9	9	133.59
P53999|TCP4_HUMAN	68.68	26	26	6.53E+06	4	4	30.89
Q99575|POP1_HUMAN	37.37	1	1	4.11E+05	1	1	0
Q9Y5V3|MAGD1_HUMAN	36.82	2	2	1.06E+06	2	2	0
Q14574|DSC3_HUMAN	36.81	2	2	1.36E+05	1	1	0
P11021|GRP78_HUMAN	237.22	51	51	7.58E+07	32	29	200.59
Q8NFD5|ARI1B_HUMAN	36.59	3	3	5.42E+05	3	3	0
Q9Y2L5|TPPC8_HUMAN	36.55	0	0		1	0	0
P38646|GRP75_HUMAN	199.24	42	42	9.20E+07	27	25	162.78
Q5TF21|SOGA3_HUMAN	36.29	4	4	8.16E+05	2	2	0
A6NHQ2|FBLL1_HUMAN	36.02	11	11	0.00E+00	2	1	0
P17844|DDX5_HUMAN	69.07	4	4	1.89E+05	2	1	33.35
Q9C0D5|TANC1_HUMAN	35.46	2	2	5.86E+05	2	2	0
Q8NF91|SYNE1_HUMAN	35.19	1	1	2.60E+06	4	4	0
Q9NPD3|EXOS4_HUMAN	35.09	6	6	1.60E+05	1	1	0
P09874|PARP1_HUMAN	35.05	1	1	2.89E+04	1	1	0
O00442|RTCA_HUMAN	34.68	3	3	1.05E+05	1	1	0
Q13610|PWP1_HUMAN	34.19	3	3	3.17E+05	1	1	0
P01040|CYTA_HUMAN	175.42	85	85	4.95E+07	10	9	141.25
P61254|RL26_HUMAN	90.9	27	27	1.03E+07	5	5	56.79
Q9UNX3|RL26L_HUMAN	90.9	27	27	1.03E+07	5	5	56.79
P18085|ARF4_HUMAN	33.58	6	6	6.06E+04	1	1	0
Q9NZT1|CALL5_HUMAN	211.84	71	71	8.52E+07	10	10	178.41
Q14573|ITPR3_HUMAN	32.96	0	0		1	0	0
O00570|SOX1_HUMAN	32.86	11	11	5.06E+06	3	3	0

P09651|ROA1_HUMAN	117.15	24	24	1.16E+06	7	2	84.33
O75129|ASTN2_HUMAN	32.81	3	3	1.69E+07	2	2	0
Q32P51|RA1L2_HUMAN	106	19	19		5	0	73.63
Q9Y6J0|CABIN_HUMAN	32.3	2	2	1.98E+05	2	2	0
P22105|TENX_HUMAN	32.26	1	1	2.77E+06	2	2	0
Q12802|AKP13_HUMAN	32.22	2	2	2.60E+05	2	2	0
Q14644|RASA3_HUMAN	31.88	3	3	1.24E+06	1	1	0
Q8N3K9|CMYA5_HUMAN	31.79	1	1	8.23E+04	2	2	0
Q7Z2W4|ZCCHV_HUMAN	31.75	2	2	1.92E+05	1	1	0
P08572|CO4A2_HUMAN	31.57	2	2	2.30E+07	2	2	0
Q9GZS3|WDR61_HUMAN	31.44	7	7	0.00E+00	1	1	0
O75531|BAF_HUMAN	83.53	40	40	8.39E+06	2	2	52.23
Q6P4R8|NFRKB_HUMAN	31.28	2	2	3.02E+05	2	2	0
Q8WVV9|HNRLL_HUMAN	31.04	4	4	3.26E+06	1	1	0
Q15029|U5S1_HUMAN	64.03	6	6	1.63E+06	4	4	33.19
O00358|FOXE1_HUMAN	30.47	6	6	3.59E+05	2	2	0
Q66PJ3|AR6P4_HUMAN	97.59	22	22	2.74E+06	6	6	67.22
P20849|CO9A1_HUMAN	30.31	5	5	9.74E+05	3	3	0
Q8N3D4|EH1L1_HUMAN	30.14	2	2	8.93E+04	2	2	0
P62857|RS28_HUMAN	70.73	30	30	8.82E+06	2	2	40.73
O75602|SPAG6_HUMAN	29.93	2	2	4.14E+05	1	1	0
Q99250|SCN2A_HUMAN	29.87	1	1	2.20E+05	2	2	0
Q5BJE1|CC178_HUMAN	29.86	3	3	1.85E+06	2	2	0
Q99856|ARI3A_HUMAN	29.84	4	4	2.94E+06	2	2	0
Q00839|HNRPU_HUMAN	131.51	11	11	5.24E+06	8	8	101.7
Q92598|HS105_HUMAN	182.42	25	25	1.92E+07	13	11	152.75
Q7Z478|DHX29_HUMAN	29.5	1	1	4.05E+05	2	2	0
Q9ULD9|ZN608_HUMAN	29.49	1	1	1.57E+06	1	1	0
Q9UHC7|MKRN1_HUMAN	29.47	9	9	2.92E+05	2	2	0
P67936|TPM4_HUMAN	29.38	4	4	2.68E+05	1	1	0
Q96PZ7|CSMD1_HUMAN	29.36	0	0	2.24E+05	1	1	0
O15234|CASC3_HUMAN	29.1	3	3	1.87E+05	1	1	0
P31689|DNJA1_HUMAN	99.64	10	10	4.13E+06	2	2	70.56
Q9P202|WHRN_HUMAN	29.08	3	3	7.09E+06	2	2	0
O15417|TNC18_HUMAN	29.07	1	1	1.33E+05	2	2	0
Q9BUJ2|HNRL1_HUMAN	128.16	12	12	4.78E+06	7	7	99.18
Q96JG9|ZN469_HUMAN	28.85	1	1	1.93E+06	2	2	0
P12111|CO6A3_HUMAN	28.72	1	1	3.04E+05	2	2	0
O14607|UTY_HUMAN	28.66	2	2	3.49E+05	2	1	0
Q10571|MN1_HUMAN	28.66	2	2	3.83E+07	2	2	0
P26368|U2AF2_HUMAN	95.12	12	12	2.90E+06	5	5	66.51
Q16629|SRSF7_HUMAN	63.53	13	13	3.24E+06	3	2	34.93
Q96RK0|CIC_HUMAN	28.59	3	3	1.36E+06	3	3	0
Q7Z5J4|RAI1_HUMAN	28.52	2	2	2.35E+06	2	2	0
P98088|MUC5A_HUMAN	28.44	1	1	4.76E+06	2	2	0
P22694|KAPCB_HUMAN	28.33	3	3	9.50E+04	1	1	0
O75128|COBL_HUMAN	28.18	1	1	1.53E+06	1	1	0
Q16531|DDB1_HUMAN	158.37	15	15	1.96E+07	14	14	130.24
Q6ZUT6|CO052_HUMAN	28.09	4	4	6.17E+06	2	2	0
Q9NTJ3|SMC4_HUMAN	28	1	1	3.04E+05	1	1	0

P35408|PE2R4_HUMAN	27.8	4	4	2.03E+05	1	1	0
Q8NFW1|COMA1_HUMAN	27.77	3	3	1.26E+06	2	2	0
P12270|TPR_HUMAN	27.72	1	1	3.45E+05	1	1	0
Q86V81|THOC4_HUMAN	82.04	11	11	2.38E+06	2	2	54.59
Q15147|PLCB4_HUMAN	27.42	2	2	1.55E+05	2	1	0
Q9NYQ7|CELR3_HUMAN	27.27	1	1	2.46E+05	2	2	0
Q96F45|ZN503_HUMAN	27.23	8	8	0.00E+00	2	2	0
Q5T953|IER5L_HUMAN	27.09	5	5	2.90E+05	1	1	0
Q5VZB9|DMRTA_HUMAN	26.9	4	4	0.00E+00	1	1	0
Q53GQ0|DHB12_HUMAN	26.86	5	5	2.92E+05	1	1	0
Q9NVI1|FANCI_HUMAN	26.79	0	0		1	0	0
A5YKK6|CNOT1_HUMAN	26.75	0	0	5.29E+04	1	1	0
Q9H223|EHD4_HUMAN	26.75	5	5	1.09E+05	1	1	0
Q03001|DYST_HUMAN	26.69	0	0	1.34E+06	2	2	0
Q9BVQ7|SPA5L_HUMAN	26.66	8	8	3.22E+05	2	2	0
Q00975|CAC1B_HUMAN	26.63	2	2	0.00E+00	2	2	0
O75376|NCOR1_HUMAN	26.53	1	1	0.00E+00	1	1	0
O60506|HNRPQ_HUMAN	160.04	19	19	5.23E+06	8	5	133.57
Q3T8J9|GON4L_HUMAN	26.35	2	2	6.20E+06	2	2	0
P08621|RU17_HUMAN	26.34	4	4	1.38E+06	2	2	0
Q8TB37|NUBPL_HUMAN	26.34	4	4	2.75E+06	1	1	0
Q9P2D8|UNC79_HUMAN	26.3	0	0	6.99E+05	1	1	0
Q7Z7G1|CLNK_HUMAN	26.11	2	2	5.70E+05	1	1	0
Q96D09|GASP2_HUMAN	26.08	2	2	8.12E+05	2	2	0
Q9H583|HEAT1_HUMAN	26.08	1	1	1.41E+05	2	2	0
Q9P2G4|MAP10_HUMAN	26	5	5	9.50E+06	2	2	0
P21817|RYR1_HUMAN	25.99	1	1	8.93E+05	2	2	0
Q8IW75|SPA12_HUMAN	25.99	2	2	1.00E+05	1	1	0
P12235|ADT1_HUMAN	103.41	25	25		5	0	77.5
Q96HR8|NAF1_HUMAN	25.84	3	3	1.04E+05	1	1	0
P51784|UBP11_HUMAN	25.75	1	1	1.16E+05	1	1	0
O14734|ACOT8_HUMAN	25.72	7	7	7.55E+05	1	1	0
Q9HCC9|LST2_HUMAN	25.71	1	1	2.09E+06	1	1	0
Q5T5C0|STXB5_HUMAN	25.7	1	1	1.50E+06	1	1	0
P22626|ROA2_HUMAN	157.61	30	30	1.68E+07	9	8	132.01
Q9Y2K5|R3HD2_HUMAN	25.59	1	1	3.12E+07	1	1	0
P46781|RS9_HUMAN	141.13	40	40	1.51E+07	11	11	115.55
P35568|IRS1_HUMAN	25.55	2	2	1.65E+05	1	1	0
Q8N1G0|ZN687_HUMAN	25.48	1	1	3.38E+05	1	1	0
Q9NZJ4|SACS_HUMAN	25.41	0	0	2.85E+05	1	1	0
Q9NYQ6|CELR1_HUMAN	25.38	1	1	2.98E+06	2	2	0
Q02388|CO7A1_HUMAN	49.2	4	4	4.57E+06	5	5	23.94
P34931|HS71L_HUMAN	216.9	40	40	7.01E+07	26	6	191.72
Q9NVN8|GNL3L_HUMAN	25.14	3	3	0.00E+00	1	1	0
A6NEL2|SWAHB_HUMAN	24.96	1	1	2.55E+05	1	1	0
P48426|PI42A_HUMAN	24.91	3	3	4.06E+05	1	1	0
Q14257|RCN2_HUMAN	195.8	28	28	3.40E+07	9	9	170.93
P82094|TMF1_HUMAN	24.85	1	1	5.87E+05	1	1	0
P35251|RFC1_HUMAN	24.79	1	1	8.02E+04	1	1	0
O15018|PDZD2_HUMAN	24.68	1	1	3.68E+06	2	2	0

Q9NQ11|AT132_HUMAN	24.61	2	2	1.24E+06	2	2	0
Q9Y6U3|ADSV_HUMAN	98.26	6	6	9.04E+05	3	3	73.79
P13942|COBA2_HUMAN	24.34	2	2	4.22E+05	2	2	0
Q9UNY4|TTF2_HUMAN	24.33	1	1	4.68E+06	1	1	0
Q9BRR8|GPTC1_HUMAN	24.3	1	1	1.70E+06	2	2	0
Q12830|BPTF_HUMAN	24.22	1	1	9.34E+05	2	2	0
Q9UJ99|CAD22_HUMAN	24.08	4	4	0.00E+00	2	2	0
P0CJ78|ZN865_HUMAN	24.04	1	1	6.90E+05	1	1	0
O94761|RECQ4_HUMAN	23.77	1	1	4.20E+06	1	1	0
Q96P44|COLA1_HUMAN	23.75	4	4	1.44E+06	2	2	0
Q70CQ2|UBP34_HUMAN	23.74	0	0	4.46E+05	1	1	0
Q96SB4|SRPK1_HUMAN	23.63	3	3	3.16E+05	1	1	0
Q9NRI5|DISC1_HUMAN	23.59	3	3	3.17E+06	2	2	0
H7BZ55|CRCC2_HUMAN	23.55	2	2	1.69E+06	2	2	0
Q13535|ATR_HUMAN	23.54	0	0	2.84E+05	1	1	0
C9JG80|NPIB4_HUMAN	23.46	1	1	2.25E+07	1	1	0
E5RHQ5|NPB11_HUMAN	23.46	1	1	2.25E+07	1	1	0
tr|K7EPK0|K7EPK0_HUMAN	23.42	2	2	8.58E+06	1	1	0
P98160|PGBM_HUMAN	23.2	0	0	0.00E+00	1	1	0
Q8NI27|THOC2_HUMAN	23.2	3	3	8.62E+05	2	2	0
P54259|ATN1_HUMAN	23.19	1	1	0.00E+00	1	1	0
P24043|LAMA2_HUMAN	23.13	0	0	1.54E+05	1	1	0
Q13202|DUS8_HUMAN	23.05	5	5	2.75E+05	2	2	0
P19622|HME2_HUMAN	23.03	8	8	0.00E+00	1	1	0
O14646|CHD1_HUMAN	23	1	1	6.10E+05	1	1	0
Q8N3C0|ASCC3_HUMAN	22.81	0	0	3.85E+05	1	1	0
Q99536|VAT1_HUMAN	22.81	4	4	1.41E+04	1	1	0
Q9P206|K1522_HUMAN	22.81	2	2	1.04E+05	1	1	0
P98179|RBM3_HUMAN	22.69	11	11	1.24E+06	1	1	0
Q9H568|ACTL8_HUMAN	22.67	2	2	4.30E+06	1	1	0
Q86YQ8|CPNE8_HUMAN	22.5	2	2		1	0	0
Q8IYJ1|CPNE9_HUMAN	22.5	2	2		1	0	0
Q96RG2|PASK_HUMAN	22.48	2	2	6.33E+05	1	1	0
A9QM74|IMA8_HUMAN	22.36	3	3	0.00E+00	1	1	0
P25440|BRD2_HUMAN	22.34	1	1	3.63E+05	1	1	0
P15502|ELN_HUMAN	47.19	20	20	3.46E+06	7	7	24.85
Q96HQ2|C2AIL_HUMAN	22.3	16	16	1.43E+06	1	1	0
Q96RY5|CRML_HUMAN	22.16	1	1	0.00E+00	1	1	0
Q9UQC2|GAB2_HUMAN	22.1	3	3	4.91E+05	1	1	0
Q4V328|GRAP1_HUMAN	22.07	2	2	1.67E+05	1	1	0
Q9ULZ9|MMP17_HUMAN	22	2	2	1.46E+06	1	1	0
P63173|RL38_HUMAN	78.81	34	34	4.26E+06	2	2	56.83
Q2M1Z3|RHG31_HUMAN	21.98	1	1	9.74E+04	1	1	0
P82279|CRUM1_HUMAN	21.89	1	1	5.09E+05	1	1	0
Q9UJK0|TSR3_HUMAN	21.87	11	11	2.03E+05	1	1	0
Q5TAP6|UT14C_HUMAN	21.8	2	2	9.82E+04	1	1	0
P20929|NEBU_HUMAN	313.76	20	20	2.86E+08	103	102	292.03
O95359|TACC2_HUMAN	21.7	1	1	1.75E+06	1	1	0
Q86V15|CASZ1_HUMAN	21.68	2	2	0.00E+00	1	1	0
Q9UPV7|PHF24_HUMAN	21.68	3	3	4.31E+05	1	1	0

O15054|KDM6B_HUMAN	21.6	1	1	1.83E+05	1	1	0
Q12906|ILF3_HUMAN	95.63	11	11	2.11E+06	5	5	74.08
P05141|ADT2_HUMAN	131.66	35	35	3.64E+06	8	2	110.16
O00763|ACACB_HUMAN	21.47	1	1	0.00E+00	1	1	0
P52790|HXK3_HUMAN	21.45	2	2	2.41E+06	1	1	0
Q9NRF2|SH2B1_HUMAN	21.37	3	3	3.31E+06	1	1	0
P21439|MDR3_HUMAN	21.29	1	1		1	0	0
Q2M3G0|ABCB5_HUMAN	21.29	1	1		1	0	0
Q8IXS0|F217A_HUMAN	21.26	2	2	5.76E+06	1	1	0
P60891|PRPS1_HUMAN	328.41	80	80	2.28E+08	58	13	307.19
Q9BXB1|LGR4_HUMAN	21.11	1	1	1.23E+05	1	1	0
Q9HBR0|S38AA_HUMAN	21.08	1	1	0.00E+00	1	1	0
A7E2Y1|MYH7B_HUMAN	20.99	1	1	4.30E+05	1	1	0
Q6ZUA9|MROH5_HUMAN	20.97	1	1	2.28E+05	1	1	0
Q8TC27|ADA32_HUMAN	20.93	1	1	7.01E+05	1	1	0
Q8IZC6|CORA1_HUMAN	20.92	1	1	3.65E+01	1	1	0
P42677|RS27_HUMAN	68.45	39	39	2.50E+06	3	3	47.63
Q9Y3L3|3BP1_HUMAN	20.8	3	3	1.52E+05	1	1	0
P25398|RS12_HUMAN	89.44	32	32	5.41E+06	6	6	68.65
P21108|PRPS3_HUMAN	288.98	44	44	3.28E+07	42	3	268.26
P40429|RL13A_HUMAN	125.95	35	35	3.11E+07	9	9	105.25
Q8ND56|LS14A_HUMAN	20.65	2	2	3.55E+05	1	1	0
A6NF01|P121B_HUMAN	20.59	3	3	0.00E+00	1	1	0
Q14997|PSME4_HUMAN	20.53	1	1	0.00E+00	1	1	0
O95793|STAU1_HUMAN	44.14	2	2	2.37E+05	1	1	23.65
O14777|NDC80_HUMAN	20.46	2	2	4.48E+05	1	1	0
P17600|SYN1_HUMAN	20.44	3	3	0.00E+00	1	1	0
P25311|ZA2G_HUMAN	79.94	9	9	3.39E+06	2	2	59.53
Q8IYL2|TRM44_HUMAN	20.4	4	4	2.27E+05	1	1	0
O76031|CLPX_HUMAN	20.38	2	2	3.10E+05	1	1	0
P50607|TUB_HUMAN	20.34	3	3	7.09E+06	1	1	0
Q6P1M3|L2GL2_HUMAN	20.34	1	1	3.23E+05	1	1	0
Q9UJ37|SIA7B_HUMAN	20.33	4	4	5.04E+05	1	1	0
Q6PID8|KLD10_HUMAN	20.25	5	5	4.96E+06	1	1	0
Q8N9T8|KRI1_HUMAN	20.21	2	2	0.00E+00	1	1	0
P62906|RL10A_HUMAN	143.34	33	33	1.21E+07	8	8	123.14
Q8N9H9|CA127_HUMAN	20.2	4	4	0.00E+00	1	1	0
Q9Y261|FOXA2_HUMAN	20.17	2	2	1.97E+06	1	1	0
Q86UU1|PHLB1_HUMAN	20.05	1	1	9.86E+04	1	1	0
Q8IVT2|MISP_HUMAN	20.04	2	2	0.00E+00	1	1	0
P35268|RL22_HUMAN	125.72	52	52	1.00E+07	5	5	105.92
P11142|HSP7C_HUMAN	331.13	66	66	6.51E+08	55	39	311.68
P12236|ADT3_HUMAN	130.56	42	42	3.34E+06	9	2	111.12
P17066|HSP76_HUMAN	205.54	35	35	2.34E+07	21	7	186.31
P11940|PABP1_HUMAN	154.42	23	23	3.65E+06	11	3	135.76
Q08945|SSRP1_HUMAN	74.89	8	8	1.03E+06	4	4	56.72
P05388|RLA0_HUMAN	244.99	54	54	4.44E+07	20	13	226.89
P12273|PIP_HUMAN	124.74	36	36	1.28E+07	5	5	107.12
P62424|RL7A_HUMAN	214.77	39	39	6.92E+07	15	15	197.55
Q14103|HNRPD_HUMAN	86.69	9	9	7.34E+06	3	2	69.72

Q14152|EIF3A_HUMAN	54.53	2	2	3.04E+06	2	2	38.21
Q02878|RL6_HUMAN	184.2	44	44	4.91E+07	13	13	168.07
P46782|RS5_HUMAN	108.48	23	23	6.46E+06	4	4	92.81
Q2VWA4|SKOR2_HUMAN	39.44	8	8	1.73E+06	4	4	23.97
P62316|SMD2_HUMAN	51.43	24	24	2.10E+06	2	2	36.14
Q9H361|PABP3_HUMAN	107.7	9	9		5	0	92.46
Q9P281|BAHC1_HUMAN	35.92	1	1	2.50E+06	2	2	20.81
Q6P2Q9|PRP8_HUMAN	37.54	1	1	4.04E+05	3	2	23.23
P11908|PRPS2_HUMAN	317.8	74	74	5.33E+08	56	18	303.8
Q9Y3U8|RL36_HUMAN	85.1	34	34	7.64E+06	5	5	71.16
O00425|IF2B3_HUMAN	81.31	9	9	1.26E+06	3	3	67.82
O95218|ZRAB2_HUMAN	38.74	6	6	8.22E+05	2	2	25.61
P46821|MAP1B_HUMAN	41.11	1	1	0.00E+00	2	1	28.18
Q96AG4|LRC59_HUMAN	59.31	8	8	1.07E+06	1	1	46.42
P62847|RS24_HUMAN	76.66	29	29	9.01E+06	3	3	64.03
O75643|U520_HUMAN	87.9	3	3	1.12E+06	5	5	75.55
Q13310|PABP4_HUMAN	112.11	8	8	3.44E+05	4	1	99.84
Q08211|DHX9_HUMAN	206.14	27	27	7.42E+07	25	25	193.89
P61978|HNRPK_HUMAN	149.74	21	21	6.42E+06	6	6	137.6
Q07020|RL18_HUMAN	161.1	35	35	5.16E+07	8	8	149.11
P15924|DESP_HUMAN	146.14	7	7	1.15E+07	16	16	134.32
P17707|DCAM_HUMAN	36.57	7	7	1.62E+06	2	2	24.84
O96019|ACL6A_HUMAN	45.59	10	10	1.25E+06	2	2	33.9
P18124|RL7_HUMAN	166.47	45	45	4.44E+07	12	12	154.94
O60832|DKC1_HUMAN	48.65	4	4	1.29E+06	1	1	37.56
P54652|HSP72_HUMAN	238.16	35	35	4.00E+07	28	12	227.09
P62241|RS8_HUMAN	176.33	60	60	4.48E+07	11	11	165.48
P27635|RL10_HUMAN	87.42	21	21	4.79E+06	3	3	76.68
Q14558|KPRA_HUMAN	322.11	85	85	1.62E+09	51	41	311.51
P61313|RL15_HUMAN	150.07	42	42	3.37E+07	9	9	139.47
tr|A0A0A6YYG9|A0A0A6YY(	129.73	8	8		4	0	119.19
Q6ZVL6|K154L_HUMAN	39.41	3	3	2.43E+06	3	2	29.05
Q01813|PFKAP_HUMAN	154.47	15	15	2.92E+06	10	7	144.53
Q8IYB3|SRRM1_HUMAN	103.19	8	8	3.60E+06	3	3	93.34
Q5T749|KPRP_HUMAN	69.72	9	9	2.37E+06	4	4	59.99
P0DMV8|HS71A_HUMAN	259.52	67	67	6.43E+07	41	18	249.8
P0DMV9|HS71B_HUMAN	259.52	67	67	6.43E+07	41	18	249.8
Q92841|DDX17_HUMAN	55.18	2	2		1	0	45.96
P19338|NUCL_HUMAN	286.79	52	52	2.42E+08	47	46	277.99
O60902|SHOX2_HUMAN	35.03	12	12	8.39E+04	3	3	26.28
P08237|PFKAM_HUMAN	46.7	4	4	1.82E+05	3	2	38.04
P22492|H1T_HUMAN	47.37	5	5		1	0	38.82
P02768|ALBU_HUMAN	130.51	8	8	3.60E+06	6	6	122.1
P49721|PSB2_HUMAN	43.35	5	5	5.97E+05	1	1	35.01
P61353|RL27_HUMAN	94.28	49	49	2.77E+07	8	8	86.09
A7E2V4|ZSWM8_HUMAN	38.7	6	6	9.72E+05	5	5	30.75
P36578|RL4_HUMAN	207.22	44	44	8.56E+07	20	20	199.48
P31153|METK2_HUMAN	71.38	6	6	8.23E+05	2	1	63.78
P49207|RL34_HUMAN	42.67	13	13	9.32E+06	2	2	35.44
O43143|DHX15_HUMAN	73.33	5	5	8.32E+05	3	3	66.75

Q99729|ROAA_HUMAN	60.13	5	5	2.21E+06	2	1	53.86
Q15397|PUM3_HUMAN	40.73	2	2	5.09E+05	1	1	34.64
Q9Y2T7|YBOX2_HUMAN	136.6	24	24	9.67E+05	8	4	130.56
P46778|RL21_HUMAN	97.83	15	15	5.59E+06	3	3	92.08
P06702|S10A9_HUMAN	69.57	31	31	2.46E+06	3	3	63.84
P62805|H4_HUMAN	71.52	29	29	4.45E+06	3	3	65.91
A6NC98|CC88B_HUMAN	28.99	1	1	7.05E+05	1	1	23.59
P13645|K1C10_HUMAN	331.64	68	68	6.06E+08	55	47	326.39
Q9H4A3|WNK1_HUMAN	26.63	1	1	3.34E+06	1	1	21.48
Q9BZL6|KPCD2_HUMAN	27.11	2	2	2.12E+06	1	1	22.02
P84098|RL19_HUMAN	101.1	14	14	5.70E+06	3	3	96.24
P62266|RS23_HUMAN	111.33	50	50	5.38E+06	4	4	106.56
Q9UQ35|SRRM2_HUMAN	35.09	1	1	8.17E+05	1	1	30.37
P81605|DCD_HUMAN	184.99	75	75	2.06E+08	16	16	180.34
P62273|RS29_HUMAN	44.45	34	34	1.71E+06	2	2	40.37
O95831|AIFM1_HUMAN	107.46	14	14	3.38E+06	5	5	103.6
Q15811|ITSN1_HUMAN	28.71	1	1	2.61E+06	1	1	24.99
tr|J3KR12|J3KR12_HUMAN	29.28	7	7	2.33E+06	2	2	25.57
A3KMH1|VWA8_HUMAN	30.54	1	1	2.31E+05	1	1	26.9
Q14315|FLNC_HUMAN	55.69	1	1		2	0	52.41
P47914|RL29_HUMAN	84.7	14	14	1.06E+07	2	2	81.53
Q08188|TGM3_HUMAN	35.69	4	4	7.31E+05	2	2	32.67
P35249|RFC4_HUMAN	28.9	6	6	1.73E+05	1	1	26.03
P62899|RL31_HUMAN	114.91	35	35	1.28E+07	4	4	112.37
P67809|YBOX1_HUMAN	171.81	34	34	1.31E+07	10	5	169.45
P04264|K2C1_HUMAN	349.53	77	77	1.30E+09	94	81	347.3
Q2M2I5|K1C24_HUMAN	84.87	5	5	1.09E+06	3	1	82.82
O14880|MGST3_HUMAN	51.56	9	9	1.67E+05	1	1	49.69
Q15046|SYK_HUMAN	33.94	4	4	3.54E+05	2	2	32.19
Q01469|FABP5_HUMAN	34.11	7	7	1.55E+05	1	1	32.57
Q9NUL3|STAU2_HUMAN	57.77	2	2	4.06E+05	1	1	56.48
O76021|RL1D1_HUMAN	56.24	7	7	2.44E+06	2	2	54.95
P09429|HMGB1_HUMAN	48.1	7	7	4.48E+05	1	1	46.93
P31151|S10A7_HUMAN	70.13	12	12	1.65E+06	2	2	69.11
Q6ZRS2|SRCAP_HUMAN	32.91	0	0	3.09E+06	1	1	31.9
P00403|COX2_HUMAN	32.37	4	4	5.12E+05	1	1	31.46
Q99848|EBP2_HUMAN	115.78	19	19	1.40E+06	5	5	114.9
P62753|RS6_HUMAN	138.11	25	25	7.91E+06	8	8	137.24
P30050|RL12_HUMAN	160.3	43	43	2.57E+07	5	5	159.61
Q96M86|DNHD1_HUMAN	22.02	0	0	1.63E+06	1	1	21.6
P09661|RU2A_HUMAN	34.74	5	5	4.11E+05	1	1	34.49
P17655|CAN2_HUMAN	20.38	2	2	1.63E+06	1	1	20.13
O60256|KPRB_HUMAN	325.26	81	81	3.64E+09	67	57	325.06
Q9H4B7|TBB1_HUMAN	137.18	14	14	0.00E+00	8	1	137.12
P13646|K1C13_HUMAN	153.69	20	20	5.02E+06	12	3	153.82
Q7Z3Y8|K1C27_HUMAN	107.11	9	9	1.61E+06	5	1	107.24
Q9ULU8|CAPS1_HUMAN	28.56	3	3	0.00E+00	3	3	28.91
Q5JQF8|PAP1M_HUMAN	46.2	6	6		1	0	46.61
P23458|JAK1_HUMAN	94.08	7	7	2.88E+06	6	6	94.52
P62314|SMD1_HUMAN	49.67	11	11	1.66E+06	1	1	50.25

Q15366|PCBP2_HUMAN	37.22	4	4	2.85E+05	1	1	37.88
Q5T750|XP32_HUMAN	30.08	3	3	4.35E+05	1	1	30.81
P62910|RL32_HUMAN	43.65	10	10	2.15E+05	1	1	44.47
Q99460|PSMD1_HUMAN	61.51	3	3	4.03E+05	2	2	62.41
Q04695|K1C17_HUMAN	162.75	31	31	3.18E+06	15	6	163.71
O00303|EIF3F_HUMAN	73.01	5	5	3.46E+05	1	1	73.98
Q01130|SRSF2_HUMAN	26.64	4	4	1.40E+06	1	1	27.67
Q9BRL6|SRSF8_HUMAN	26.64	3	3	1.40E+06	1	1	27.67
P20908|CO5A1_HUMAN	26.31	2	2	1.52E+06	2	2	27.38
P08123|CO1A2_HUMAN	23.52	2	2	4.44E+05	2	2	24.69
P11388|TOP2A_HUMAN	78.19	3	3	3.63E+05	4	2	79.44
P52597|HNRPF_HUMAN	123.49	14	14	1.80E+06	4	2	124.79
O15084|ANR28_HUMAN	49.02	3	3	5.71E+05	2	2	50.44
Q02880|TOP2B_HUMAN	60.5	3	3	4.44E+05	4	2	62.18
P78362|SRPK2_HUMAN	68.89	4	4	1.22E+06	2	2	70.82
Q3V6T2|GRDN_HUMAN	22.99	1	1	1.87E+06	1	1	24.94
Q9UPA5|BSN_HUMAN	32.47	1	1	5.26E+06	3	3	34.56
Q92522|H1X_HUMAN	93.37	10	10	6.10E+06	2	2	95.81
P19012|K1C15_HUMAN	149.95	22	22	1.72E+05	10	2	152.71
P48741|HSP77_HUMAN	134.34	14	14		6	0	137.24
P42858|HD_HUMAN	27.48	0	0		1	0	30.48
Q9BQ39|DDX50_HUMAN	60.04	2	2		1	0	63.07
P58107|EPIPL_HUMAN	35.12	1	1	2.17E+05	3	3	38.2
Q13439|GOGA4_HUMAN	30.6	1	1	4.27E+05	2	1	33.72
P83731|RL24_HUMAN	100.17	19	19	1.56E+07	3	3	103.4
P02461|CO3A1_HUMAN	56.8	15	15	4.20E+06	10	10	60.05
O75369|FLNB_HUMAN	62.27	2	2	8.32E+06	3	2	65.54
P59998|ARPC4_HUMAN	138.82	34	34	9.35E+06	5	1	142.55
O76013|KRT36_HUMAN	42.46	1	1		1	0	46.3
P02533|K1C14_HUMAN	231.78	67	67	5.14E+06	33	2	235.67
P35527|K1C9_HUMAN	339.31	79	79	9.78E+08	57	56	343.3
P42766|RL35_HUMAN	73.41	22	22	8.62E+06	3	3	77.7
Q9P2P6|STAR9_HUMAN	34.95	1	1	2.40E+06	3	3	39.48
P62333|PRS10_HUMAN	37.99	4	4	2.12E+05	1	1	43.07
Q5XKE5|K2C79_HUMAN	131.83	17	17	4.20E+05	10	4	136.92
P19013|K2C4_HUMAN	99.94	12	12	2.86E+05	4	1	105.08
P62269|RS18_HUMAN	98.53	32	32	7.66E+06	6	6	104.32
Q9Y6Y0|NS1BP_HUMAN	303.01	73	73	7.88E+08	45	45	309.15
P62277|RS13_HUMAN	122.94	44	44	1.03E+07	8	7	129.3
P14136|GFAP_HUMAN	61.85	6	6	7.33E+05	2	1	68.23
Q9NWQ4|GPT2L_HUMAN	20.04	4	4	3.70E+05	1	1	26.51
Q3ZCM7|TBB8_HUMAN	193.5	42	42	2.32E+06	20	3	200.04
Q6KB66|K2C80_HUMAN	61.62	5	5	8.26E+05	2	1	68.23
Q9Y2W1|TR150_HUMAN	24.51	1	1	2.04E+05	1	1	31.17
B0I1T2|MYO1G_HUMAN	39.95	1	1		1	0	46.72
A6NNZ2|TBB8L_HUMAN	191.28	35	35	1.53E+05	19	1	198.08
P23396|RS3_HUMAN	121.85	34	34	1.28E+07	8	8	128.65
Q9UI10|EI2BD_HUMAN	41.54	6	6	2.11E+05	2	2	48.67
Q08554|DSC1_HUMAN	43.69	2	2	3.54E+05	1	1	50.99
tr|Q5TEC6|Q5TEC6_HUMAN	22.41	7	7	4.76E+05	1	1	29.72

tr|A0A0B4J269|A0A0B4J269	266.01	38	38	3.30E+05	36	2	273.56
Q96HS1|PGAM5_HUMAN	46.81	10	10	1.13E+06	3	3	54.54
Q9Y230|RUVB2_HUMAN	199.12	41	41	2.18E+07	20	20	206.95
P08727|K1C19_HUMAN	109.56	8	8		5	0	117.46
A6NHL2|TBAL3_HUMAN	70.01	8	8	4.21E+04	3	1	78.01
Q00325|MPCP_HUMAN	29.08	3	3	9.98E+05	1	1	37.09
Q8WXR4|MYO3B_HUMAN	36.73	1	1		1	0	45
P61513|RL37A_HUMAN	68.62	20	20	2.72E+06	1	1	76.94
Q9H7D7|WDR26_HUMAN	40.1	3	3	3.96E+05	1	1	48.42
P49411|EFTU_HUMAN	163.06	23	23	2.51E+07	9	9	171.41
P62249|RS16_HUMAN	115.47	37	37	1.23E+07	5	5	123.95
P35908|K22E_HUMAN	312.19	85	85	3.15E+08	79	68	320.74
Q9Y5B9|SP16H_HUMAN	84.01	6	6	4.64E+06	5	5	92.69
P50914|RL14_HUMAN	112.14	28	28	3.66E+07	6	6	121.22
P46776|RL27A_HUMAN	90.37	16	16	7.43E+06	3	3	99.51
Q13595|TRA2A_HUMAN	35.08	9	9	3.86E+05	2	2	44.22
Q13509|TBB3_HUMAN	269.96	66	66		36	0	279.19
O60814|H2B1K_HUMAN	64.52	26	26		3	0	74.12
P58876|H2B1D_HUMAN	64.52	26	26		3	0	74.12
P62807|H2B1C_HUMAN	64.52	26	26		3	0	74.12
Q5QNW6|H2B2F_HUMAN	64.52	26	26		3	0	74.12
Q93079|H2B1H_HUMAN	64.52	26	26		3	0	74.12
Q99877|H2B1N_HUMAN	64.52	26	26		3	0	74.12
Q99879|H2B1M_HUMAN	64.52	26	26		3	0	74.12
Q99880|H2B1L_HUMAN	64.52	26	26		3	0	74.12
P13647|K2C5_HUMAN	213.01	56	56	7.59E+07	42	31	222.61
P07437|TBB5_HUMAN	300.16	75	75	2.79E+08	45	8	309.84
P08779|K1C16_HUMAN	235.44	63	63	7.39E+07	33	17	245.25
P25940|CO5A3_HUMAN	23.43	3	3	8.45E+05	2	2	33.3
P62701|RS4X_HUMAN	97.6	20	20	9.36E+06	5	5	107.6
Q7Z794|K2C1B_HUMAN	144.87	14	14	5.94E+07	9	6	155
P0CG38|POTEI_HUMAN	199.9	23	23	3.68E+07	29	5	210.07
Q5T011|SZT2_HUMAN	25.6	1	1	3.77E+06	2	2	36.04
Q01546|K22O_HUMAN	113.93	9	9	2.36E+05	6	1	124.59
P26373|RL13_HUMAN	88.05	24	24	2.19E+07	6	6	98.72
Q6UB35|C1TM_HUMAN	26.9	3	3	8.74E+05	2	2	37.72
P0CG39|POTEJ_HUMAN	161.25	9	9		11	0	172.13
Q562R1|ACTBL_HUMAN	180.87	41	41	3.19E+08	18	10	192.15
Q8NHW5|RLA0L_HUMAN	162.55	33	33	9.08E+05	8	1	173.87
P62917|RL8_HUMAN	127.53	37	37	1.96E+07	7	7	139.01
P60842|IF4A1_HUMAN	42.44	6	6		2	0	53.99
Q14240|IF4A2_HUMAN	42.44	6	6		2	0	53.99
Q8N1N4|K2C78_HUMAN	117.61	7	7	2.26E+07	6	4	129.28
Q09028|RBBP4_HUMAN	38.12	4	4	2.13E+06	2	2	49.94
P12004|PCNA_HUMAN	37.96	7	7	2.19E+05	1	1	49.87
P16989|YBOX3_HUMAN	135.46	21	21	5.98E+04	7	2	147.43
P35900|K1C20_HUMAN	79.38	4	4		4	0	91.62
Q9BQA1|MEP50_HUMAN	288.92	88	88	8.14E+08	28	28	301.17
Q7Z5P9|MUC19_HUMAN	50.89	1	1	5.75E+06	6	6	63.14
P62995|TRA2B_HUMAN	46.81	6	6	8.97E+05	1	1	59.14

P02462|CO4A1_HUMAN	20.88	2	2	3.13E+05	1	1	33.33
Q02543|RL18A_HUMAN	125.95	33	33	1.61E+07	8	8	138.41
Q86U42|PABP2_HUMAN	39.46	15	15	8.95E+04	4	4	52.16
O95757|HS74L_HUMAN	111.79	11	11	2.50E+06	6	4	124.57
P62750|RL23A_HUMAN	106.35	21	21	6.96E+06	4	4	119.41
P62937|PPIA_HUMAN	80.8	18	18	2.21E+06	3	3	93.86
P14678|RSMB_HUMAN	42.27	7	7	7.98E+06	3	3	55.36
O60882|MMP20_HUMAN	23.69	2	2	2.34E+06	1	1	36.81
Q13748|TBA3C_HUMAN	272.03	56	56		27	0	285.24
P22087|FBRL_HUMAN	27.21	3	3		1	0	40.45
Q9H3K6|BOLA2_HUMAN	51.39	19	19	2.96E+05	1	1	64.77
tr|H3BVE0|H3BVE0_HUMAN	51.39	6	6	2.96E+05	1	1	64.77
P68363|TBA1B_HUMAN	284.48	64	64		32	0	298.01
Q9BYX7|ACTBM_HUMAN	204.09	34	34	1.02E+07	19	2	218.1
Q9Y265|RUVB1_HUMAN	204.37	43	43	5.77E+07	17	17	218.46
O95678|K2C75_HUMAN	130.99	7	7		6	0	145.18
Q6PEY2|TBA3E_HUMAN	262.55	51	51		23	0	276.89
Q9BQE3|TBA1C_HUMAN	276.77	64	64	1.94E+06	29	2	291.19
Q13162|PRDX4_HUMAN	63.23	12	12	6.11E+05	2	1	77.76
P01036|CYTS_HUMAN	37.14	13	13	6.32E+05	2	2	51.69
P29508|SPB3_HUMAN	30.96	3	3	2.02E+05	1	1	45.62
Q7Z3Y7|K1C28_HUMAN	80.17	5	5		3	0	94.87
P09104|ENOG_HUMAN	62.31	4	4		1	0	77.04
P13929|ENOB_HUMAN	62.31	4	4		1	0	77.04
O60841|IF2P_HUMAN	23.95	2	2	2.89E+05	2	2	38.69
P38606|VATA_HUMAN	38.64	2	2	5.32E+04	1	1	53.38
P68371|TBB4B_HUMAN	288.23	74	74	9.86E+06	42	1	303.01
Q02413|DSG1_HUMAN	44.07	2	2	1.46E+06	1	1	59.04
P39023|RL3_HUMAN	157.56	26	26	2.38E+07	10	10	172.66
Q1KMD3|HNRL2_HUMAN	22.89	1	1	2.47E+05	1	1	37.99
A5A3E0|POTEF_HUMAN	217.68	22	22	2.37E+08	29	5	232.87
Q99456|K1C12_HUMAN	79.14	4	4		3	0	94.34
P05783|K1C18_HUMAN	42.46	2	2		1	0	57.73
Q71U36|TBA1A_HUMAN	283.24	64	64		31	0	298.68
O95864|FADS2_HUMAN	68.22	6	6	3.63E+05	2	2	84.3
P35232|PHB_HUMAN	107.06	21	21	1.39E+06	4	4	123.33
P68366|TBA4A_HUMAN	264.33	56	56	4.51E+06	28	3	280.96
P38405|GNAL_HUMAN	34.4	3	3		1	0	51.16
P62913|RL11_HUMAN	64.86	8	8	6.42E+06	1	1	81.86
O15371|EIF3D_HUMAN	49.69	5	5	7.02E+05	2	2	66.81
Q9BRJ6|CG050_HUMAN	82.27	22	22	1.88E+06	2	2	99.45
O15511|ARPC5_HUMAN	105.29	34	34	5.19E+06	4	3	122.65
Q9NY65|TBA8_HUMAN	235.34	38	38	9.39E+05	17	1	252.85
P27708|PYR1_HUMAN	158.21	8	8	9.80E+06	14	14	175.81
P12035|K2C3_HUMAN	126.72	16	16	2.35E+06	9	2	144.4
Q13885|TBB2A_HUMAN	281.72	73	73	4.88E+05	40	1	299.52
Q9BUF5|TBB6_HUMAN	209.16	36	36		21	0	227.12
Q7RTS7|K2C74_HUMAN	78.84	16	16	3.32E+06	5	3	96.99
Q9BVA1|TBB2B_HUMAN	283.86	74	74	4.15E+06	41	2	302.24
P32119|PRDX2_HUMAN	85.58	20	20	9.24E+05	3	2	104.02

O15145|ARPC3_HUMAN	121.2	25	25	2.25E+07	5	5	139.68
P49902|5NTC_HUMAN	59.35	5	5	2.89E+06	2	2	78.03
O75629|CREG1_HUMAN	31.25	10	10	4.49E+05	1	1	50.07
P48668|K2C6C_HUMAN	236.04	54	54	2.81E+06	48	5	255
P68133|ACTS_HUMAN	225.19	59	59	2.04E+03	29	1	244.26
Q9UHB6|LIMA1_HUMAN	138.95	14	14	6.84E+06	8	8	158.02
P04350|TBB4A_HUMAN	266.4	66	66		33	0	285.64
Q99714|HCD2_HUMAN	96.79	22	22	1.82E+06	3	3	116.21
Q15393|SF3B3_HUMAN	51.16	2	2	1.75E+06	2	2	70.81
Q06830|PRDX1_HUMAN	77.04	19	19	1.03E+06	4	2	97.21
P05787|K2C8_HUMAN	105.16	11	11	4.46E+05	6	2	125.33
O43933|PEX1_HUMAN	25.73	1	1	2.95E+05	1	1	46.06
P05997|CO5A2_HUMAN	24.32	5	5	8.27E+04	2	2	44.83
O00743|PPP6_HUMAN	46.99	4	4	7.41E+05	1	1	67.72
Q5VTE0|EF1A3_HUMAN	186.28	30	30	1.99E+07	14	8	207.08
P68032|ACTC_HUMAN	225.25	59	59		29	0	246.06
Q9BTM1|H2AJ_HUMAN	122.25	36	36	2.20E+05	4	1	143.21
P40227|TCPZ_HUMAN	128.15	14	14	3.61E+06	5	5	149.61
Q9ULV0|MYO5B_HUMAN	127.68	9	9	5.40E+06	11	10	149.24
Q9Y657|SPIN1_HUMAN	115.59	33	33	4.27E+06	6	4	137.52
P02458|CO2A1_HUMAN	29.31	5	5	5.22E+07	3	3	51.57
Q8IZ41|RASEF_HUMAN	29.97	5	5	9.60E+04	2	2	52.31
Q92499|DDX1_HUMAN	31.28	1	1	1.63E+05	1	1	54.38
Q07065|CKAP4_HUMAN	86.11	12	12	1.20E+06	5	5	109.54
Q99873|ANM1_HUMAN	54.71	3	3	8.99E+05	1	1	78.15
P18621|RL17_HUMAN	106	22	22	4.16E+06	3	3	129.5
tr|A0A0A6YYL6|A0A0A6YYL	106	18	18	4.16E+06	3	3	129.5
Q8WZ42|TITIN_HUMAN	24.33	0	0	6.24E+05	2	2	48
P62736|ACTA_HUMAN	215.43	59	59	5.10E+06	28	1	239.16
P60174|TPIS_HUMAN	26.83	10	10	3.31E+05	1	1	50.79
P62888|RL30_HUMAN	101.09	41	41	2.67E+07	3	3	125.14
P62854|RS26_HUMAN	105.77	31	31	1.30E+07	3	3	129.97
Q86Y46|K2C73_HUMAN	74.31	10	10	1.69E+06	4	1	98.68
Q06265|EXOS9_HUMAN	26.26	2	2	2.30E+05	1	1	50.89
O14744|ANM5_HUMAN	290	64	64	1.25E+09	47	46	314.64
P08729|K2C7_HUMAN	85.24	14	14		5	0	109.89
P05386|RLA1_HUMAN	82.69	34	34	2.18E+07	2	2	107.37
Q13428|TCOF_HUMAN	20.35	1	1	2.52E+05	1	1	45.19
P52701|MSH6_HUMAN	57.62	3	3	8.10E+05	3	3	82.63
P33993|MCM7_HUMAN	148.3	14	14	4.70E+06	7	7	173.7
tr|S4R435|S4R435_HUMAN	33.75	3	3	1.39E+06	1	1	59.27
Q14194|DPYL1_HUMAN	33.02	4	4	2.14E+05	2	1	58.59
Q9NQX4|MYO5C_HUMAN	96.17	3	3	2.00E+05	4	1	122.02
Q9H857|NT5D2_HUMAN	36.75	4	4	9.46E+05	2	2	62.7
P49368|TCPG_HUMAN	99.97	9	9	4.73E+06	4	4	126.06
P37108|SRP14_HUMAN	54.27	32	32	4.00E+06	2	2	80.96
P02538|K2C6A_HUMAN	231.51	45	45	2.24E+06	42	1	258.53
Q13868|EXOS2_HUMAN	30.01	4	4	3.44E+05	1	1	57.19
P23588|IF4B_HUMAN	87.83	8	8	5.59E+06	4	4	115.22
P23528|COF1_HUMAN	132.06	43	43	7.68E+06	5	5	159.5

Q8TDN6|BRX1_HUMAN	101.1	25	25	3.97E+06	7	7	128.61
P98175|RBM10_HUMAN	110.27	14	14	1.11E+07	7	7	137.85
Q9BPX5|ARP5L_HUMAN	120.57	46	46	1.26E+07	5	4	148.21
O60884|DNJA2_HUMAN	48.41	6	6	7.69E+05	1	1	76.42
P04259|K2C6B_HUMAN	225.58	45	45	3.19E+05	37	1	253.71
P50990|TCPQ_HUMAN	98.8	11	11	2.94E+06	5	5	126.94
P01893|HLAH_HUMAN	40.61	10	10	1.71E+06	2	1	68.75
P16104|H2AX_HUMAN	131.38	36	36	2.42E+06	5	2	159.53
P15880|RS2_HUMAN	107.52	17	17	6.60E+06	6	6	135.81
P61247|RS3A_HUMAN	95.25	28	28	9.61E+06	7	7	123.68
P26599|PTBP1_HUMAN	78.79	8	8	4.04E+05	2	2	107.36
Q3SY84|K2C71_HUMAN	68.54	8	8		3	0	97.36
Q9Y2H1|ST38L_HUMAN	89.03	13	13	4.38E+05	6	2	117.94
P62851|RS25_HUMAN	65.86	15	15	2.54E+06	2	2	94.93
P62081|RS7_HUMAN	102.81	19	19	2.95E+06	4	4	132.4
Q01082|SPTB2_HUMAN	289.62	40	40	1.27E+08	73	67	319.3
O14908|GIPC1_HUMAN	25.43	5	5	5.11E+04	1	1	56.49
P05109|S10A8_HUMAN	39.61	24	24	1.68E+06	2	2	71.14
Q08043|ACTN3_HUMAN	50.79	3	3	9.05E+05	2	1	83.4
P31943|HNRH1_HUMAN	120	11	11		4	0	152.84
P29692|EF1D_HUMAN	53.47	9	9	5.32E+05	2	2	86.31
Q13813|SPTN1_HUMAN	326.33	42	42	1.79E+08	86	85	359.25
P62263|RS14_HUMAN	48.87	7	7	8.91E+05	1	1	82.11
P62280|RS11_HUMAN	69.72	23	23	2.97E+06	3	3	103.14
Q5JUX0|SPIN3_HUMAN	102.15	16	16	5.47E+05	3	1	135.87
B5ME19|EIFCL_HUMAN	60.29	3	3	6.10E+05	2	2	94.13
Q99613|EIF3C_HUMAN	60.29	3	3	6.10E+05	2	2	94.13
Q15208|STK38_HUMAN	225.13	53	53	2.03E+08	23	19	259.07
P01615|KV202_HUMAN	35.54	12	12	1.01E+06	1	1	70.58
P30154|2AAB_HUMAN	29.11	2	2	9.71E+04	1	1	64.49
P60866|RS20_HUMAN	42.33	10	10	5.04E+05	1	1	78.13
Q31612|1B73_HUMAN	26.67	10	10	1.46E+06	2	1	63.35
O00159|MYO1C_HUMAN	227.55	36	36	4.23E+07	32	31	264.31
P43490|NAMPT_HUMAN	37.61	2	2	1.47E+05	1	1	74.68
Q6S8J3|POTEE_HUMAN	233.62	23	23		30	0	270.75
Q15057|ACAP2_HUMAN	112.87	9	9	3.01E+06	5	5	150.13
P12956|XRCC6_HUMAN	135.81	17	17	5.82E+06	8	8	173.89
P63261|ACTG_HUMAN	323.15	81	81	8.05E+07	55	1	361.68
P50991|TCPD_HUMAN	75.49	5	5	2.39E+06	2	2	114.78
P60709|ACTB_HUMAN	322.67	81	81	2.36E+07	55	1	362.05
P26641|EF1G_HUMAN	59.02	5	5	1.10E+06	2	2	98.55
Q9UBC5|MYO1A_HUMAN	91.42	2	2		3	0	131.22
P29144|TPP2_HUMAN	107.42	5	5	1.49E+06	4	4	147.94
P55209|NP1L1_HUMAN	101.37	12	12	7.49E+06	3	3	142.17
P07195|LDHB_HUMAN	28.01	2	2	1.03E+05	1	1	68.88
Q92900|RENT1_HUMAN	29.37	1	1	1.61E+05	1	1	70.41
Q9BVP2|GNL3_HUMAN	32.72	3	3	7.47E+04	1	1	73.86
P00338|LDHA_HUMAN	34.81	5	5	3.84E+05	2	2	76.16
Q9BUA3|CK084_HUMAN	120.56	25	25	7.62E+06	6	6	161.92
P62318|SMD3_HUMAN	65.34	15	15	1.59E+07	2	2	106.77

Q9NSB2|KRT84_HUMAN	53.79	7	7	1.58E+06	3	2	95.35
P62158|CALM_HUMAN	116.86	31	31	3.93E+07	3	3	158.61
O00571|DDX3X_HUMAN	57.73	3	3	4.09E+05	2	2	99.68
P41091|IF2G_HUMAN	101.97	7	7	2.25E+06	3	3	144.02
Q05639|EF1A2_HUMAN	153.49	20	20	6.04E+05	8	2	195.66
Q9Y383|LC7L2_HUMAN	72.99	4	4	2.15E+06	1	1	115.72
Q5SWX8|ODR4_HUMAN	46.6	7	7	1.02E+06	2	2	89.63
P60660|MYL6_HUMAN	66.65	20	20	1.93E+06	3	3	110.1
P13010|XRCC5_HUMAN	73.9	13	13	2.54E+06	5	5	118.13
Q14CN4|K2C72_HUMAN	35.11	1	1		1	0	79.54
O75366|AVIL_HUMAN	301.61	59	59	3.41E+08	48	46	347.37
P30041|PRDX6_HUMAN	110.03	20	20	2.99E+06	4	4	158.22
O94832|MYO1D_HUMAN	111.92	6	6	1.71E+06	6	5	160.46
P01614|KV201_HUMAN	112.83	14	14		3	0	162.31
P06310|KV206_HUMAN	112.83	12	12		3	0	162.79
tr|A0A075B6S3|A0A075B6S	112.83	13	13		3	0	162.79
P61160|ARP2_HUMAN	181.31	40	40	2.87E+07	12	11	232.02
O43795|MYO1B_HUMAN	252.12	38	38	9.99E+07	40	36	302.9
P53675|CLH2_HUMAN	42.62	2	2		2	0	93.69
P17980|PRS6A_HUMAN	20.58	2	2	1.73E+05	1	1	72.42
P21333|FLNA_HUMAN	164.94	14	14	1.54E+07	24	22	217.15
Q09666|AHNK_HUMAN	62.11	1	1	2.33E+07	6	6	115.07
P06493|CDK1_HUMAN	31.61	3	3	2.65E+05	1	1	85.07
O00410|IPO5_HUMAN	59.75	2	2	8.47E+05	2	2	113.6
P25705|ATPA_HUMAN	168	24	24	9.48E+06	10	10	221.99
P46783|RS10_HUMAN	33.75	5	5	1.39E+06	1	1	88.82
Q9Y4L1|HYOU1_HUMAN	27.4	2	2	2.41E+06	2	2	83.03
P30153|2AAA_HUMAN	29.11	2	2	9.71E+04	1	1	84.86
P17987|TCPA_HUMAN	78.98	6	6	2.19E+06	2	2	135.58
Q92747|ARC1A_HUMAN	161.98	36	36	1.54E+07	9	8	218.72
P21964|COMT_HUMAN	108.78	25	25	2.88E+06	5	5	165.71
P35080|PROF2_HUMAN	40.86	16	16	7.65E+05	2	2	97.85
P14923|PLAK_HUMAN	53.48	4	4	9.80E+05	2	2	111.05
Q13200|PSMD2_HUMAN	27.47	2	2	1.45E+05	1	1	85.1
P78527|PRKDC_HUMAN	192.86	12	12	4.30E+07	38	38	251.84
Q16643|DREB_HUMAN	197.69	37	37	2.81E+07	16	16	256.69
P29401|TKT_HUMAN	70.6	6	6	9.78E+05	3	3	129.81
Q9BRS2|RIOK1_HUMAN	33.49	5	5	5.15E+05	2	2	93.18
P78371|TCPB_HUMAN	104.48	16	16	2.51E+06	5	5	166.09
O94906|PRP6_HUMAN	21.82	1	1	1.41E+05	1	1	84.71
P61981|1433G_HUMAN	33.02	9	9	2.47E+05	2	1	97.71
Q12965|MYO1E_HUMAN	83.26	5	5	2.61E+06	4	4	148.52
P78358|CTG1B_HUMAN	58.11	16	16	1.69E+06	2	2	123.42
P05455|LA_HUMAN	32.69	3	3	4.23E+05	1	1	98.81
P04406|G3P_HUMAN	108.86	13	13	4.53E+06	3	3	175.19
tr|A0A075B6S2|A0A075B6S	119.02	19	19	2.93E+06	4	1	186.23
tr|A0A0A0MRZ7|A0A0A0MI	119.02	19	19	2.93E+06	4	1	186.23
Q14697|GANAB_HUMAN	33.18	3	3	3.55E+05	2	2	100.41
P39656|OST48_HUMAN	57.53	5	5	8.87E+05	2	2	125.43
Q9Y4I1|MYO5A_HUMAN	139.93	8	8	9.26E+06	11	8	207.86

P09211|GSTP1_HUMAN	31.79	10	10	1.13E+06	1	1	99.93
P13639|EF2_HUMAN	58.25	5	5	1.12E+06	3	3	126.72
P07910|HNRPC_HUMAN	74.08	10	10	3.56E+06	3	3	142.59
P06733|ENOA_HUMAN	70.76	7	7	8.22E+05	2	1	139.73
Q99733|NP1L4_HUMAN	39.29	7	7	6.09E+05	2	2	109.2
P48643|TCPE_HUMAN	66.87	4	4	1.26E+06	2	1	137.85
Q9P035|HACD3_HUMAN	49.14	6	6	1.48E+06	1	1	120.5
P09327|VILI_HUMAN	193.76	35	35	4.33E+07	25	23	265.38
Q9UQ03|COR2B_HUMAN	52.7	6	6	2.14E+06	3	2	125.3
P09471|GNAO_HUMAN	60.65	15	15	1.09E+06	3	1	134.18
P61158|ARP3_HUMAN	202.02	56	56	6.67E+07	19	19	276.16
Q8IZU2|WDR17_HUMAN	88.73	5	5	1.49E+06	4	4	164.07
Q14195|DPYL3_HUMAN	74.37	8	8	1.37E+06	4	3	151.66
Q9UM54|MYO6_HUMAN	209.23	30	30	5.90E+07	36	36	287.45
P04844|RPN2_HUMAN	95.87	10	10	2.37E+06	5	5	174.51
O15143|ARC1B_HUMAN	139.37	30	30	1.35E+07	10	9	218.58
O43175|SERA_HUMAN	116.91	11	11	3.79E+06	5	4	197.1
O15144|ARPC2_HUMAN	171.48	63	63	2.49E+07	15	15	252.47
P08238|HS90B_HUMAN	116.04	8	8	1.04E+06	5	2	199.06
P35749|MYH11_HUMAN	21.89	1	1		1	0	105.28
P06576|ATPB_HUMAN	146.86	26	26	1.06E+07	10	10	230.78
Q92974|ARHG2_HUMAN	42.84	2	2	5.21E+05	1	1	127.65
P19474|RO52_HUMAN	88.1	15	15	8.76E+06	5	5	173.2
P07900|HS90A_HUMAN	114.18	6	6		4	0	201.6
P10809|CH60_HUMAN	67.25	6	6	3.92E+05	2	2	155.83
P35579|MYH9_HUMAN	134.8	5	5	2.00E+06	9	6	224.7
tr|A0A075B6R9|A0A075B6R	24.1	8	8	1.85E+05	1	1	115.13
O75390|CISY_HUMAN	86.37	12	12	2.93E+06	4	4	178.01
O43707|ACTN4_HUMAN	69.57	3	3	1.32E+06	3	2	161.3
Q14204|DYHC1_HUMAN	75.75	2	2	1.09E+06	5	5	167.85
P08195|4F2_HUMAN	36.78	2	2	5.80E+05	1	1	130.44
O75131|CPNE3_HUMAN	22.5	2	2		1	0	120.07
Q8WWY3|PRP31_HUMAN	79.01	9	9	2.84E+06	3	3	177.01
P05023|AT1A1_HUMAN	30.28	1	1	1.21E+06	1	1	131.78
Q92673|SORL_HUMAN	28.92	1	1	4.80E+05	2	2	130.92
P04843|RPN1_HUMAN	129.06	13	13	4.22E+06	6	6	233.99
P62879|GBB2_HUMAN	30.74	6	6	8.34E+05	2	2	138.9
Q7Z406|MYH14_HUMAN	42.11	1	1		2	0	150.35
P54105|ICLN_HUMAN	86.97	30	30	7.02E+06	3	3	195.25
Q14247|SRC8_HUMAN	56.87	2	2	7.63E+05	2	2	165.5
P35580|MYH10_HUMAN	44.79	1	1		2	0	158.52
P04216|THY1_HUMAN	30.97	9	9	7.24E+04	1	1	144.82
P27824|CALX_HUMAN	51.39	3	3	5.83E+05	1	1	165.78
Q00610|CLH1_HUMAN	71.52	3	3	1.15E+06	4	2	187.65
Q5JWF2|GNAS1_HUMAN	34.4	1	1		1	0	156.99
P08754|GNAI3_HUMAN	63.34	11	11	9.58E+04	3	1	194.46
Q9P219|DAPLE_HUMAN	166.54	14	14	1.37E+07	19	19	312.81
P04899|GNAI2_HUMAN	66.02	11	11	2.66E+05	3	1	219.08
P40939|ECHA_HUMAN	38.59	3	3	0.00E+00	1	1	207.68
