# Supplementary material for: YAP silencing by RB1 mutation is essential for small-cell lung cancer metastasis
Source: Nat Commun. 2023 Sep 22;14:5916. doi: 10.1038/s41467-023-41585-z (PMC10516997; doi:10.1038/s41467-023-41585-z)
Supplement: Supplementary file 3 — Description Of Additional Supplementary File [file 41467_2023_41585_MOESM3_ESM.pdf]

Description of Additional Supplementary Files Document

Supplementary Movie S1

Fast amoeboid migration of controlled H209 cells under confinement

Supplementary Movie S2

Fast amoeboid migration of mScarlet labeled H209 cell in xenografted mice

Supplementary Movie S3

Fast amoeboid migration of EGFP labeled H526 cells in xenografted mice

Supplementary Movie S4

YAP repress amoeboid migration of H209 cells under confinement

Supplementary Movie S5

F-actin is polarized in controlled H209 cells under confinement

Supplementary Movie S6

YAP repress F-actin polarization in H209 cells under confinement
