# Supplementary material for: YAP silencing by RB1 mutation is essential for small-cell lung cancer metastasis
Source: Nat Commun. 2023 Sep 22;14:5916. doi: 10.1038/s41467-023-41585-z (PMC10516997; doi:10.1038/s41467-023-41585-z)
Supplement: Supplementary file 10 — Reporting summary [file 41467_2023_41585_MOESM10_ESM.pdf]

nature portfolio

Corresponding author(s): Kun-liang Guan

Last updated by author(s): Aug 14, 2023

Reporting Summary

Nature Portfolio wishes to improve the reproducibility of the work that we publish. This form provides structure for consistency and transparency in reporting. For further information on Nature Portfolio policies, see our Editorial Policies and the Editorial Policy Checklist.

Statistics

For all statistical analyses, confirm that the following items are present in the figure legend, table legend, main text, or Methods section.

n/a	Confirmed	
☐	☒	The exact sample size (*n*) for each experimental group/condition, given as a discrete number and unit of measurement
☐	☒	A statement on whether measurements were taken from distinct samples or whether the same sample was measured repeatedly
☐	☒	The statistical test(s) used AND whether they are one- or two-sided<br>*Only common tests should be described solely by name; describe more complex techniques in the Methods section.*
☒	☐	A description of all covariates tested
☒	☐	A description of any assumptions or corrections, such as tests of normality and adjustment for multiple comparisons
☐	☒	A full description of the statistical parameters including central tendency (e.g. means) or other basic estimates (e.g. regression coefficient) AND variation (e.g. standard deviation) or associated estimates of uncertainty (e.g. confidence intervals)
☒	☐	For null hypothesis testing, the test statistic (e.g. *F*, *t*, *r*) with confidence intervals, effect sizes, degrees of freedom and *P* value noted<br>*Give P values as exact values whenever suitable.*
☒	☐	For Bayesian analysis, information on the choice of priors and Markov chain Monte Carlo settings
☒	☐	For hierarchical and complex designs, identification of the appropriate level for tests and full reporting of outcomes
☒	☐	Estimates of effect sizes (e.g. Cohen's *d*, Pearson's *r*), indicating how they were calculated

Our web collection on statistics for biologists contains articles on many of the points above.

Software and code

Policy information about availability of computer code

Data collection	The collection of microscopic statistical data were performed using Image J (vl.53); Microscopy image were captured using Nikon Element; Flow cytometry data were performed using FACSDiva(v9.0), All software used in this study are either commercially available or open source
Data analysis	All statistical graphs were performed using the Graph Pad Prism software (vS.00), Flow cytometry data were analyzed using FlowJo (v10.6.1), All software used in this study are either commercially available or open source

For manuscripts utilizing custom algorithms or software that are central to the research but not yet described in published literature, software must be made available to editors and reviewers. We strongly encourage code deposition in a community repository (e.g. GitHub). See the Nature Portfolio guidelines for submitting code & software for further information.

Data

Policy information about availability of data

All manuscripts must include a data availability statement. This statement should provide the following information, where applicable:

- Accession codes, unique identifiers, or web links for publicly available datasets
- A description of any restrictions on data availability
- For clinical datasets or third party data, please ensure that the statement adheres to our policy

he authors declare that all data supporting the findings of this study are available within the article and its Supplementary Information files, or from the

corresponding author upon reasonable request. Source data are provided with this paper. RNA-SEQ and ATAC-SEQ data are available in SRA database under PRJNA752796 [https://www.ncbi.nlm.nih.gov/bioproject/PRJNA752796/].

Research involving human participants, their data, or biological material

Policy information about studies with human participants or human data. See also policy information about sex, gender (identity/presentation), and sexual orientation and race, ethnicity and racism.

Reporting on sex and gender	Nothing to report
Reporting on race, ethnicity, or other socially relevant groupings	Nothing to report
Population characteristics	Nothing to report
Recruitment	Nothing to report
Ethics oversight	Nothing to report

Note that full information on the approval of the study protocol must also be provided in the manuscript.

Field-specific reporting

Please select the one below that is the best fit for your research. If you are not sure, read the appropriate sections before making your selection.

☒ Life sciences ☐ Behavioural & social sciences ☐ Ecological, evolutionary & environmental sciences

For a reference copy of the document with all sections, see nature.com/documents/nr-reporting-summary-flat.pdf

Life sciences study design

All studies must disclose on these points even when the disclosure is negative.

Sample size	Sufficient sample sizes were chosen for each experiment to determine whether the outcome was statistically significant. At least two independent transgenic lines were used for every relevant study, and at least three repetitions were performed for every studies
Data exclusions	No data were excluded from this study.
Replication	We confirmed that all studies performed here is reproducible in all replications.
Randomization	Experimental samples were selected randomly during the experiment without any pre-judgment.
Blinding	Blinding was not implemented in this study.

Reporting for specific materials, systems and methods

We require information from authors about some types of materials, experimental systems and methods used in many studies. Here, indicate whether each material, system or method listed is relevant to your study. If you are not sure if a list item applies to your research, read the appropriate section before selecting a response.

Materials & experimental systems

n/a	Involved in the study
☐	☒ Antibodies
☐	☒ Eukaryotic cell lines
☒	☐ Palaeontology and archaeology
☐	☒ Animals and other organisms
☒	☐ Clinical data
☒	☐ Dual use research of concern
☒	☐ Plants

Methods

n/a	Involved in the study
☒	☐ ChIP-seq
☐	☒ Flow cytometry
☒	☐ MRI-based neuroimaging

Antibodies

Antibodies used	ASCL1 (Abcam, #ab211327), INSM1 (Abcam, #ab170876), E2F7 (Abcam, ab245655), RB1 (Abcam, ab181616), SYP (Santa Cruz, #sc-17750), RCOR1 (Santa Cruz, #sc-376567), CCN1 (Santa Cruz, #sc-374129), CCN2 (Santa Cruz, #sc-365970), YAP/TAZ (Santa Cruz, #sc-101199), P53 (Santa Cruz, #sc-126), E2F1 (Santa Cruz, #sc-251), GAPDH (Santa Cruz, #sc-47724), LATS1 (Cell signaling, #3477), LATS2 (Cell signaling, #5888), p-LATS (Cell signaling, #8654), H3 (Cell signaling, #4499), H3K27ac (Cell signaling, #8173), H3K4me3 (Cell signaling, #9751), VIN (Cell signaling, #13901), Flag (Sigma, #1804),
Validation	All commercial antibodies were validated by the suppliers.

Eukaryotic cell lines

Policy information about cell lines and Sex and Gender in Research

Cell line source(s)	NCI-H526 [H526] is a SCLC cell line that was isolated from the lungs of a 55-year-old, White male with carcinoma. NCI-H69 [H69] is a SCLC cell line from a 55 year white male, NCI-H209 is a SCLC cell line from white male.
Authentication	STR assay were performed for authentication
Mycoplasma contamination	we confirm all cell lines are mycoplasma negative
Commonly misidentified lines (See ICLAC register)	None

Animals and other research organisms

Policy information about studies involving animals; ARRIVE guidelines recommended for reporting animal research, and Sex and Gender in Research

Laboratory animals	Nude mice, Foxn1nu
Wild animals	This study didn't involve wild animals.
Reporting on sex	All nude mice used in this study are male.
Field-collected samples	the study didn't involve samples collected from field.
Ethics oversight	Approval was granted by Animal Experimental Ethical Inspection of Laboratory Centre at Fudan University

Note that full information on the approval of the study protocol must also be provided in the manuscript.

Flow Cytometry

Plots

Confirm that:

☒ The axis labels state the marker and fluorochrome used (e.g. CD4-FITC).

☐ The axis scales are clearly visible. Include numbers along axes only for bottom left plot of group (a 'group' is an analysis of identical markers).

☒ All plots are contour plots with outliers or pseudocolor plots.

☒ A numerical value for number of cells or percentage (with statistics) is provided.

Methodology

Sample preparation	After heparinizing mice, 500 µl of blood was collected into an anticoagulant tube to deplete red blood cells using a lysis solution. The remaining cells were resuspended in 1X HBSS containing 5% BSA. EGFP and mSCARLET-labeled tumor cells were analyzed using GFP and RFP channels, respectively in LSRFortessa X-20 by the UCSD Embryonic Core. The FlowJo software was used for final data processing.
Instrument	LSRFortessa X-20
Software	BDFACSDiva (v9.0), Flow Jo(v10.6.1)
Cell population abundance	No cell sorting were performed in this study.
Gating strategy	For all experiments cell debris were excluded with FSC-A/SSC-A gates and doublets were excluded with FSC-A/FSC-H. Dead cell exclusion (DAPI or 7 AAD negative population) was preformed when appropriate. Positive staining was determined based on FMO and single stains for each experiment

☐ Tick this box to confirm that a figure exemplifying the gating strategy is provided in the Supplementary Information.
